# SB2301-mediated perturbation of membrane composition in lipid droplets induces lipophagy and lipid droplets ubiquitination

**DOI:** 10.1038/s42003-023-04682-9

**Published:** 2023-03-21

**Authors:** Jinjoo Jung, Jongbeom Park, Mingi Kim, Jaeyoung Ha, Hana Cho, Seung Bum Park

**Affiliations:** 1 CRI Center for Chemical Proteomics, Department of Chemistry, Seoul National University, Seoul, 08826 South Korea; 2 Department of Biophysics and Chemical Biology, Seoul National University, Seoul, 08826 South Korea

**Keywords:** Mechanism of action, Small molecules

## Abstract

Lipid droplets (LDs) are involved in various biological events in cells along with their primary role as a storage center for neutral lipids. Excessive accumulation of LDs is highly correlated with various diseases, including metabolic diseases. Therefore, a basic understanding of the molecular mechanism of LD degradation would be beneficial in both academic and industrial research. Lipophagy, a selective autophagy mechanism/LD degradation process, has gained increased attention in the research community. Herein, we sought to elucidate a novel lipophagy mechanism by utilizing the LD-degrading small molecule, SB2301, which activates ubiquitin-mediated lipophagy. Using a label-free target identification method, we revealed that ethanolamine-phosphate cytidylyltransferase 2 (PCYT2) is a potential target protein of SB2301. We also demonstrated that although SB2301 does not modulate PCYT2 function, it induces the cellular translocation of PCYT2 to the LD surface and spatially increases the phosphatidylethanolamine (PE)/phosphatidylcholine (PC) ratio of the LD membrane, causing LD coalescence, leading to the activation of lipophagy process to maintain energy homeostasis.

## Abstract (web summary)

The small molecule SB2301 activates lipophagy and alters lipid droplet membrane composition, with the ethanolamine-phosphate cytidylyltransferase 2 (PCYT2) being a potential target of the compound.

## Introduction

Lipid droplets (LDs) are specialized organelles that store cellular free fatty acids (FFAs) as neutral lipids, such as triacylglycerol (TG) or sterol esters (SE), to avoid FFA lipotoxicity^1,2^. In LDs, neutral lipids are surrounded by phospholipids such as phosphatidylcholine (PC) and phosphatidylethanolamine (PE)^3^, and surface proteins such as the perilipin family^4^ and lipases^5^. Cells generate or degrade LDs dynamically in response to environmental changes to maintain energy homeostasis and regulate lipid metabolism^6^.

Lipases on the LD surface mainly degrade neutral lipids when cells require LD degradation^7,8^. Singh et al. reported that LDs could be a substrate of selective autophagy, called lipophagy, that sequestrates LD within autophagosomes under starvation conditions^9^. The biomedical research community has been working on the lipophagy mechanism to reveal various roles of LDs beyond the storage of neutral lipids^10^, such as cellular stress modulation^11,12^, protein functional regulation^13,14^, and storage of other biomolecules^15,16^. Therefore, studying the LD regulatory mechanism can provide valuable information to define the underlying biological pathways owing to its relevance in chemical biology and drug discovery fields. Furthermore, the physiological relevance of LDs in metabolic diseases has recently been emphasized. The LD accumulation in the muscle and liver is a hallmark of metabolic diseases, including steatosis^17,18^, type 2 diabetes^19,20^, and atherosclerosis^21,22^. Thus, lipophagy has emerged as a new strategy for treating such diseases, especially steatosis and liver diseases^23–25^.

In addition, the phenotype-based approach has been a major strategy for discovering new molecular entities with novel modes of action. In this study, we performed image-based high-thoughput screening to monitor the number and size of cellular LDs as crucial phenotypes in live cells. We further assessed a new small molecule, SB2301, to explore ubiquitin-mediated lipophagy as a new regulatory mechanism of LDs. Using SB2301, we revealed a new lipophagy-activating mechanism that induces LD degradation by spatially altering the lipid composition of the LD membranes.

## Results

### Discovery of LD-reducing small molecules

First, we performed image-based phenotypic screening of cellular LDs in live cells to identify potential modulators of cellular LDs. Previously, we reported the fluorogenic probe, SF44, which has a hydrophobic LD-selective turn-on property in live cells^26^. We also demonstrated the application of SF44 to fluorescence imaging in a high-throughput manner with an excellent Z′ factor^27^. The SF44-based LD monitoring system utilizing SF44 allows real-time observation of the cellular LD dynamics without the need for washing steps. Thus, we applied this high-content LD-monitoring system in live cells to identify new chemical entities lacking cellular toxicity with minimal influence from other external factors.

We previously reported a series of compounds that reduced cellular LDs^28^. These compounds were derived from a privileged substructure-based diversity-oriented synthesis (pDOS) library designed with a central isoxazole moiety and its substitution at the C3 and C5 positions with privileged structures, such as indole, benzopyran, quinoline, and pyrimidine (Supplementary Fig. 1). Among them, only 3-(quinolin-6-yl)phenol-substituted isoxazole derivatives showed excellent efficacy for cellular LD reduction. However, these compounds showed some degree of cytotoxicity. To address this issue, we bioisosterically replaced isoxazole with 1,2,3-triazole as a structural modification to avoid cytotoxicity while improving the desired LD-reducing activities^29^. With 3-(quinolin-6-yl) phenol-substituted isoxazoles as a starting point, we constructed a 1,4-disubstituted 1,2,3-triazoles library containing quinoline-privileged structures for structure-activity relationship (SAR) studies (Supplementary Note 1). A series of 17 analogues were designed and synthesized based on 3-(quinolin-6-yl)phenol and 1,2,3-triazole scaffolds (Supplementary Note 2). As shown in Supplementary Scheme 1, alkynyl quinoline was subjected to a copper(I)-catalyzed click reaction with various aromatic azides in the presence of CuSO_4_·H_2_O and sodium ascorbate.

**Fig. 1:**
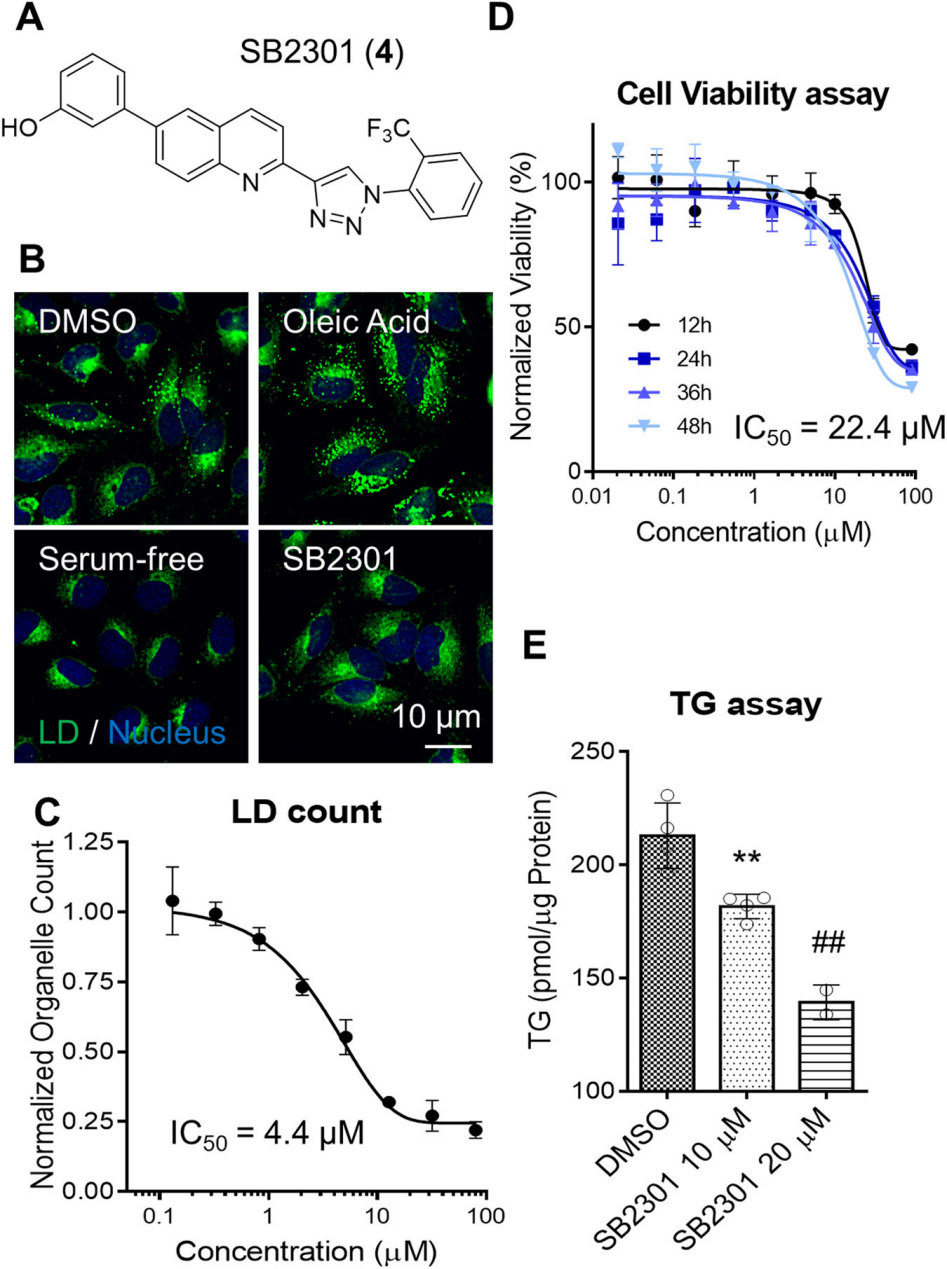
SB2301 reduces cellular LDs without inducing cytotoxicity. **A** Chemical structure of SB2301 (**4**). **B** Representative LD fluorescence images captured during phenotypic screening. **C** The quantification of cellular LD count in HeLa cells upon treatment with various SB2301 doses for 24 h. All data are shown as the mean ± standard deviation (SD). **D** Dose-response curves of cell viability upon SB2301 treatment for the indicated times in HeLa cells. All data are shown as the mean ± SD. **E** TG quantification results upon SB2301 treatment for 24 h in HepG2 cells. All data are represented as dot plots with the mean ± SD, (*n* = 3). Data were analyzed using an unpaired *t*-test. **P = 0.0068 vs. dimethyl sulfoxide (DMSO), ^##^*P* = 0.0029 vs. DMSO.

The LD reduction activity of these analogues (**1**–**20**) was initially evaluated in human cervical cancer HeLa cells at a fixed concentration (10 μM each) to determine the efficacy of LD reduction. In addition, potency was determined by measuring the half-maximal inhibitory concentration (IC_50_) (Supplementary Table 1). Compounds with high cytotoxicity and low water solubility were excluded during high-content phenotypic screening. Based on our SAR study, we confirmed that 1-(2-trifluoromethyl phenyl)-4-(3-hydroxyphenyl quinolone)triazole (**4**) displayed the most potent LD-reducing activity among our derivatives and was named SB2301 (Fig. 1A). We observed a dose-dependent LD reduction activity in HeLa cells with an IC_50_ of 4.4 μM at 24 h after treatment with SB2301 (Fig. 1B, C). Even though SB2301 showed some cytotoxicity at high concentrations (Fig. 1D), we used SB2301 at the concentrations where this is not an issue offering a window of opportunity for the subsequent biological studies. We also observed a similar pattern of LD reduction in human hepatocellular carcinoma HepG2 cells (Supplementary Fig. 2A) and mouse hepatocyte AML12 cells (Supplementary Fig. 2B). Consistent with the reduction in cellular LDs, the cellular triglyceride (TG) content was reduced in a dose-dependent manner (Fig. 1E). Based on these findings, we conducted a further study to determine the mechanism by which SB2301 reduced cellular LDs.

### LD degradation by lipophagy activation

**Fig. 2:**
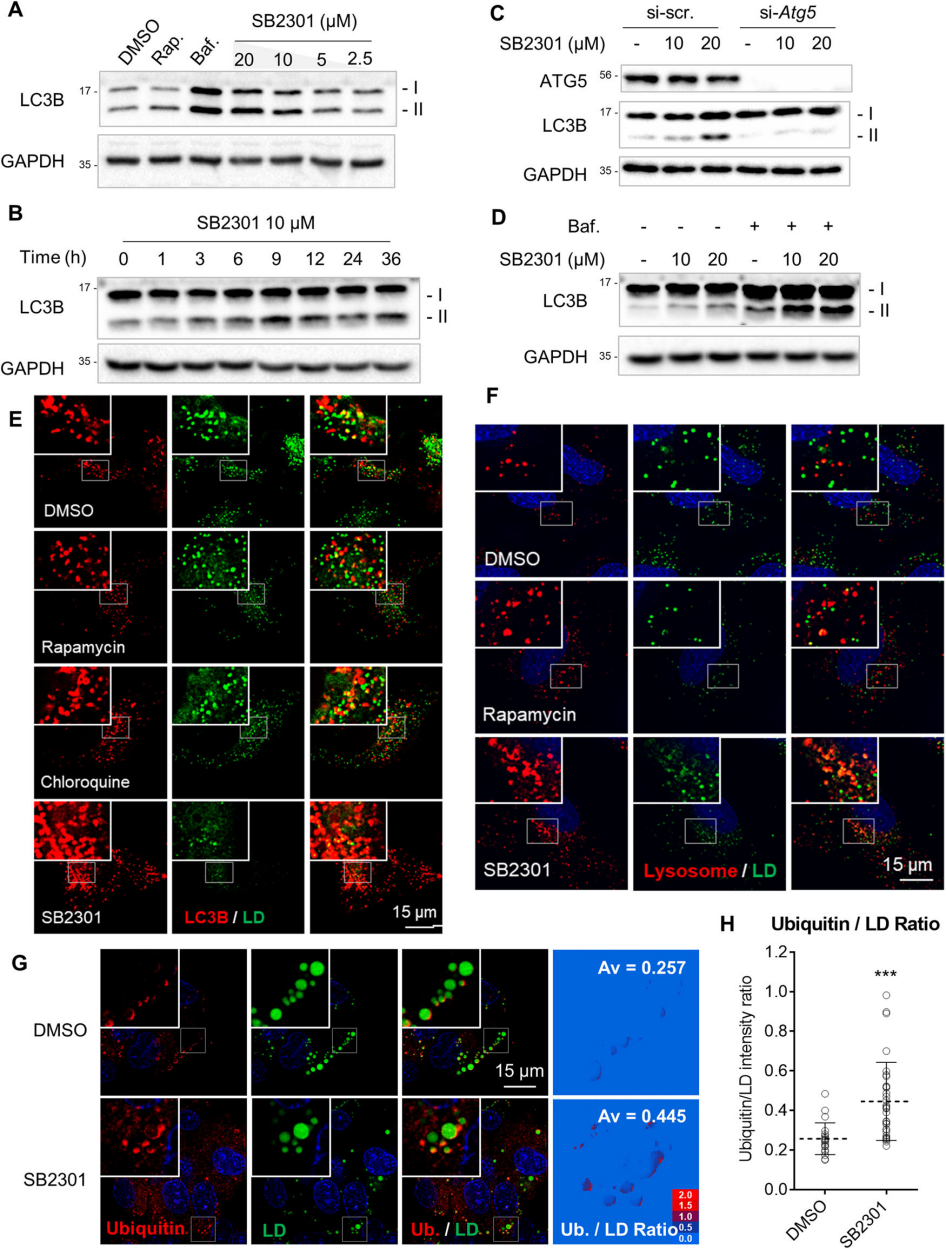
SB2301 reduces cellular LDs via lipophagy. **A** Western blot analysis with various doses of SB2301, rapamycin (Rap, 2 μM), and bafilomycin A1 (Baf, 20 nM) treated in HepG2 cells for 12 h. Western blot analysis with 10 μM of SB2301 for indicated times (**B**), with SB2301 treatment under the autophagy related gene 5 (*Atg5*)-knockdown condition, followed by SB2301 treatment for 9 h (**C**), and with SB2301 treatment in the absence and presence of 5 nM of Baf for 6 h (**D**). **E** Representative live-cell fluorescence images of LC3 (red, mCherry) and LDs (green, SF44) on mCherry-LC3 transfected HeLa cells upon treatment with rapamycin (1 μM), chloroquine (10 μM), and SB2301 (5 μM) for 12 h. **F** Representative live-cell fluorescence images of lysosomes (red, lysotracker) and LDs (green, SF44) upon treatment with rapamycin (1 μM) and SB2301 (10 μM) on HeLa cells for 12 h. **G** Representative immunofluorescence images of ubiquitin (red) and LDs (green, BODIPY 493/503) on HepG2 cells upon SB2301 treatment (10 μM) for 12 h. After cells were fixed, ubiquitin was labeled with anti-ubiquitin antibody and LD was stained with BODIPY 495/503. **H** Fluorescence intensity quantification of images captured in **G**. Images were selected randomly from biological triplicates (*n* = 21 from DMSO, *n* = 32 from SB2301). Cytoplasmic ubiquitin signals were subtracted as a background, and the fluorescent signal ratio (ubiquitin/LD) on each pixel was calculated. All data are represented as dot plots with the mean ± SD. Data were analyzed using an unpaired *t*-test. ***P = 0.0001.

Cellular LD can be reduced either by preventing their formation (anabolic pathway) or promoting their degradation (catabolic pathway). We did not observe any drastic changes in the expression of lipid biosynthesis-related genes upon SB2301 treatment by qPCR analysis (Supplementary Fig. 3A) and proteins regulating TG/CE synthesis by western blot analysis (Supplementary Fig. 3B). Additionally, SB2301-mediated LD reduction was not inhibited by co-treatment with Orlistat, a common lipase inhibitor (Supplementary Fig. 3C). Instead, we confirmed that SB2301 treatment activated LD degradation via lipophagy, a selective autophagic process. During the progression of autophagy, the microtubule-associated protein 1 A/1B-light chain 3 (LC3) I is lipidated to become LC3 II on the surface of the phagophore. According to western blot analysis, LC3 I to II conversion occurred upon SB2301 treatment in both dose- and time-dependent manners (Fig. 2A, B). When we reduced autophagy related gene 5 (ATG5), one of the key proteins for the phagophore elongation of autophagy, SB2301-mediated conversion of LC3 I to II was completely abolished (Fig. 2C). We then examined SB2301-mediated autophagic flux upon treatment with bafilomycin A1 (Baf), a known modulator of vacuolar-type H^+^-ATPase, inhibiting the autolysosomal degradation of autophagic cargo by preventing its acidification, thereby blocking the late-stage autophagic flux. We observed increased LC3 II accumulation upon co-treatment with SB2301 and Baf in a dose-dependent manner (Fig. 2D). We also performed live-cell imaging with the mCherry-GFP-LC3 protein (Supplementary Fig. 4)^30^; Owing to the intrinsic quenching property of GFP in acidic conditions, we could determine whether mCherry-GFP-fused LC3 proteins are located in either autophagosomes (neutral) or autolysosomes (acidic) by simply monitoring changes in the GFP signal while tracking the LC3 protein with a pH-independent mCherry signal. Rapamycin (Rap) activates autophagy by inhibiting the mammalian target of rapamycin complex 1 (mTORC1) signaling pathway and inducing the formation of red puncta due to GFP signal quenching in acidic autolysosomes. In contrast, Baf treatment arrestes the late-stage autophagic flux by blocking lysosomal acidification, which was confirmed by the formation of yellow puncta via live-cell imaging with mCherry-GFP-LC3. Based on our control experiments with the autophagy inducer (Rap) and inhibitor (Baf), SB2301 phenocopied the autophagy inducer, confirming that SB2301 activates autophagy.

We then monitored the cellular location of LD, LC3 (autophagosome), and lysosomes upon SB2301 treatment to determine whether LDs were used as autophagy substrates. As shown in Fig. 2E and Supplementary Fig. 5A, Raf (an autophagy activator, not a lipophagy activator) did not induce LDs to be surrounded by LC3 as LC3-mediated autophagosome substrate selection. Meanwhile, with chloroquine (a lysosomal degradation inhibitor) treatment, LDs were trapped in the autophagosome. SB2301 induced autophagosome formation via autophagy activation, which was consistent with the enhanced fluorescent signal of LC3. Furthermore, SB2301 treatment increased the co-localization of LD with LC3 proteins more than dimethyl sulfoxide (DMSO) control along with a significant reduction in LD fluorescence signals. Collectively, our results indicate that SB2301 induced the sequestration of whole or partial LDs in autophagosomes, which eventually fuse with lysosomes to hydrolyze LDs (Fig. 2F and Supplementary Fig. 5B). To ascertain whether SB2301 induces autophagy in organelles other than cellular LDs, we measured mitochondrial DNA content upon SB2301 treatment and confirmed that mitochondrial contents were unaffected by this treatment (Supplementary Fig. 6), indicating that SB2301 selectively reduced cellular LDs by inducing lipophagy.

Ubiquitination is actively involved in recognizing selective autophagy cargos^31^, such as the mitochondria^32,33^, protein aggregates^34^, peroxisomes^35^, and pathogens^36^. However, ubiquitin-mediated lipophagy has not yet been reported. To determine whether SB2301-mediated LD reduction via lipophagy is regulated by a ubiquitin-mediated mechanism, we performed immunofluorescence imaging against ubiquitin in HepG2 cells. As shown in Fig. 2G, Supplementary Figs. 7, 8, substantial ubiquitination occurred on the LD surface upon SB2301 treatment compared to that with DMSO treatment. In addition, when we quantified the ratio of ubiquitin to the LD signal after background subtraction with cytoplasmic ubiquitin signals, the ratio was significantly higher under SB2301-treated condition (Fig. 2H), confirming that LD surface proteins were more selectively ubiquitinated than cytoplasmic proteins upon SB2301 treatment.

### Target protein identification

To investigate the underlying mechanism of SB2301 on lipophagy, we conducted a target identification study using the thermal stability shift-based fluorescence difference in two-dimensional gel electrophoresis (TS-FITGE). This technique is based on characteristic changes in protein stability induced by thermal denaturation when the protein specifically interacts with its ligand^37^. Briefly, the DMSO- and SB2301-treated cells were subjected to heat shock at increasing temperatures, and the resulting lysates were chemically conjugated with Cy3- and Cy5-*N-*hydroxysuccinimide esters, respectively. Thereafter, two proteomes (fluorescently labeled with Cy3 and Cy5) from each temperature condition were combined and analyzed by 2D gel electrophoresis. Finally, fluorescence image-based analysis of 2D gel revealed color changes of individual protein spots, which were caused by changes in the thermal stability of a protein upon engagement with SB2301.

**Fig. 3:**
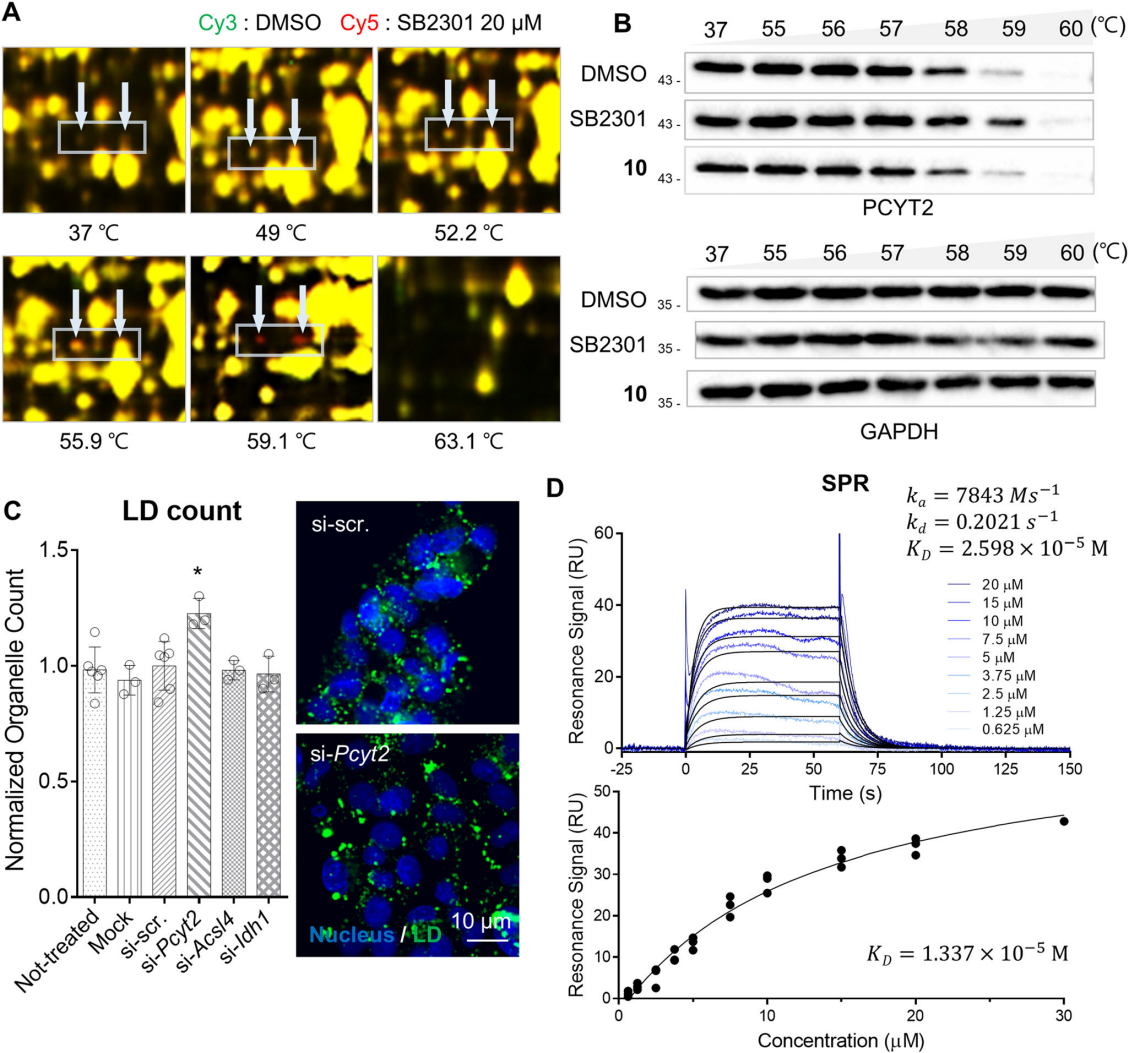
Identification of PCYT2 as a target protein of SB2301 by TS-FITGE. **A** Overlaid images of the Cy3 (green, DMSO-treated proteome) and Cy5 channel (red, SB2301-treated proteome). Indicated spots (white arrow) turned red at 59.1 °C then disappeared at 63.1 °C. **B** Immunoblot from CETSA representing the specific binding of SB2301 with PCYT2. 20 μM of SB2301 and negative compound (**10**) were treated on HepG2 cells for 1 h. **C** LD count changes upon depletion of *Pcyt2*, *Acsl4*, and *Idh1* using the respective siRNAs. HepG2 cells were transfected with siRNAs for 48 h, and then the fluorescent LD images were obtained. All data are represented as dot plots with the mean ± SD, (*n* = 3). Data were analyzed using an unpaired *t*-test. *P = 0.0123 vs. si-scr. **D** Representative sensorgrams of surface plasmon resonance (SPR) analysis of SB2301 binding to human PCYT2. The equilibrium dissociation constant (K_D_) was calculated after 1:1 fitting with kinetic (upper) or affinity mode (lower).

As shown in Fig. 3A and Supplementary Fig. 9, approximately nine red or green spots repeatedly appeared in the 2D-gel images of samples treated at higher temperatures, indicating enhanced (red) or decreased (green) protein thermal stability upon SB2301 engagement. Fourteen protein candidates were identified by mass spectrometry (Supplementary Table 2). A cellular thermal shift assay (CETSA) was performed on several candidate proteins to validate the target ID experiments (Fig. 3B and Supplementary Fig. 10). To prioritize the target protein candidates, we conducted a literature survey on their functions and selected three proteins, PCYT2 (ethanolamine-phosphate cytidylyltransferase 2), ACSL4 (long-chain-fatty-acid-CoA ligase 4), and IDH1 (isocitrate dehydrogenase [NADP] cytoplasmic), which are closely related to cellular lipid metabolism. We then verified whether each target protein could influence the quantity or size of cellular LDs with loss-of-function study. As shown in Fig. 3C and Supplementary Fig. 11, we observed that the cellular level of PCYT2 was inversely correlated with cellular LD counts, suggesting that PCYT2 might influence the lipophagy activation mechanism of SB2301.

We then conducted a biophysical experiment to examine the specific binding of SB2301 to PCYT2 using surface plasmon resonance (SPR) analysis and confirmed the direct engagement of SB2301 with PCYT2 in a 1:1 binding mode (Fig. 3D). Since the estimated equilibrium dissociation constant (Kd) value of SB2301 for PCYT2 was ~26 μM, PCYT2 may not be the sole protein target of SB2301. However, its synthetic analogues (**3** and **10**), which lack LD-reducing activity, showed no thermal stability shift with PCYT2 in CETSA (Supplementary Fig. 12) and no binding events in the SPR analysis (Supplementary Fig. 13A, B). In contrast, in the case of synthetic analogue **14** (moderate LD-reducing activity with cytotoxicity) or **13** (minimal LD-reducing activity), we observed direct binding with PCYT2 with a similar tendency to their LD-reducing activity (Supplementary Fig. 13C, D). Therefore, we concluded that PCYT2 could be a potential target protein correlated with the SB2301-mediated reduction of cellular LDs.

### Spatial regulation of PCYT2 followed by composition changes in LD membrane

**Fig. 4:**
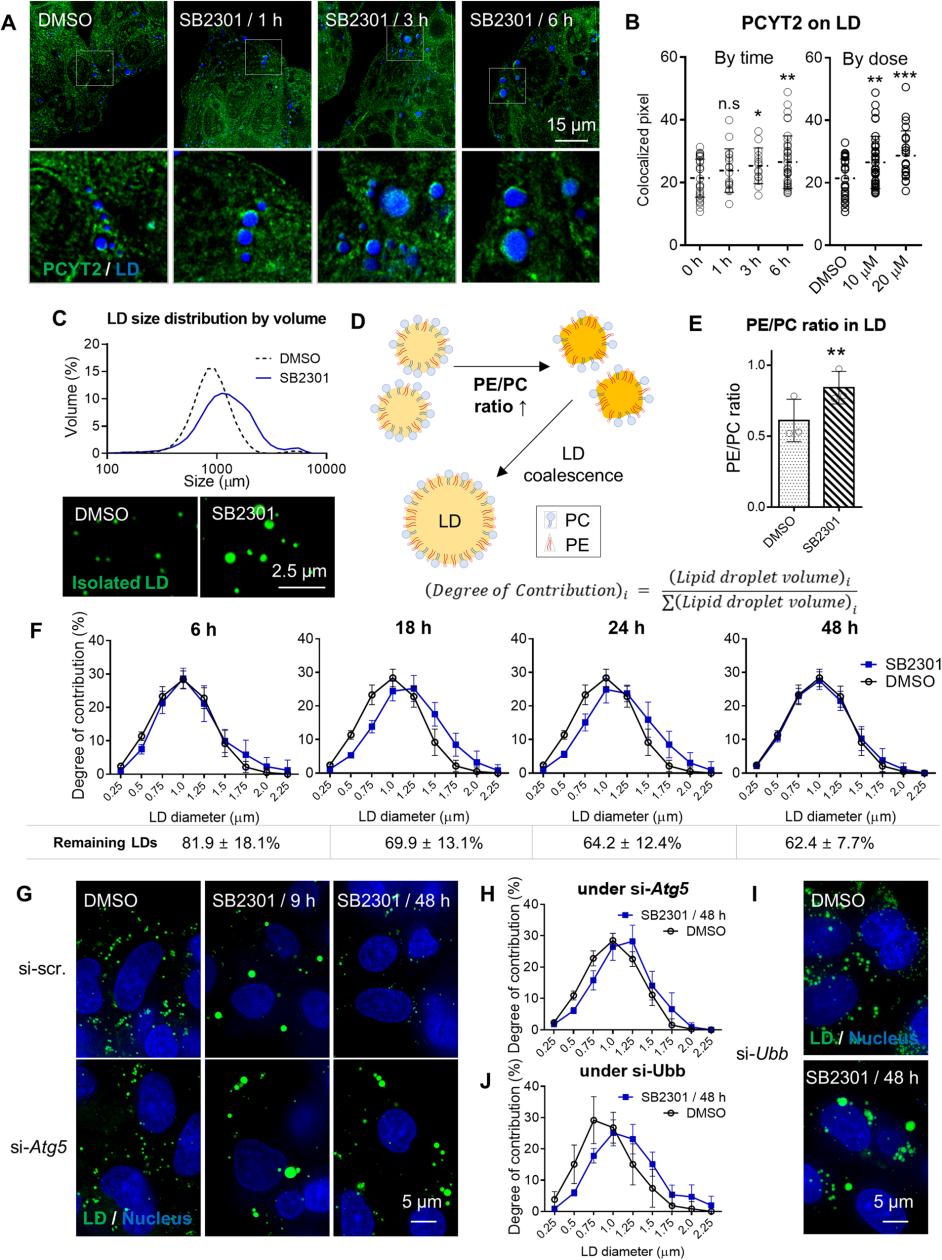
Spatial regulation of PCYT2 followed by dynamic changes in the LD size. **A** Representative immunofluorescence images of PCYT2 (green) and LD (blue, BODIPY 493/503). HepG2 cells were treated with 10 μM SB2301 at the indicated times. The cells were fixed and immunostained for PCYT2 detection. **B** Quantification of PCYT2 on the LD. The superimposed area between LD and PCYT2 on each experimental condition was analyzed using the ImageJ software. Images were selected randomly from biological triplicates. All data are represented as dot plots with the mean ± SD. Data were analyzed using an unpaired *t-*test. *P = 0.0423, **P = 0.0038, ***P = 0.0002. **C** The size distribution of purified LDs was measured by dynamic light scattering. HepG2 cells were treated with 10 μM SB2301 for 9 h prior to the purification of cellular LDs. **D** Schematic illustration of LD coalescence via modulating their PE/PC ratio. **E** The PE/PC ratio measurement in purified LDs shows that SB2301 treatment spatially increases the PE/PC ratio. All data are represented as dot plots with the mean ± SD, (*n* = 3). Data were analyzed using the paired *t-*test. **P = 0.0077. **F** The degree of contribution and remaining LDs were analyzed from LD fluorescence images at each time point. The degree of contribution was calculated according to the definition. The number of remaining LDs was obtained by dividing the total LD area by the cell counts. Upon SB2301 treatment, the mode of the blue graph (the SB2301-treated condition) shifted to the larger LD diameter, then returned to its original value. LD fluorescence images were captured from HepG2 cells treated with 20 μM of SB2301 for the indicated times. All data are represented as the mean ± SD. **G**, **H** Representative LD fluorescence images and quantification results on *Atg5*-knockdown HepG2 cells. ATG5-depleted cells were treated with 20 μM of SB2301 was treated for 48 h. The degree of contribution of LDs was analyzed as described in **E**. All data are represented as the mean ± SD. **I**, **J** Representative LD fluorescence images and quantification results from *Ubb*-knockdown HepG2 cells. UBB-depleted cells were treated with 20 μM of SB2301 for 48 h. The degree of contribution of LDs was then analyzed as described in **E**. All data are represented as the mean ± SD.

PCTY2 is known to catalyze the synthesis of CDP-ethanolamine, a precursor of PE synthesis in the cytosol^38^. Choline/ethanol-amine phosphotransferase (CEPT) synthesizes PE using CDP-ethanolamine on the ER membrane, and the PCYT2-mediated step is the rate-determining step of cellular PE synthesis (Supplementary Scheme 2)^39^. Based on this information, we investigated the changes that might occur when SB2301 binds to PCYT2. Although the enzymatic function of PCYT2 was not affected by SB2301 treatment (Supplementary Fig. 14), we observed translocation of PCYT2 to the LD surface even after a short treatment time (within 1 h) (Fig. 4A, B for SB2301, Supplementary Fig. 15 for DMSO). Furthermore, SB2301 induced translocation of PCYT2 to the LD surface in a time- and dose-dependent manner (Supplementary Fig. 16 and Supplementary Video 1–4). Notably, large-sized LDs began to form within a similar time frame of PCYT2 translocation to the LD surface (Fig. 4A, C, Supplementary Figs. 7A, 17 for SB2301 and Supplementary Fig. 18 for DMSO). Thus, we inferred that LD enlargement might arise with SB2301-induced PCYT2 translocation, which allows for the direct and fast supply of CDP-ethanolamine to the LD surface and the subsequent increase in PE on the LD surface. The main phospholipid components of the LD membrane are PE and PC^3^. PC adopts a cylindrical shape, whereas PE adopts a conical shape owing to its smaller polar head^40^. Due to the different biophysical properties and overall shapes of the two phospholipids, the PE/PC ratio in the LD membrane influences the curvature and size of LD to remain in a state of minimum entropy^40^. Therefore, we hypothesized that SB2301 induces the translocation of PCYT2 at the LD surface, and this spatiotemporal relocation of PCYT2 preferentially increase the levels of PE at the LD surface, which consequently leads to the coalescence of unstable small LDs into large LDs to minimize the surface tension by reducing their curvature (Fig. 4D). We measured the PE/PC ratio in cellular LDs to test this hypothesis. Although we failed to observe any changes in the quantity of PE or PC in the SB2301-treated whole cells (Supplementary Fig. 19), substantial increases in the PE/PC ratio of the isolated LDs were observed (Fig. 4E). Thus, the amount of PE on the LD surface was increased by SB2301-induced PCYT2 translocation, and the resulting PE/PC ratio at the LD surface perturbed its biophysical stability, subsequently leading to LD coalescence.

To elucidate the correlation between lipophagy and PE/PC ratio increase at the LD membrane, we systematically analyzed the dynamic changes in the LD size. The coalescence of small LDs during the earlier period of SB2301 treatment explained the decrease in cellular LD counts without fully activating the autophagy process (Supplementary Fig. 2A). The proportion of large LDs gradually increased around 6 h (Fig. 4A and Supplementary Fig. 16). Within the same period, the autophagy pathway was also activated (Fig. 2B). By analyzing the degree of contribution of cellular LDs over time, we observed a dynamic change in cellular LD volumes (Fig. 4F). Upon SB2301 treatment, LD coalescence occurred for up to 18 h; thereafter, the proportion of large LDs no longer increased and started to decrease owing to the continuous LD degradation. Eventually, the LD size distribution returned to its original value, but the total cellular LD volume was reduced to 64.2% at 24 h (Fig. 4F). However, the LD volume distribution did not return to its original pattern even after SB2301 treatment under *Atg5*- (Fig. 4G, H, and Supplementary Fig. 21 for SB2301; Supplementary Figs. 20, 21 for DMSO) or *Ubb*-knockdown conditions (Fig. 4J, I, and Supplementary Fig. 21 for SB2301; Supplementary Figs. 20, 21 for DMSO). From these observations, we inferred that SB2301 treatment induced the translocation of PCYT2 at the LD surface and that the PE/PC ratio increased at the LD membrane, leading to LD coalescence. LD fusion via a rise in the PE/PC ratio occurred first regardless of autophagy. The resulting unfavorable large LDs may trigger ubiquitin-mediated lipophagy.

### Free fatty acid (FFA) consumption by the mitochondria

**Fig. 5:**
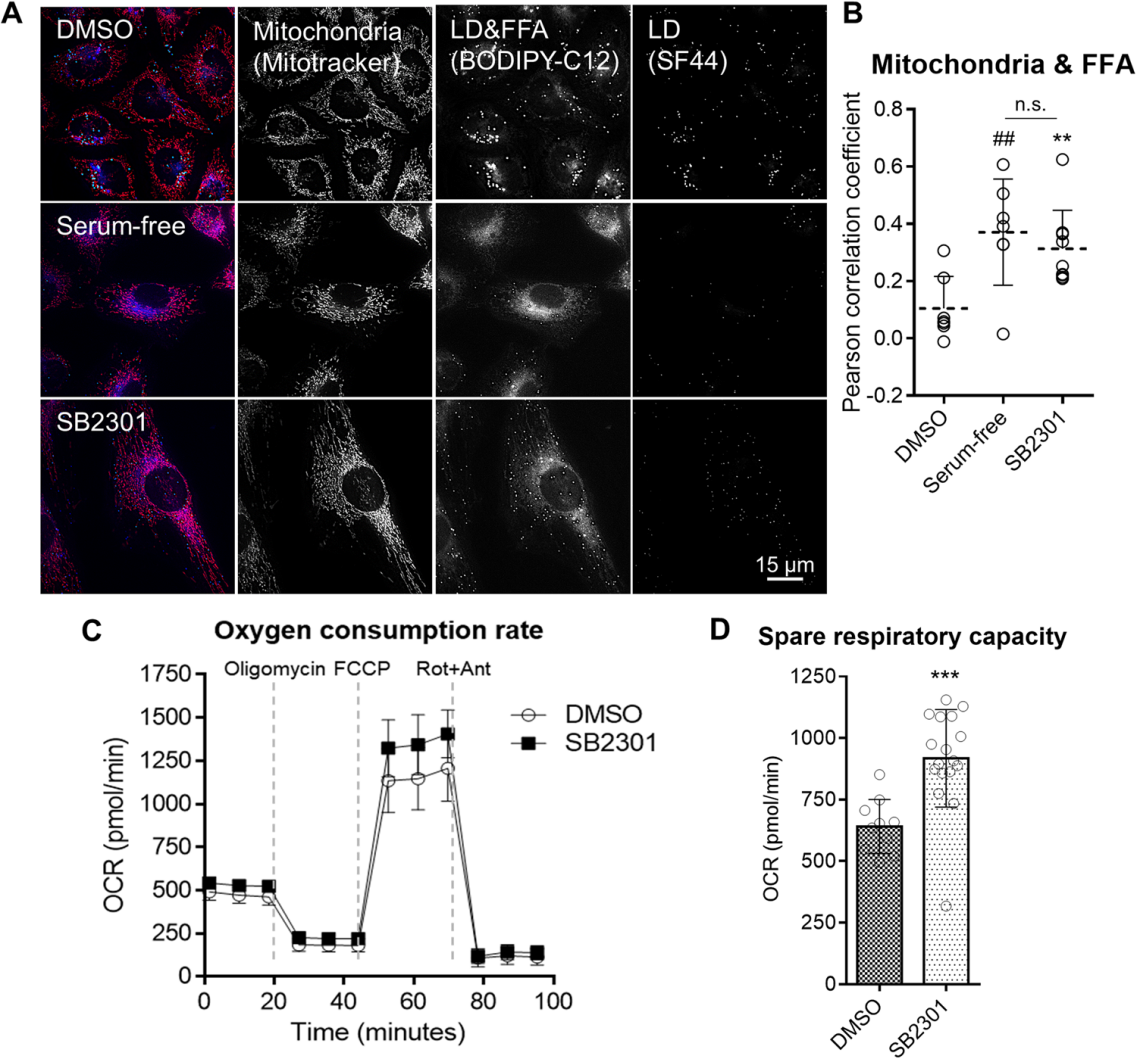
Cellular consumption of FFA is increased upon SB2301 treatment. **A** Representative live-cell fluorescence images of FFA pulse-chase assay with BODIPY-labeled FFA. 2 μM of BODIPY-C12 was treated on HeLa cells for 21 h. The cells were then treated with 5 μM of SB2301 or incubated in a serum-free medium for an additional 24 h. Mitochondria were stained with Mitotracker, and LD was stained with SF44. The images were merged by designating red for mitochondria, blue for BODIPY-C12, and green for LD. **B** Pearson correlation coefficient was calculated to demonstrate the colocalization of BODIPY-C12 and Mitotracker signals. Images were selected randomly from biological triplicates. All data are represented as dot plots with the mean ± SD. Data were analyzed using an unpaired *t*-test. **P = 0.0052 vs. DMSO, ^##^*P* = 0.0070 vs. DMSO. **C** Oxygen consumption rate measurement. AML12 cells were treated with 20 μM of SB2301 for 24 h. All data are represented as the mean ± SD. Rot + Ant; rotenone + antimycin A. **D** Calculated spare respiratory capacity (maximum respiratory capacity—basal respiration) from **C**. All data are represented as dot plots with the mean ± SD, (*n* = 4). Data were analyzed using an unpaired *t*-test. ***P = 0.0004.

We performed a fluorescent-free fatty acid pulse-chase experiment^41^ to determine the fate of FFAs produced by lipophagy. The BODIPY-labeled FFA (BODIPY-C12) is one of the best reagents to track the intracellular movement of FFA from LD simply by monitoring the fluorescence signal of BODIPY-C12. For example, cells with high evergy demands can activate lipophagy and extend mitochondrial β-oxidation of FFAs to produce more ATPs for their survival^42^. When cells were incubated with BODIPY-C12, and then were depleted with serum, more FFAs were found in the mitochondria than in LDs (Fig. 5A, B). Similarly, we observed SB2301 treatment induced the degradation of cellular LDs via lipophagy and produced FFAs that accumulated in the mitochondria for β-oxidation (Fig. 5A, B). We measured the oxygen consumption rate (OCR) of cells in the absence and presence of SB2301 and confirmed that the cells increased their spare respiratory capacity (SRC) in response to SB2301 treatment (Fig. 5C, D, and Supplementary Fig. 22). Cells adjust their metabolic states with enhanced mitochondrial SRC under high energy demands or high-stress conditions to protect themselves from environmental stimuli^43–45^. Similar to the SRC increase, cells respond to environmental stimuli through AMP-activated protein kinase (AMPK), a representative energy sensor, and activate its downstream signaling to regulate β-oxidation^46^; AMPK activation allows the phosphorylation and inactivation of acetyl-CoA carboxylase (ACC), a potent inhibitor of mitochondrial fatty acid oxidation, leading to increased fatty acid oxidation^47,48^. By western blot analysis, we confirmed that AMPK and ACC were phosphorylated by SB2301 (Supplementary Fig. 23). The increase in SRC and AMPK-mediated ACC phosphorylation upon SB2301 treatment indicated that the cells turned on the buffering system to consume or remove the FFAs produced by SB2301-mediated lipophagy, thereby reducing cellular stress such as lipotoxicity.

### Potential application of SB2301 to steatosis disease in a cell culture model

**Fig. 6:**
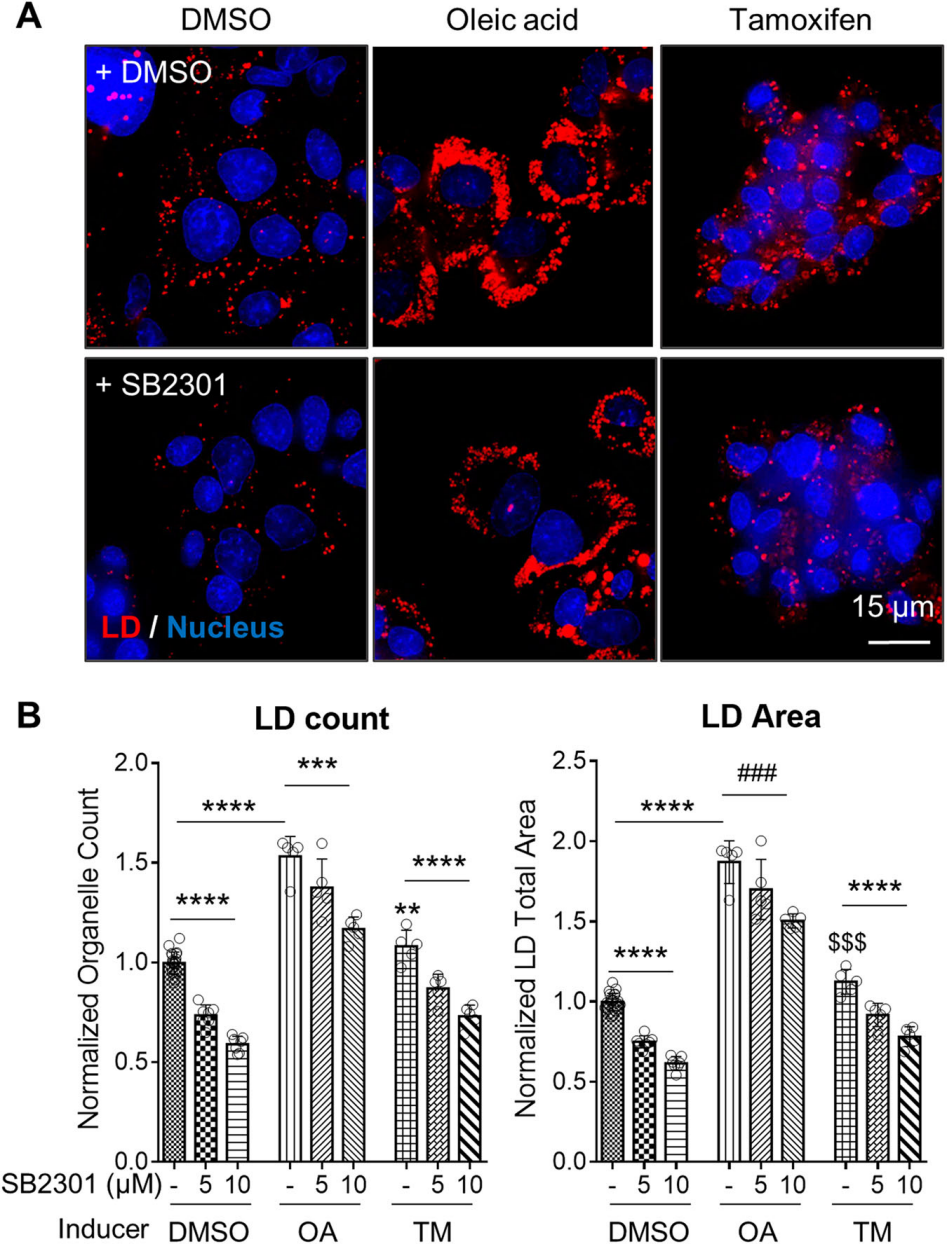
LD-reducing effects on in vitro drug-induced steatosis models. HepG2 cells were treated with 75 μM of oleic acid (OA) or 10 μM of tamoxifen (TM) for 24 h to induce steatosis followed by SB2301 treatment for an additional 24 h. **A** Representative live-cell fluorescence LD images of SB2301 in OA- and TM-treated hepatic steatosis models. **B** Quantification results of cellular LD count and area from **A**. All data are represented as dot plots with the mean ± SD, (*n* = 3). Data were analyzed using an unpaired *t*-test. ****P < 0.0001, ***P = 0.001, ^$$$^*P* = 0.0002 vs. DMSO, ^###^*P* = 0.0004, ^**^P = 0.0093 vs. DMSO.

LD degradation can be a unique and beneficial phenotype for drug discovery as excessive LD accumulation is highly correlated with major causes of various metabolic diseases^18^. In addition, the amount of FFAs regulates non-alcoholic fatty liver diseases^49^. In this context, the mechanism for SB2301-activated lipophagy might be a potential therapeutic strategy as SB2301 effectively reduces LD and consumes FFAs. To explore this novel lipophagy activation mechanism as a therapeutic strategy, we applied SB2301 to in vitro disease models, particularly hepatic steatosis. To develop an in vitro steatosis model, we employed two model systems by treating HepG2 cells with oleic acid (OA)^50^ and tamoxifen (TM)^51^ to mimic a simple high-fat dietary system and severe steatohepatitis system, respectively. In both hepatic steatosis models, SB2301 reduced the cellular LD counts and area in a dose-dependent manner (Fig. 6). These findings confirm that the newly investigated lipophagy-mediated LD-reducing mechanism could be used as a therapeutic strategy for metabolic diseases, including non-alcoholic fatty liver disease and non-alcoholic steatohepatitis.

## Discussion

Cellular LD regulates energy homeostasis and lipid metabolism by changing its quantity and size in response to environmental changes, such as disease states and energy conditions^52^. LD research has gained considerable interest in academia and industry because of its relevance in pathophysiological conditions. However, only a few studies have explored the regulatory mechanisms of cellular LDs, especially by selective autophagy, namely lipophagy. Herein, we discovered a bioactive small molecule, SB2301, that reduces cellular LDs without severe cytotoxicity via a phenotype-based approach. We then demonstrated that SB2301 reduced cellular LDs by activating selective autophagy by monitoring LD sequestration into autophagosomes and autolysosomes, leading to lysosomal degradation. Interestingly, SB2301 treatment promoted ubiquitination on the LD surface, allowing specific substrate recognition for selective autophagy^31,32,35^, although ubiquitinated LDs and their association with lipophagy have not yet been reported. Therefore, we discovered a novel molecular mechanism of intracellular ubiquitin-mediated lipophagy. Several questions remain unanswered, including the identity of ubiquitinated proteins on the LD surface, and the mechanism by which phagophore recognize and sequester the ubiquitinated LDs^53^. Nevertheless, SB2301 combined with quantitative proteomics can serve as a unique research tool to investigate the novel ubiquitin-mediated lipophagy mechanisms.

PCYT2 was identified as a target protein of SB2301 using TS-FITGE. SB2301 treatment induced the translocation of PCYT2 to cellular LDs without affecting its enzymatic activity. We believe that SB2301-mediated translocation of PCYT2 to the LD surface might activate an increase in the PE/PC ratio specifically at LDs, thereby influencing their biophysical stability. As a result, the destabilized LD coalesced to minimize its curvature, and cells activate lipophagy to maintain homeostasis by eliminating unfavorable large LDs. Currently, it is unclear how SB2301 induces the translocation of PCYT2 to the LD surface and needs to conduct the PCYT2 knock-out study to demonstrate the requirement of PCYT2 for the activity of SB2301. However, the results obtained in this study serve as a pilot study to elucidate the detailed molecular mechanism of PCYT2 translocation and ubiquitin-mediated lipophagy processes.

Herein, we showed that SB2301 alters the membrane composition of cellular LDs through the spatial regulation of PCYT2, which directly supplies CDP-ethanolamine to the LD membrane. CEPT mediates the linking of CDP-ethanolamine with diacylglycerol, the final step for PE synthesis, and CEPT only exists on the ER surface^54^. However, it is known that CDP-choline does not necessarily require CEPT to synthesize PC on LD. Furthermore, the translocation of choline-phosphate cytidylyltransferase (PCYT1A) to LD itself can increase the amount of PC and prevent LD fusion^55^. The similarity between PE and PC in their biosynthetic mechanism and spatial synthesis^56^ might support the proposed mechanism in this study. Collectively, SB2301 induced translocation of PCYT2, leading to a spatial increase in the PE/PC ratio in the LD membrane. The resulting biophysical instability of the LD membrane causes LD coalescence and activates ubiquitin-mediated lipophagy to maintain cellular homeostasis. Further proteomics studies for the interactome of the target protein PCTY2 and the identification of ubiquitinated LD surface proteins would be essential to demonstrate how the coalescent LD surface induces protein ubiquitination, activates lipophagy, and identify the key players in this lipophagy-activating mechanism. Nevertheless, this ubiquitin-mediated lipophagy might provide a novel strategy for reducing the intracellular lipid content without inducing lipotoxicity because FFAs produced from degraded LDs can be consumed in mitochondria, as shown in this phenotype-based study. In addition, cellular LD reduction in in vitro hepatic steatosis models was demonstrated as a new therapeutic strategy to treat metabolic diseases caused by fat accumulation, including non-alcoholic steatohepatitis.

## Methods

### Antibodies

Anti-LC3B (ab51520), anti-PCYT2 (ab126142), anti-IDH1 (ab81653), anti-ubiquitin (ab7780), anti-ATG5 (ab108327), TRITC-conjugated anti-rabbit IgG secondary antibodies (ab6718), and anti-DGAT1 (ab178711) were purchased from Abcam. Anti-glyceraldehyde-3-phosphate dehydrogenase (GAPDH) (CST 2118), HRP-labeled anti-mouse IgG (CST 7076), and HRP-labeled anti-rabbit IgG secondary antibodies (CST 7074) were purchased from Cell Signaling Technology. Anti-ACSL4 (sc-271800) and anti-WDR1 (sc-393159) were purchased from SantaCruz. Anti-SOAT1 (Novus biologicals, NB400-141) was purchased from Novus biologicals. Anti-SOAT2 (Cayman, 100027) was purchased from Cayman. Anti-DGAT2 (Thermo, PA5-21722) was purchased from Thermo Scientific.

### Plasmids

mCherry-GFP-LC3 plasmid (pBabe vector) was given from Dr. Heesun Cheong, Division of Chemical Biology, Research Institute, National Cancer Center, Korea. mCherry-LC3 plasmid was purchased from Addgene (40827).

### Imaging instrument

DeltaVision Elite imaging system from GE Healthcare was used for high resolution imaging experiment. Objective lenses were equipped with Olympus IX-71 inverted microscope. sCMOS camera and InSightSSI fluorescence illumination module were equipped with the system. For live cell imaging, a CO_2_ supporting chamber with an objective air heater were installed with the system. Images were analyzed with SoftWorks program supported by GE Healthcare.

### Cell culture

HepG2 cells were cultured in Dulbecco modified eagle medium (DMEM) with 10% (v/v) fetal bovine serum (FBS), and 1% (v/v) antibiotic-antimycotic (AA) solution. HeLa human cervical cancer cells were cultured in RPMI 1640 medium with 10% (v/v) FBS, and 1% AA solution. AML12 cells were cultured in DMEM/F12 (1:1) (Gibco, 11330-032), 1× insulin-transferrin-selenium-G Supplement (Gibco, 41400-045), dexamethasone (final 40 ng/ml), 10% (v/v) FBS, and 1% (v/v) AA solution. Cells were maintained in a 100-mm cell culture dish in an incubator at 37 °C, in a humidified atmosphere with 5% CO_2_.

### LD imaging with SF44

Live cells were treated with SF44 (10 μM) and Hoechst 33342 (2 μg/ml). Serum-free condition (as a positive control) was changed to complete media before treatment with SF44 and Hoechst. After 30-min incubation, automatic fluorescence imaging was performed with InCell Analyzer 2000 [GE Healthcare] or DeltaVision Elite [GE Healthcare] without washing. Using InCell Analyzer 2000, images of four randomly selected spots per individual wells in a 96-well plate were automatically captured. Images were taken in auto-focusing mode at a 20× magnification. Fluorescence imaging was performed using the following filter settings: excitation_emission, 430/24 nm_605/64 nm for LD; 350/50 nm_455/50 nm for nucleus in InCell Analyzer 2000 or 438/24 nm_559/38 nm for LD; 380/18 nm_435/48 nm for nucleus in DeltaVision Elite. According to the manufacturer’s protocol, data were analyzed with InCell Developer program. The fluorescence intensity of LD was interpreted as a cellular organelle using a granularity module, and the area of individual cells was recognized by nuclei staining using collar segmentation.

### Cell viability assay

Cells were seeded in a transparent 96-well plate followed by compound treatment for the indicated times. Cell viability assay was done with WST assay following the manufacturer’s protocol.

### TG assay

HepG2 cells were seeded on a 12-well plate followed by compound treatment for the indicated time. The amount of triglyceride was measured by a triglyceride assay kit (Abcam, ab65336), following the manufacturer’s protocol.

### Mitochondrial DNA extraction and quantification with qPCR

HepG2 cells were treated with SB2301 for indicated times. The genomic DNA was extracted from HepG2 cells with DNeasy DNA extraction kit (Qiagen, 69581) according to the manufacturer’s instructions. 20 ng of genomic DNA was subjected to quantitative PCR (qPCR). KAPA SYBR FAST ABI Prime qPCR master mix (KK4605) and forward/reverse primers (200 nM) against human mitochondrial DNA or human GAPDH were mixed in a final volume of 20 μl with nuclease-free water. The PCR was conducted with StepOnePlus (Applied Biosystems), and the PCR cycling was used; initial denaturing at 95 °C for 3 min, followed by 40 cycles of denaturing at 95 °C for 3 s, and extension at 60 °C for 25 s. The data were analyzed by the comparative CT method, and the mitochondrial DNA levels were normalized to GAPDH levels.

### qPCR analysis of lipid biosynthesis related genes

Total mRNA was isolated from HepG2 cells treated with SB2301 using the RNeasy Plus mini kit (Qiagen 74134). Reverse transcription reaction (RT) was performed with AccuPower Cycle Script RT PreMix (Bioneer, K-2044) with 1 μg of total mRNA in a final volume 20 μl with nuclease-free water in the following conditions: 12 cycles with primer annealing at 30 °C for 1 min and DNA synthesis at 50 °C for 4 min. 1 μl of RT product was subjected to quantitative PCR (qPCR). KAPA SYBR FAST ABI Prime qPCR master mix (KK4605) and forward/reverse primers (200 nM) against each gene were mixed in a final volume 20 μl with nuclease-free water. The PCR was conducted with StepOnePlus (Applied Biosystems), and the PCR cycling was used; initial denaturing at 95 °C for 3 min, followed by 40 cycles of denaturing at 95 °C for 3 s, and extension at 60 °C for 30 s. The data were analyzed by the comparative CT method, and each expression levels were normalized to GAPDH levels.

### Western Blotting

Cells were lysed with radio-immunoprecipitation assay (RIPA) buffer (50 mM Tris, pH 7.8, 150 mM NaCl, 0.5% deoxycholate, 1% IGEPAL CA-630) and protease inhibitor cocktail (Roche). Lysates were obtained after centrifugation at 15,000 rpm for 20 min, by transferring the supernatant. Protein concentration was quantified with BCA protein assay kit. Overall protein sampling procedures were done at 4 °C. Prepared protein samples were analyzed with SDS-PAGE followed by a western blot procedure. Proteins were transferred into the PVDF membrane, and it was blocked with 2% BSA in TBST over 1 h at room temperature (r.t.). Primary antibodies were treated overnight at 4 °C [Anti-LC3B; 1:2000, anti-ATG5; 1:1000, anti-glyceraldehyde-3-phosphate dehydrogenase (GAPDH); 1:2000, anti-DGAT1; 1:1000, anti-DGAT2; 1:1000, anti-SOAT1; 1:500, anti-SOAT2; 1:500] followed by washing with TBST. HRP-labeled anti-rabbit IgG or HRP-labeled anti-mouse IgG secondary antibody (1:5000) were treated at r.t. for 1 h. After washing with TBST, the membrane was developed by Amersham ECL prime solution. The chemiluminescent signal was measured by the ChemiDoc^MP^ imaging system.

### Plasmid transfection

HeLa cells were seeded on Lab-Tek II Chambered Coverglass w/Cover #1.5 Borosilicate Sterile/8 Well (Nunc 155409), 24 h before transfection. mCherry-GFP-LC3 plasmid or mCherry-hLC3 plasmid was transfected to HeLa cells using Lipofectamine 2000 reagent. Transfection was proceeded according to the manufacturer’s protocol.

### mCherry-GFP-LC3 puncta imaging

Fluorescence images from HeLa cells transfected mCherry-GFP-LC3 were obtained with 60× scale, using mCherry/mCherry, GFP/GFP (Excitation/Emission) filter sets. mCherry (excitation: 575/25 nm, emission: 625/45 nm); and GFP (excitation: 475/28 nm, emission: 525/48 nm). Images were analyzed with SoftWorks deconvolution software.

### mCherry-hLC3 and LD imaging

mCherry-hLC3-transfected HeLa cells were treated with SF44 (10 μM) for 30 min before imaging. Fluorescence images were obtained at a magnification of 100× using mCherry/mCherry (excitation_emission, 575/25 nm_625/45 nm) and CFP/YFP (excitation_emission, 438/24 nm_559/38 nm) filter sets. Images were analyzed using the SoftWorks deconvolution software.

### Lysosomes and LD imaging

Before fluorescence imaging, lysosomes were stained with Lysotracker DeepRed (50 nM, Thermo Scientific, L7528) for 1.5 h. LDs were stained with SF44 (10 μM), and nuclei were stained with Hoechst 33342 (2 μg/ml) for 30 min. Images were obtained at a magnification of 100× using Cy5/Cy5, (excitation_emission, 632/22 nm_676/34 nm), FITC/TRITC (excitation_emission, 475/28 nm_594/45 nm), and DAPI/DAPI (excitation_emission, 380/18 nm_435/48 nm). Images were analyzed using the SoftWorks deconvolution software.

### Immunofluorescence

Cells were washed with cold phosphate-buffered saline (PBS) and fixed with 4% paraformaldehyde in PBS. The cells were incubated in 0.1% Triton X-100 in PBS for 15 min at r.t. for permeabilization. The samples were washed three times with ice-cold PBS, followed by incubation with 2% BSA in PBS for 1 h at r.t. Fixed cells on dishes were exposed to the diluted primary antibody solution (ubiquitin; 1:300, PCYT2; 1:200) in PBS with 1% BSA and 3 μM BODIPY 493/503 (Thermo, D3922) at 4 °C overnight. The primary antibody was decanted and washed three times with PBS. Thereafter, a diluted anti-rabbit IgG-TRITC antibody (1:200) solution with 3 μM BODIPY 493/503 and Hoechst 33342 (2 μg/ml) was applied to the samples and incubated at r.t. for 1 h. After washing three times with PBS, fluorescence images were captured using DeltaVision Elite fluorescence microscopy (100×) using TRITC/TRITC, (excitation_emission, 542/27 nm_594/45 nm), FITC/FITC (excitation_emission, 475/28 nm_525/48 nm), and DAPI/DAPI (excitation_emission, 380/18 nm_435/48 nm). Images were analyzed using the SoftWorks deconvolution software.

### TS-FITGE

Human hepatocellular carcinoma HepG2 cells were incubated in serum-free DMEM with 20 μM SB2301 (0.2% (v/v) of DMSO in final concentration) for 1 h at 37 °C. Heat shock was applied to the cells for 3 min. The resulting cells were stabilized at 25 °C for 3 min, washed once with PBS, and resuspended in PBS containing 0.4% NP-40 and protease inhibitor. Cells were subjected to three freeze(liquid nitrogen)/thaw cycles for cell lysis. The cell lysate was clarified by centrifugation at 20,000 g for 20 min at 4 °C. 50 mg of protein was precipitated, and the residual pellet was resuspended in 10 μl of labeling buffer (30 mM Tris-HCl at pH 8.6, 2 M thiourea, 7 M urea, and 4% (w/v) CHAPS) with sonication. The soluble proteomes were mixed with 0.4 mM Cy3-NHS (for DMSO-treated group) or Cy5-NHS (for SB2301-treated group), and incubated at 4 °C for 45 min. The dye-conjugated proteomes were precipitated with cold acetone and resuspended in 75 μl of rehydration buffer (7 M urea, 2 M thiourea, 2% (w/v) CHAPS, 40 mM DTT, and 1% IPG buffer). DMSO-treated SB2301-treated samples were mixed, and 150 μl (75 μl for Cy3- and Cy5-labeled each) of proteomes was loaded on a 24-cm Immobiline Drystrip gel [GE Healthcare]. Isoelectric focusing was performed using Ettan IPGphor 3 [GE Healthcare] followed by 2-dimensional SDS-PAGE with an Ettan DALTsix system [GE Healthcare]. The gel was scanned using a Typhoon Trio [GE Healthcare]. For LCMS/MS, the gel was excised after silver staining.

### In-gel digestion and mass spectrometry

The protein spots from the silver-stained gel were excised, destained, and digested with trypsin. The mixture was evaporated in SpeedVac and then dissolved in 10% acetonitrile with 0.1% formic acid. The resulting peptides were desalted in a trap column (180 μm × 20 mm, Symmetry C18) and separated on a C18 reversed-phase analytical column (75 μm × 200 mm, 1.7 μm, BEH130 C18) [Waters] with an electrospray ionization Pico Tip (±10 μm i.d.) [New objective]. The data were converted to.pkl files by Protein Lynx Global Server and searched by MASCOT engine with the SwissProt database.

### CETSA

Trypsinized HepG2 cells were incubated in serum-free DMEM media with the 20 μM compound (final 0.2% (v/v) DMSO concentration) for 1 h at 37 °C. Heat shock applied to cell for 3 min and cells were stabilized at 25 °C for 3 min. Wash cell with PBS once and resuspend cells with PBS containing 0.4% NP-40 and protease inhibitor. Lysis cells with freeze (liquid nitrogen)/thaw cycle, three times. Prepare soluble proteins and detect protein quantity with western blotting. (Primary antibody dilution/anti-ACSL4; 1:1000, anti-PCYT2; 1:1000, anti-IDH1; 1:1000, anti-WDR1; 1:200, anti-GAPDH; 1:2000).

### si-RNA treatment

20 μM of si-RNA was applied to HepG2 cells for 48 h with Lipofectamine RNAiMAX (Invitrogen, 13778-100) following manufacture’s protocols.

### SPR analysis

The equilibrium dissociation constant (Kd) toward PCYT2 was determined by SPR using a Biacore T100 instrument [GE Healthcare]. The carboxyl group on the surface of the CM5 sensor chip was replaced with reactive succinimide ester using a combination of 1-ethyl-3-(3-dimethylaminopropyl)-carbodiimide (EDC) and *N-*hydroxysuccinimide (NHS) in flow cells 1 and 2. Human PCYT2 (Prospec, enz-221) was immobilized on flow cell 2 (10000 RU) through the formation of amide bonds with the resulting NHS ester. The remaining NHS ester on flow cells 1 and 2 was quenched via injection with 1 M ethanolamine-HCl (pH 8.0) solution. During the immobilization process, PBS was used as the running buffer. After immobilization, different concentrations of the compounds were injected for 60 s at a flow rate of 10 μL·min^−1^. At the same flow rate, the dissociation from the sensor surface was monitored for 300 s. For the running buffer, we used 1× PBS (pH 7.3) containing 3% DMSO and 0.05% P20. The binding events were measured at 25 °C. Data analysis was performed using the Biacore T100 Evaluation software [GE Healthcare]. Final sensorgrams were obtained after the elimination of responses from flow cell 1 and buffer-only control. The Kd was calculated by fitting the sensorgrams to a 1:1 binding model.

### PCYT2 enzymatic assay

PCYT2 activity was assayed as described previously with minor modification^57^. Briefly, prepare reaction mixture (50 μl) in 20 mM Tris-HCl buffer (pH 7.8), 5 mM DTT, 10 mM MgCl2, 650 μM phosphoethanolamine, and CTP with various concentration. Then the reaction mixture was incubated with 0.1 μg of purified PCYT2 at 37 °C for 3 min. Reactions were terminated by boiling for 2 min. The concentration of product (pyrophosphate) was measured by pyrophosphate assay kit (Lonza, LT07-610), and the reaction rate was calculated. DMSO and SB2301 were added at the indicated concentration in the reaction mixture.

### Quantification of the superimposed area between LD and PCYT2

Images were analyzed with ImageJ software [National Institutes of Health, Bethesda, MD, USA]. Background images of PCYT2 channel were obtained from Gaussian blur using Radius 8, and the original images of PCYT2 channel were subtracted from the background images to correct for inhomogeneous background. LD mask was prepared by adjusting the LD signal according to an intensity threshold (Image > adjust > threshold) using the default algorithm. For the LD edge mask, Find edges (Process > Find edges) was used to the thresholded LD images. The superimposed area between PCYT2 of which background was corrected and LD mask or LD edge mask was quantified by Image calculator (Process > Image calculator) using AND operation. The resulting images were thresholded, and the area was quantified by Analyze particles (Analyze > Analyze particles) using a size filter of 5-infinity (pixel).

### LD fractionation

LDs were isolated from HepG2 cell (~10^8^ cells) using LD isolation kits (Cell Biolabs, MET-5011) according to the manufacturer’s instructions. The membrane, cytosol, and LD fraction were collected for purity check. 1 ml of chloroform/acetone (1:1, v/v) was added to separate proteins and lipids from the isolated LDs. Next, the organic phase was collected for further phospholipid composition analysis. Repeat this process twice with the pellet to dissolve the lipids. After removing the organic phase, the pellet was dried at r.t. and resuspended with SDS sampling buffer to prepare protein samples for purity check.

### LD size distribution by volume

Dynamic light scattering data was obtained with purified LDs in PMMA cuvette (Ratiolab, 2810100) by Malvern Zetasizer Nano-S. Dispersant and temperature were selected as water and 25 °C, respectively.

### PE/PC ratio analysis

To measure the amount of PE and PC in the lipid sample, PE assay kit (Biovision, K499) and PC assay kit (Biovision, K576) were used. Extracted lipids from whole cell lysates or purified cellular LDs were prepared via solvent evaporation using a rotary evaporator. According to the manufacturer’s instructions, dried lipids reconstituted with PE assay buffer and PC assay buffer were employed to measure each quantity.

### LD volume and size distribution analysis

Ten fluorescence images were captured at one point with 0.8 µm z-depth. Projected images were generated using SoftWorks deconvolution software. Fifteen randomly selected images from each condition were analyzed using InCell Developer [GE Healthcare], and the diameters of all LDs present in the given image were quantified. Assuming that the LD is a perfect sphere, the volume of each lipid droplet was calculated using the relationship between the radius and volume (*V* = 4/3 πr^3^). The LDs were divided into nine groups according to the length of the radius. The LD size distribution was calculated by dividing the sum of the volumes of LDs corresponding to each group by the sum of the volumes of total LDs.

### Fluorescent FFA pulse-chase experiments

HeLa cells were incubated with 2 μM BODIPY 558/568 C12 (Thermo, D3835) in complete media for 21 h. Cells were then washed twice with complete media, incubated for an additional 1 h without BODIPY 558/568 C12. SB2301 was treated for 24 h. For positive control, serum-starved (16 h) cells were prepared, separately. Mitochondria were labeled with 100 nM MitoTracker DeepRed (Thermo, M22426), and LDs were labeled with 10 μM SF44 for 30 min simultaneously prior to fluorescence imaging. Live cell imaging was conducted within 2 h.

### OCR measurement

AML12 cells were seeded on an Agilent Seahorse XFe24 plate and subsequently treated with SB2301. A sensor cartridge with Seahorse XF Calibrant (Agilent, 100840-000) was hydrated at 37 °C overnight in a non-CO_2_ incubator. OCR was measured in Seahorse XF DMEM (Agilent, 103757-100) containing 17 mM glucose (Agilent, 103577-100), 2.5 mM glutamine (Agilent, 103579-100), and 0.5 mM pyruvate (Agilent, 103578-100) in response to 1.5 μM oligomycin, 1 μM fluorocarbonylcyanide phenylhydrazone (FCCP), and 0.5 μM rotenone + antimycin A (Seahorse XF Cell Mito Stress Test Kit, 103015-100) with Seahorse XFe24 [Agilent].

### Statistics and reproducibility

The type of used statistical test and the number of biological replicates are specified in each figure’s legend. The figures of each experiment from each biological replicate can be found in the Supplementary Data 1.

### Reporting summary

Further information on research design is available in the Nature Portfolio Reporting Summary linked to this article.

## Supplementary information

Supplementary Information

Description of Additional Supplementary Files

Supplementary Data 1

Supplementary Video 1

Supplementary Video 2

Supplementary Video 3

Supplementary Video 4

Reporting Summary

## Data Availability

Target identification proteomics results (peak files and search files) can be found in 10.6084/m9.figshare.22215583.v1. The results of each biological replicate are provided in Supplementary Data 1. Other data generated or analyzed in this study are provided in this article or its supplementary information files (including the uncropped western blots in Supplementary Fig. 24).

## References

[1] Olzmann JA, Carvalho P (2019). Dynamics and functions of lipid droplets. Nat. Rev. Mol. Cell Biol..

[2] Bailey Andrew P (2015). Antioxidant role for lipid droplets in a stem cell niche of Drosophila. Cell.

[3] Penno A, Hackenbroich G, Thiele C (2013). Phospholipids and lipid droplets. Biochim. Biophys. Acta - Mol. Cell Biol. Lipids.

[4] Itabe H, Yamaguchi T, Nimura S, Sasabe N (2017). Perilipins: a diversity of intracellular lipid droplet proteins. Lipids Health Dis.

[5] Bersuker K (2018). A proximity labeling strategy provides insights into the composition and dynamics of lipid droplet proteomes. Dev. Cell.

[6] Welte MA (2015). Expanding roles for lipid droplets. Curr. Biol..

[7] Rizack MA (1964). Activation of an epinephrine-sensitive lipolytic activity from adipose tissue by adenosine 3′,5′-phosphate. J. Biol. Chem.

[8] Vaughan M, Steinberg D (1963). Effect of hormones on lipolysis and esterification of free fatty acids during incubation of adipose tissue in vitro. J. Lipid Res..

[9] Singh R (2009). Autophagy regulates lipid metabolism. Nature.

[10] Welte MA, Gould AP (2017). Lipid droplet functions beyond energy storage. Biochim. Biophys. Acta Mol. Cell Biol, Lipids.

[11] Bosma M (2014). Sequestration of fatty acids in triglycerides prevents endoplasmic reticulum stress in an in vitro model of cardiomyocyte lipotoxicity. Biochim. Biophys. Acta. Mol. Cell Biol. Lipids.

[12] Bensaad K (2014). Fatty acid uptake and lipid storage induced by HIF-1α contribute to cell growth and survival after hypoxia-reoxygenation. Cell Rep.

[13] Moldavski O (2015). Lipid droplets are essential for efficient clearance of cytosolic inclusion bodies. Dev. Cell.

[14] Camus G (2013). Diacylglycerol acyltransferase-1 localizes Hepatitis C virus NS5A protein to lipid droplets and enhances NS5A interaction with the viral capsid core. J. Biol. Chem..

[15] Spicher L, Kessler F (2015). Unexpected roles of plastoglobules (plastid lipid droplets) in vitamin K1 and E metabolism. Curr. Opin. Plant Biol..

[16] Papackova Z, Cahova M (2015). Fatty acid signaling: the new function of intracellular lipases. Int. J. Mol. Sci..

[17] Fabbrini E, Sullivan S, Klein S (2010). Obesity and nonalcoholic fatty liver disease: Biochemical, metabolic, and clinical implications. Hepatology.

[18] Gluchowski NL, Becuwe M, Walther TC, Farese RV (2017). Lipid droplets and liver disease: from basic biology to clinical implications. Nat. Rev. Gastroenterol. Hepatol..

[19] Loon LJCV (2004). Intramyocellular lipid content in type 2 diabetes patients compared with overweight sedentary men and highly trained endurance athletes. Am. J. Physiol. Endocrinol. Metab..

[20] McGavock JM (2007). Cardiac steatosis in diabetes mellitus: a 1H-magnetic resonance spectroscopy study. Circulation.

[21] Chiu H-C (2001). A novel mouse model of lipotoxic cardiomyopathy. J. Clin. Invest..

[22] Larigauderie G (2004). Adipophilin enhances lipid accumulation and prevents lipid efflux from THP-1 macrophages: potential role in atherogenesis. Asterioscler, Thromb, Vasc. Biol.

[23] Zubiete-Franco I (2016). Methionine and S-adenosylmethionine levels are critical regulators of PP2A activity modulating lipophagy during steatosis. J. Hepatol..

[24] Kurahashi T (2015). An SOD1 deficiency enhances lipid droplet accumulation in the fasted mouse liver by aborting lipophagy. Biochem. Biophys. Res. Commun..

[25] Zhang Z (2018). Lipophagy and liver disease: New perspectives to better understanding and therapy. Biomed. Pharmacother..

[26] Kim E, Lee S, Park SB (2012). A Seoul-Fluor-based bioprobe for lipid droplets and its application in image-based high throughput screening. Chem. Commun..

[27] Lee S, Kim E, Park SB (2013). Discovery of autophagy modulators through the construction of a high-content screening platform via monitoring of lipid droplets. Chem. Sci..

[28] Kim M, Hwang YS, Cho W, Park SB (2017). Synthesis of 3,5-disubstituted isoxazoles containing privileged substructures with a diverse display of polar surface area. ACS Comb. Sci..

[29] Patani GA, LaVoie EJ (1996). Bioisosterism: A rational approach in drug design. Chem. Rev..

[30] Klionsky DJ (2021). Guidelines for the use and interpretation of assays for monitoring autophagy (4th edition)1. Autophagy.

[31] Kirkin V, McEwan DG, Novak I, Dikic I (2009). A role for ubiquitin in selective autophagy. Mol. Cell.

[32] Koyano F (2014). Ubiquitin is phosphorylated by PINK1 to activate parkin. Nature.

[33] Kane LA (2014). PINK1 phosphorylates ubiquitin to activate Parkin E3 ubiquitin ligase activity. J. Cell Biol.

[34] Lu K, Psakhye I, Jentsch S (2014). Autophagic clearance of PolyQ proteins mediated by ubiquitin-Atg8 adaptors of the conserved CUET protein family. Cell.

[35] Kim PK, Hailey DW, Mullen RT, Lippincott-Schwartz J (2008). Ubiquitin signals autophagic degradation of cytosolic proteins and peroxisomes. Proc. Natl. Acad. Sci..

[36] Thurston TLM, Ryzhakov G, Bloor S, von Muhlinen N, Randow F (2009). The TBK1 adaptor and autophagy receptor NDP52 restricts the proliferation of ubiquitin-coated bacteria. Nat. Immunol..

[37] Park H, Ha J, Koo JY, Park J, Park SB (2017). Label-free target identification using in-gel fluorescence difference via thermal stability shift. Chem. Sci..

[38] Bakovic M, Fullerton MD, Michel V (2007). Metabolic and molecular aspects of ethanolamine phospholipid biosynthesis: the role of CTP:phosphoethanolamine cytidylyltransferase (Pcyt2). Biochem. Cell Biol..

[39] Sundler R (1975). Ethanolaminephosphate cytidylyltransferase. Purification and characterization of the enzyme from rat liver. J. Biol. Chem.

[40] Thiam AR, Farese RV, Walther TC (2013). The biophysics and cell biology of lipid droplets. Nat. Rev. Mol. Cell Biol..

[41] Rambold Angelika S, Cohen S, Lippincott-Schwartz J (2015). Fatty acid trafficking in starved cells: Regulation by lipid droplet lipolysis, autophagy, and mitochondrial fusion dynamics. Dev. Cell.

[42] Liu K, Czaja MJ (2013). Regulation of lipid stores and metabolism by lipophagy. Cell Death Differ.

[43] van der Windt Gerritje JW (2012). Mitochondrial respiratory capacity is a critical regulator of CD8+ T cell memory development. Immunity.

[44] Yamamoto H (2016). Amla enhances mitochondrial spare respiratory capacity by increasing mitochondrial biogenesis and antioxidant systems in a murine skeletal muscle cell line. Oxid. Med. Cell. Longev..

[45] Choi SW, Gerencser AA, Nicholls DG (2009). Bioenergetic analysis of isolated cerebrocortical nerve terminals on a microgram scale: spare respiratory capacity and stochastic mitochondrial failure. J. Neurochem.

[46] Hardie DG, Ross FA, Hawley SA (2012). AMPK: a nutrient and energy sensor that maintains energy homeostasis. Nat. Rev. Mol. Cell Biol..

[47] Ruderman NB, Saha AK, Vavvas D, Witters LA (1999). Malonyl-CoA, fuel sensing, and insulin resistance. Am. J. Physiol. Endocrinol. Metab..

[48] Zang MW (2004). AMP-activated protein kinase is required for the lipid-lowering effect of metformin in insulin-resistant human HepG2 cells. J. Biol. Chem..

[49] Schweiger M (2017). Pharmacological inhibition of adipose triglyceride lipase corrects high-fat diet-induced insulin resistance and hepatosteatosis in mice. Nat. Commun..

[50] Cui W, Chen SL, Hu KQ (2010). Quantification and mechanisms of oleic acid-induced steatosis in HepG2 cells. Am. J. Transl. Res..

[51] Zhao F (2014). The effect and mechanism of tamoxifen-induced hepatocyte steatosis in vitro. Int. J. Mol. Sci..

[52] Henne WM, Reese ML, Goodman JM (2018). The assembly of lipid droplets and their roles in challenged cells. EMBO J.

[53] Gatica D, Lahiri V, Klionsky DJ (2018). Cargo recognition and degradation by selective autophagy. Nat. Cell Biol..

[54] Henneberry AL, Wright MM, McMaster CR (2002). The major sites of cellular phospholipid synthesis and molecular determinants of fatty acid and lipid head group specificity. Mol. Biol. Cell.

[55] Krahmer N (2011). Phosphatidylcholine synthesis for lipid droplet expansion is mediated by localized activation of CTP:Phosphocholine cytidylyltransferase. Cell Metab.

[56] Henneberry AL, Wistow G, McMaster CR (2000). Cloning, genomic organization, and characterization of a human cholinephosphotransferase. J. Biol. Chem..

[57] Gohil VM (2013). Meclizine inhibits mitochondrial respiration through direct targeting of cytosolic phosphoethanolamine metabolism. J. Biol. Chem.

