# Supplementary material for: SB2301-mediated perturbation of membrane composition in lipid droplets induces lipophagy and lipid droplets ubiquitination
Source: Commun Biol. 2023 Mar 21;6:300. doi: 10.1038/s42003-023-04682-9 (PMC10030462; doi:10.1038/s42003-023-04682-9)
Supplement: Supplementary file 1 — Supplementary Information [file 42003_2023_4682_MOESM1_ESM.pdf]

< Supplementary Information >

SB2301-mediated perturbation of membrane composition in lipid droplets induces lipophagy and lipid droplets ubiquitination

Jinjoo Jung,[1] Jongbeom Park,[1] Mingi Kim,[1] Jaeyoung Ha,[2] Hana Cho,[2] and Seung Bum Park[1,2,]*

[1]CRI Center for Chemical Proteomics, Department of Chemistry, Seoul National University, Seoul 08826, South Korea
[2]Department of Biophysics and Chemical Biology, Seoul National University, Seoul 08826, South Korea
sbpark@snu.ac.kr

S. No.	Content	Page No.
I	Supplementary Figures	S2
II	Supplementary Notes	S32
III	Supplementary Tables	S39
VI	Copies of ^{1}H and ^{13}C NMR Spectra	S42
V	Supplementary Reference	S59

I. Supplementary Figures

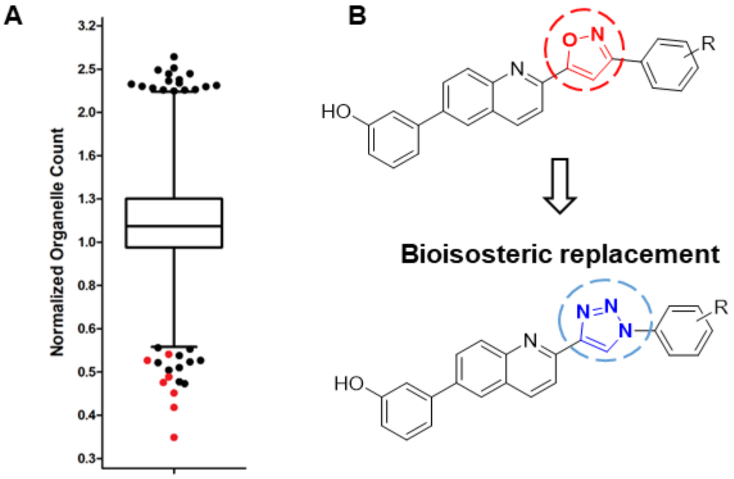

Supplementary Figure 1. Discovery of initial hit compounds and lead optimization strategy. (A) Discovery of initial hit compounds, 3-(quinolin-6-yl)phenol-substituted isoxazoles (red spots), by high-contents screening against 3000 in-house small molecule library to monitor cellular LDs. Box plot shows the distribution of LD count upon compound treatment. The whiskers indicate 1–99 percentiles. (B) Bioisosteric replacement strategy of isoxazoles to triazoles and its library construction.

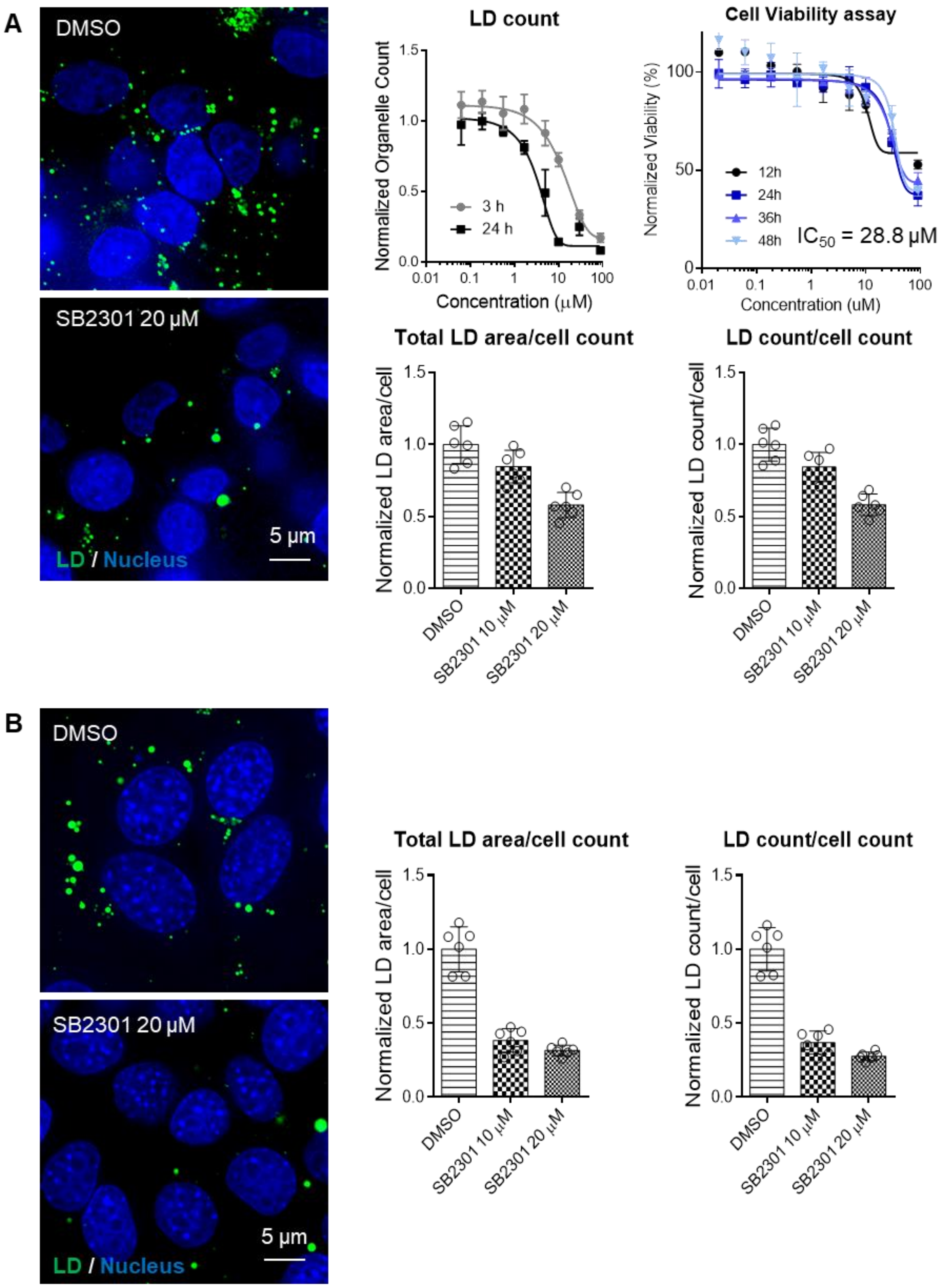

Supplementary Figure 2. Cellular LD reduction upon SB2301 treatment on hepatic cell lines. Representative LD fluorescence images and image quantification (LD count and area) results on (A) HepG2 human hepatocellular carcinoma cells and (B) AML12 mouse hepatocyte cells treated with SB2301 for 24 h. All data were shown as the mean ± SD (standard deviation).

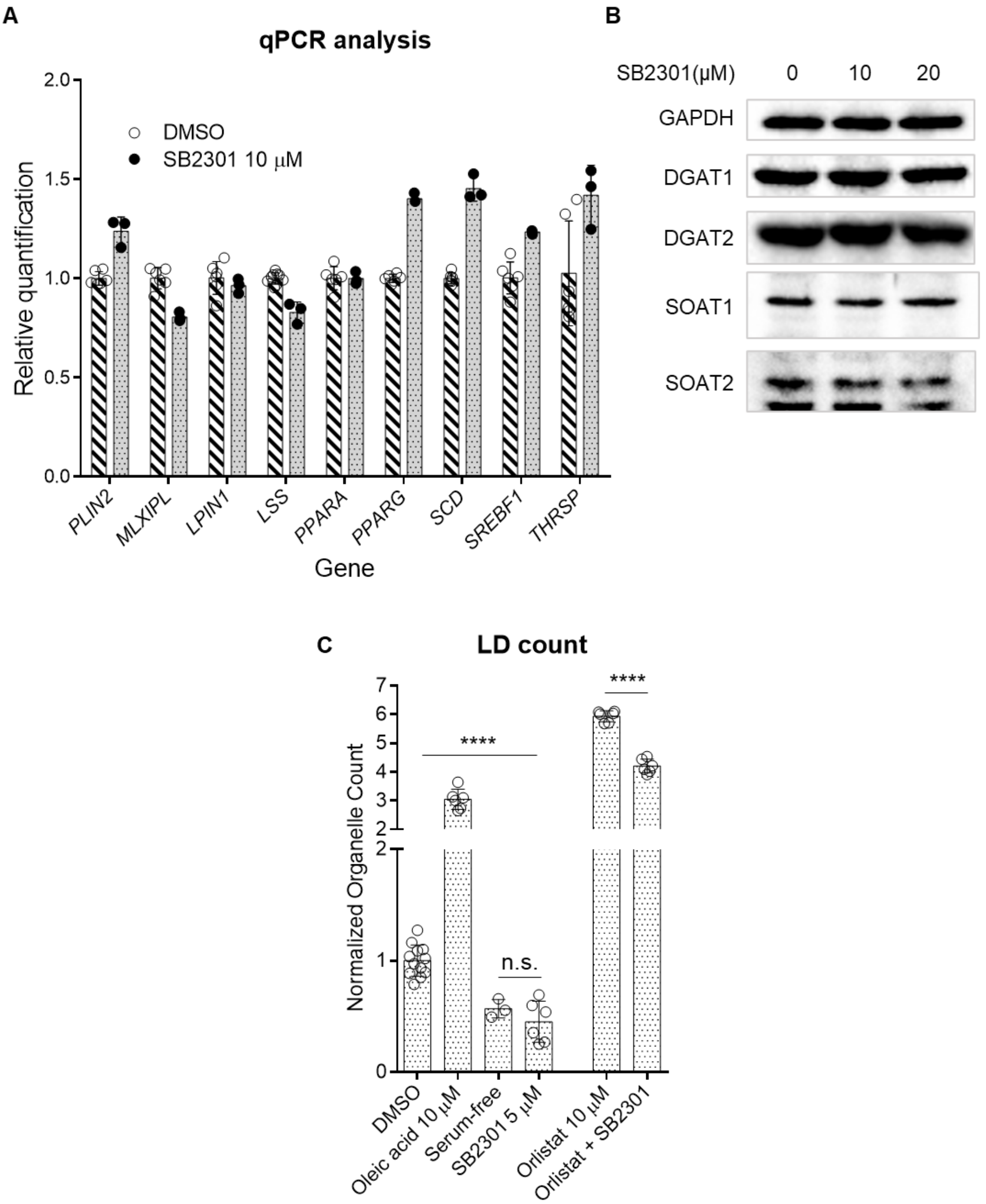

Supplementary Figure 3. SB2301 reduced cellular LDs regardless of lipid biosynthesis regulation or lipase activity. (A) The expression level of lipid biosynthesis-related genes was analyzed by qPCR. HepG2 cells were treated with 10 μM of SB2301 for 24 h. Expression levels were normalized to *GAPDH* expression level. All data were shown as the mean ± SD. (B) Western blot analysis for TG and CE synthesis regulation proteins. HepG2 cells were treated with SB2301 for 18 h. (C) LD count was analyzed after co-treatment with SB2301 and Orlistat for 24 h on HeLa cells. All data were shown as the mean ± SD, (n=3). Data were analyzed using an unpaired *t* test. ****$P < 0.0001$.

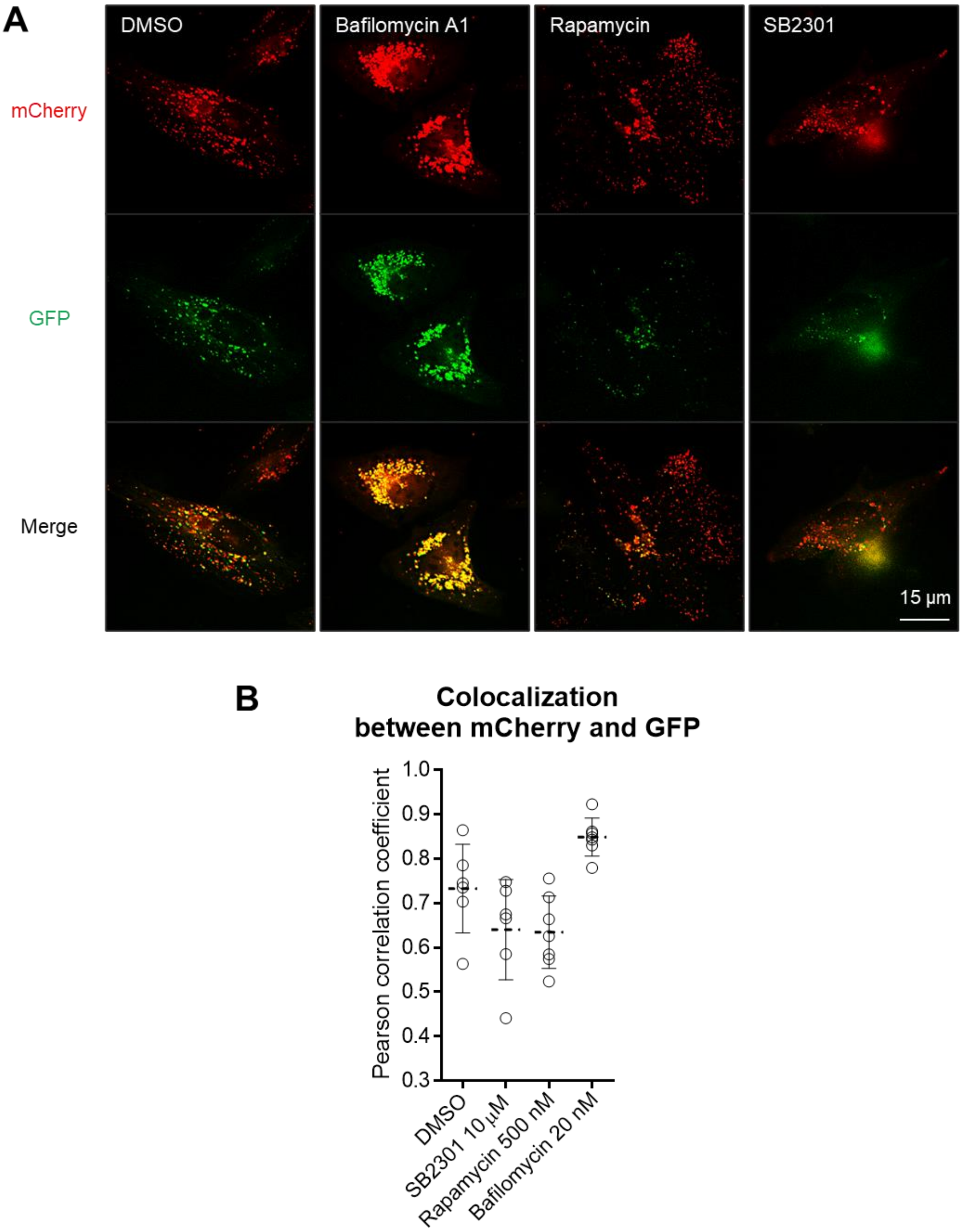

Supplementary Figure 4. SB2301 increased autophagic flux. (A) HeLa cells were transfected with mCherry-GFP-LC3 plasmid to visualize autophagic flux upon compound treatment. SB2301 (10 μM), rapamycin (500 nM), and bafilomycin A1 (20 nM) were treated for 16 h. (B) Pearson correlation coefficient measurement between mCherry and GFP signal. All data were shown in dot plots with the mean ± SD.

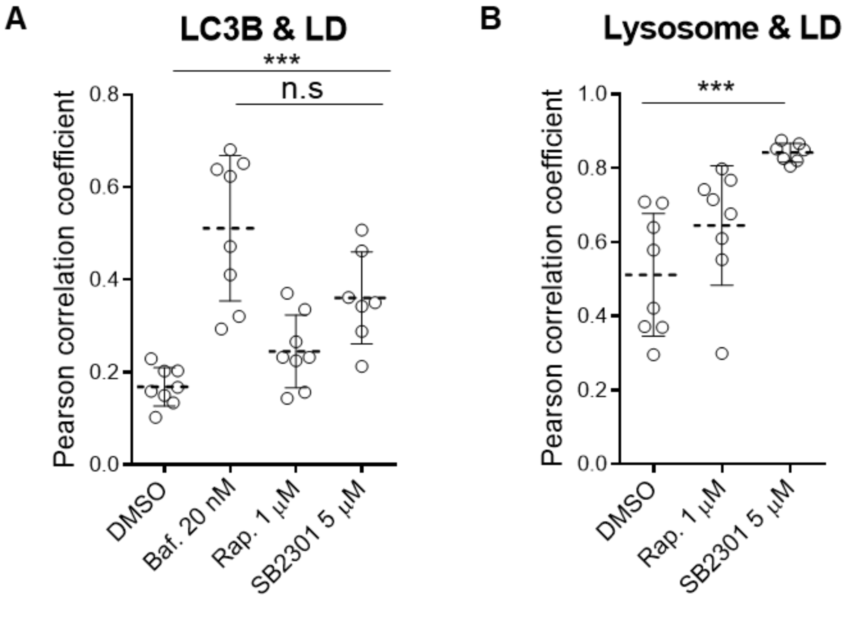

Supplementary Figure 5. Pearson correlation coefficient calculation between (A) LC3B and LD in Fig. 2E, or (B) lysosome and LD in Fig. 2F. All data were shown as the mean ± SD, (n=3). Data were analyzed using an unpaired *t* test. ***$P = 0.0002$.

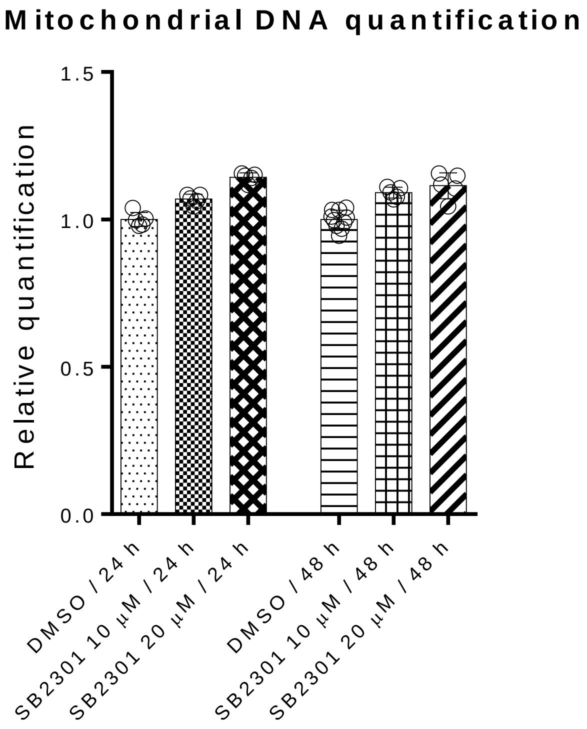

Supplementary Figure 6. Effects on mitochondrial DNA contents by SB2301. Mitochondrial DNA (mtDNA) was extracted from SB2301-treated HepG2 cells. mtDNA levels were quantified with qPCR and were normalized to *GAPDH* levels at each time point. All data were shown as the mean ± SD.

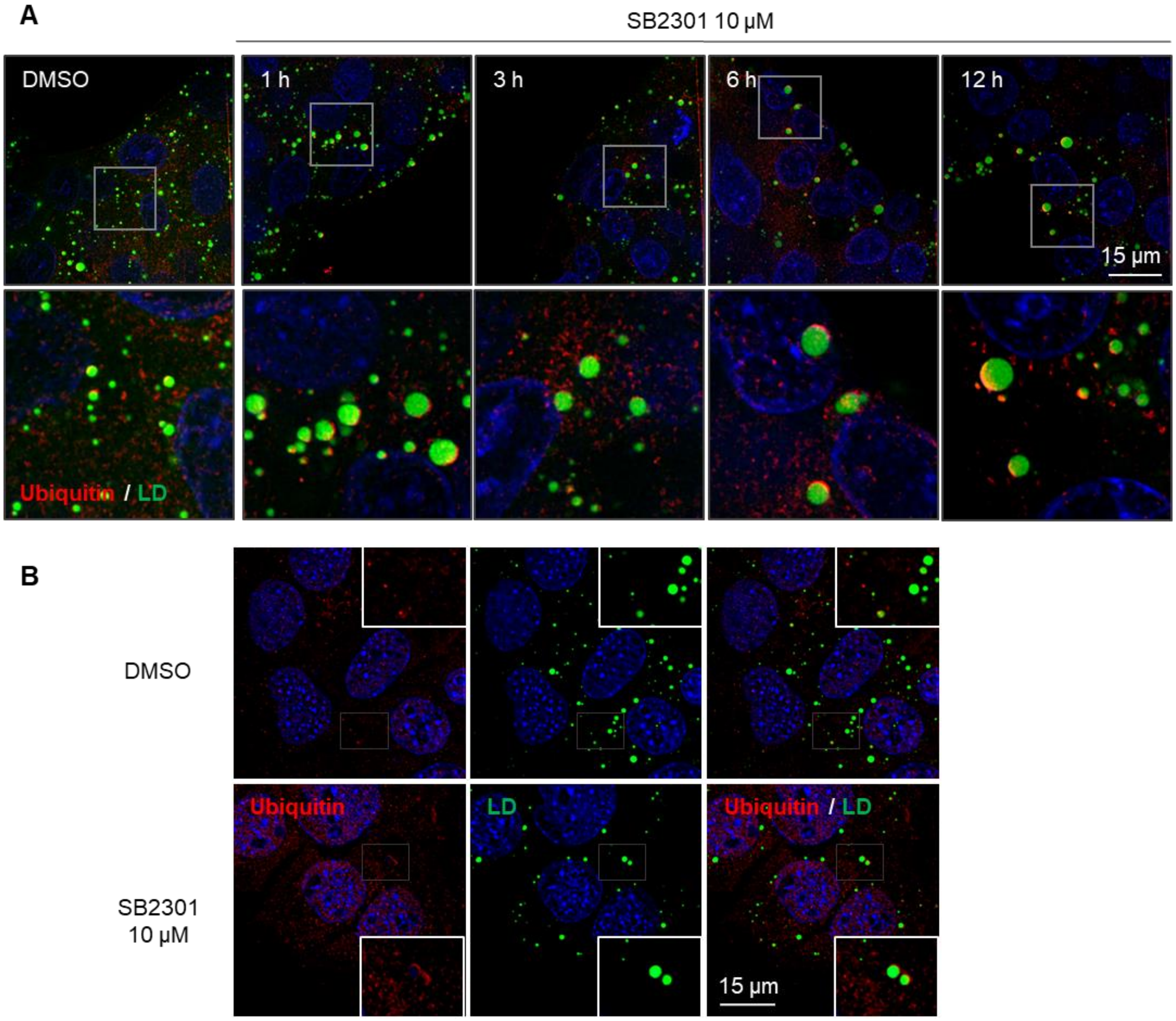

Supplementary Figure 7. SB2301 induced ubiquitination on the LD surface. Representative immunofluorescence images of ubiquitin (red) and LDs (green, BODIPY 493/503). (A) HepG2 cells were treated with 10 μM of SB2301 for indicated times and (B) AML12 cells were treated with 10 μM of SB2301 for 12 h. Cells were fixed followed by immunostaining of anti-ubiquitin antibody.

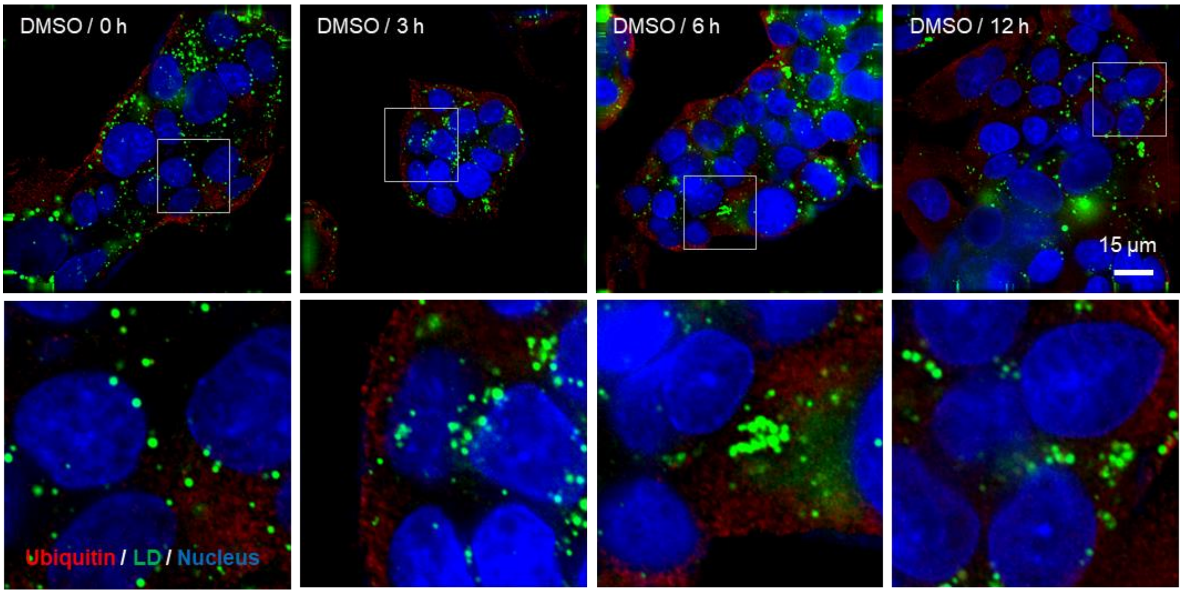

Supplementary Figure 8. Representative immunofluorescence images of ubiquitin (red), LD (green, BODIPY 493/503), and nucleus (blue, Hoechst). HepG2 cells were treated with DMSO for 0, 3, 6, or 12 h.

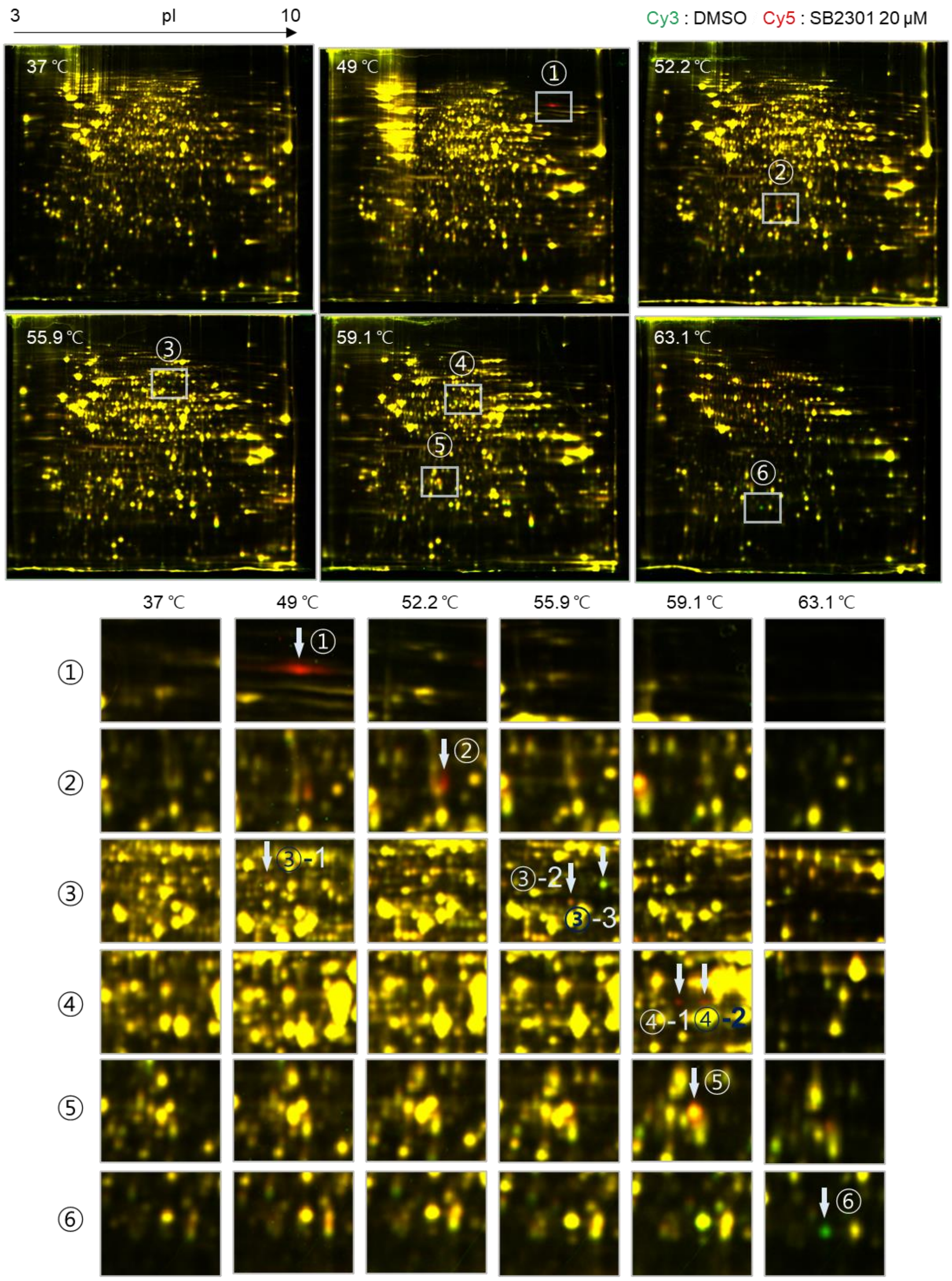

Supplementary Figure 9. TS-FITGE experiment results. Full-range images of TS-FITGE 2D gels shown in Fig. 3A. Overlaid images of the Cy3 channel (green, DMSO-treated proteome) and Cy5 channel (red, SB2301-treated proteome) at the designated temperature. Reproducible spots are marked with numbers (from 1 to 6). The proteins from those spots were identified with LC/MS/MS and listed in Supplementary Table 2.

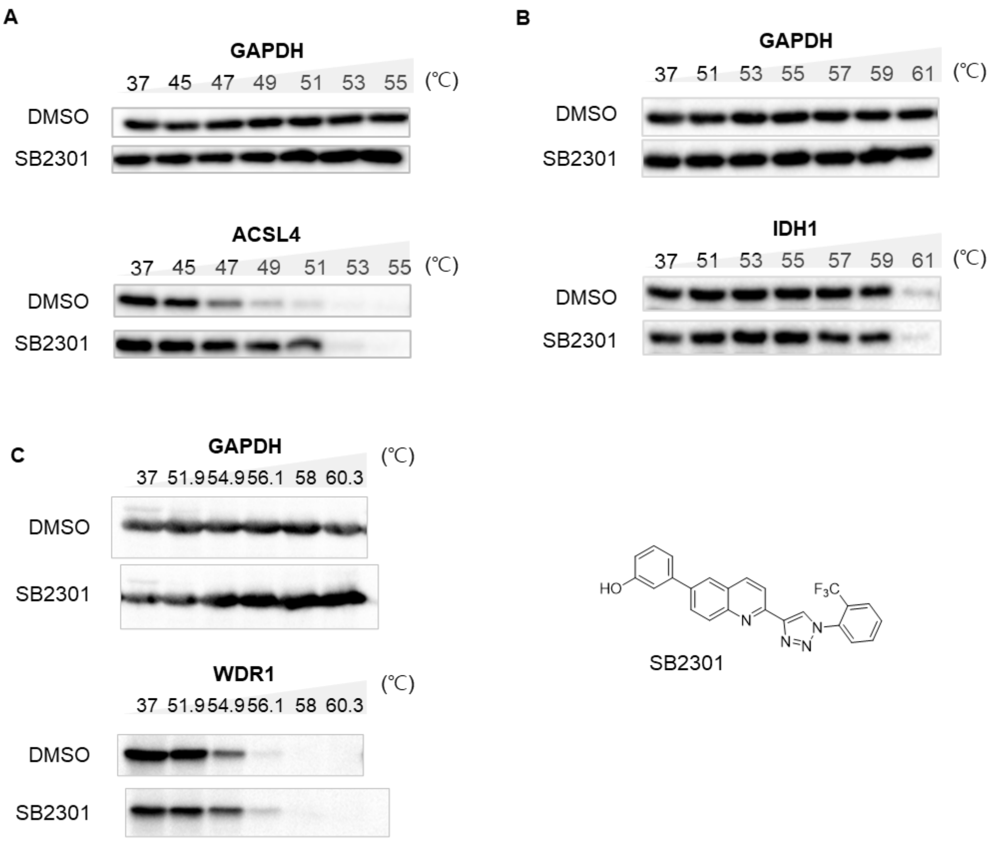

Supplementary Figure 10. Immunoblot results of cellular thermal shift assay (CETSA). HepG2 cells were treated with 20 μM of SB2301 for 1 h. The specific bindings of SB2301 towards (A) ACSL4, (B) IDH1, and (C) WDR1 were assessed by monitoring their thermal stability shifts in the western blot.

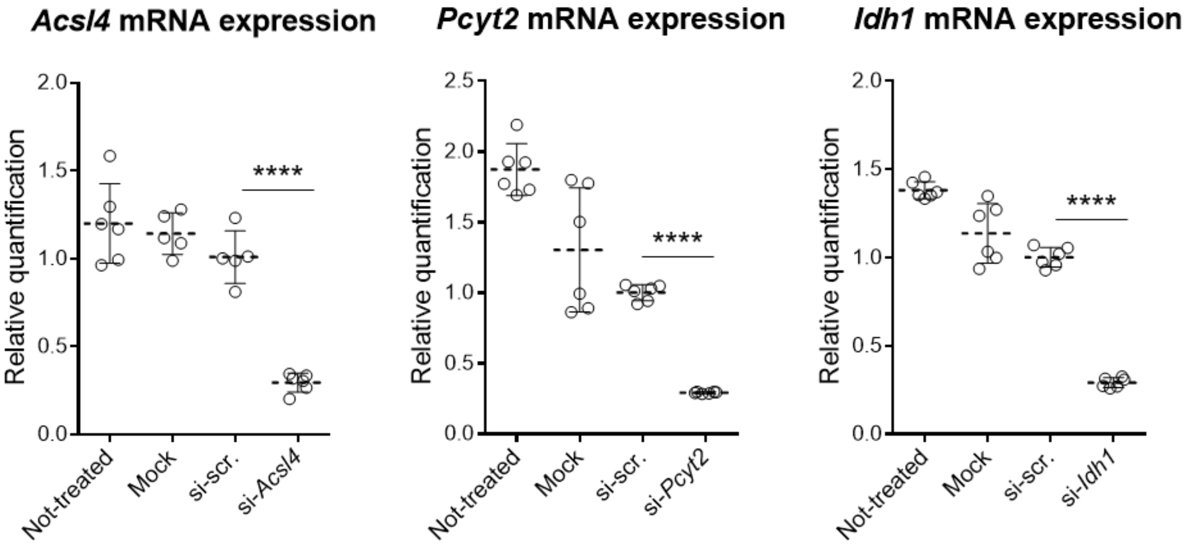

Supplementary Figure 11. Gene knock-down efficiency confirmation by RT-PCR. HepG2 cells were treated with 20 µM of each si-RNA for 48 h. Expression levels were normalized to *GAPDH* gene expression level. All data were shown as the mean ± SD, (n=3). Data were analyzed using an unpaired *t* test. ****$P < 0.0001$.

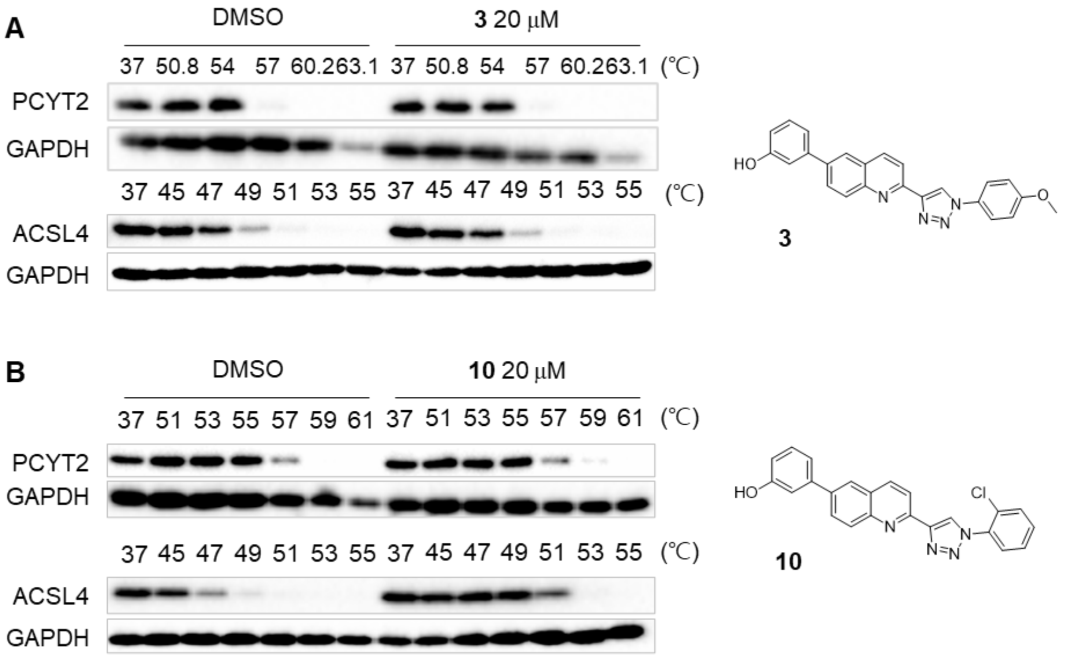

Supplementary Figure 12. Immunoblot results of CETSA. HepG2 cells were treated with 20 μM of **3** (A) or **10** (B) for 1 h. (A) The specific bindings towards PCYT2 and ACSL4 were assessed by monitoring their thermal stability shift in western blot.

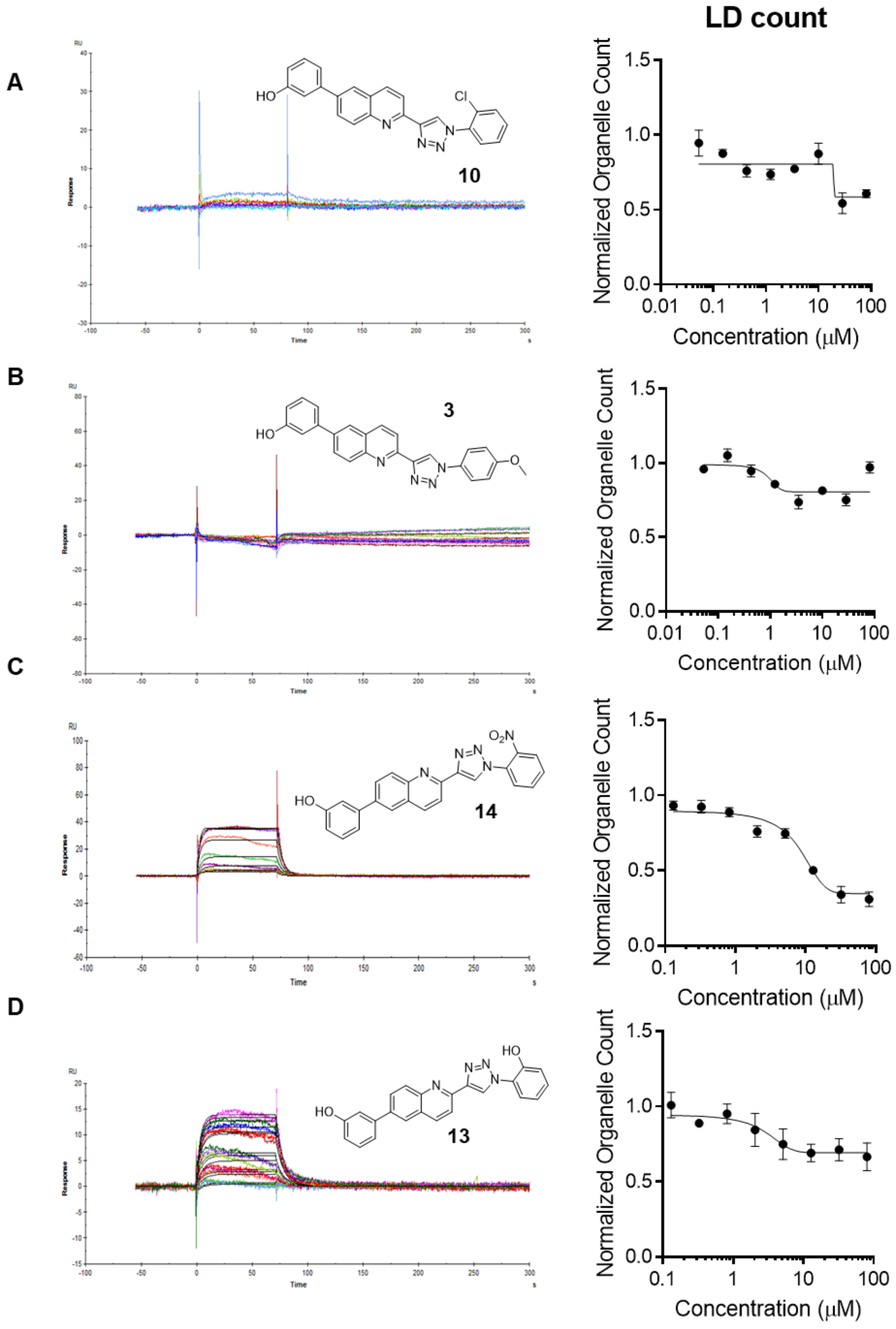

Supplementary Figure 13. Representative sensorgrams of surface plasmon resonance (SPR) analysis toward human PCYT2 (left) and dose-dependent LD-reducing activity (right) of SB2301 derivatives, **10** (A), **3** (B), **14** (C), and **13** (D). The compounds without LD-reducing activity (**10** and **3**) did not show any specific binding to PCYT2. **14**, having moderate LD-reducing activity but excluded owing to cytotoxicity, showed similar binding activity to SB2301, and **13**, having weak LD-reducing activity showed weak binding in terms of the SPR response efficacy.

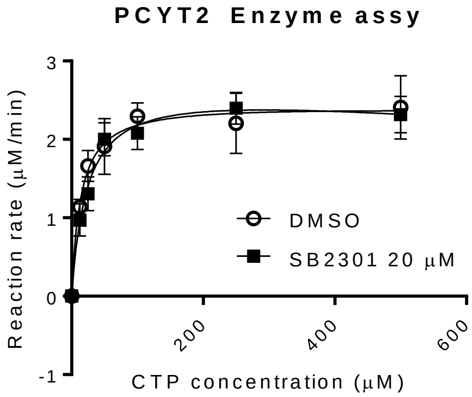

Supplementary Figure 14. PCYT2 functional assay result. *In vitro* functional activity of PCYT2 (0.1 μg) was measured in the presence of SB2301. The reaction rate was measured at 650 μM phosphoethanolamine with varying concentrations of CTP (12.5–500 μM). All data were shown as the mean ± SD.

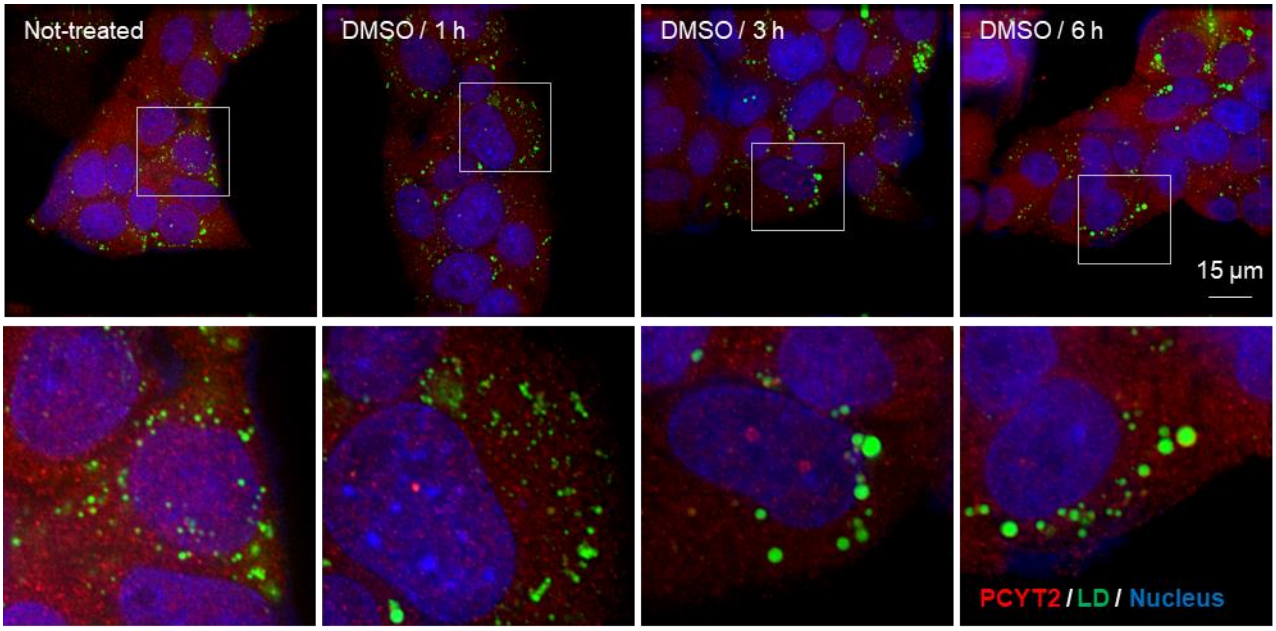

Supplementary Figure 15. Representative immunofluorescence images of PCYT2 (red), LD (green, BODIPY 493/503), and nucleus (blue, Hoechst). DMSO was treated on HepG2 cells with indicated times. Fixed cells were applied to immunostaining of PCYT2.

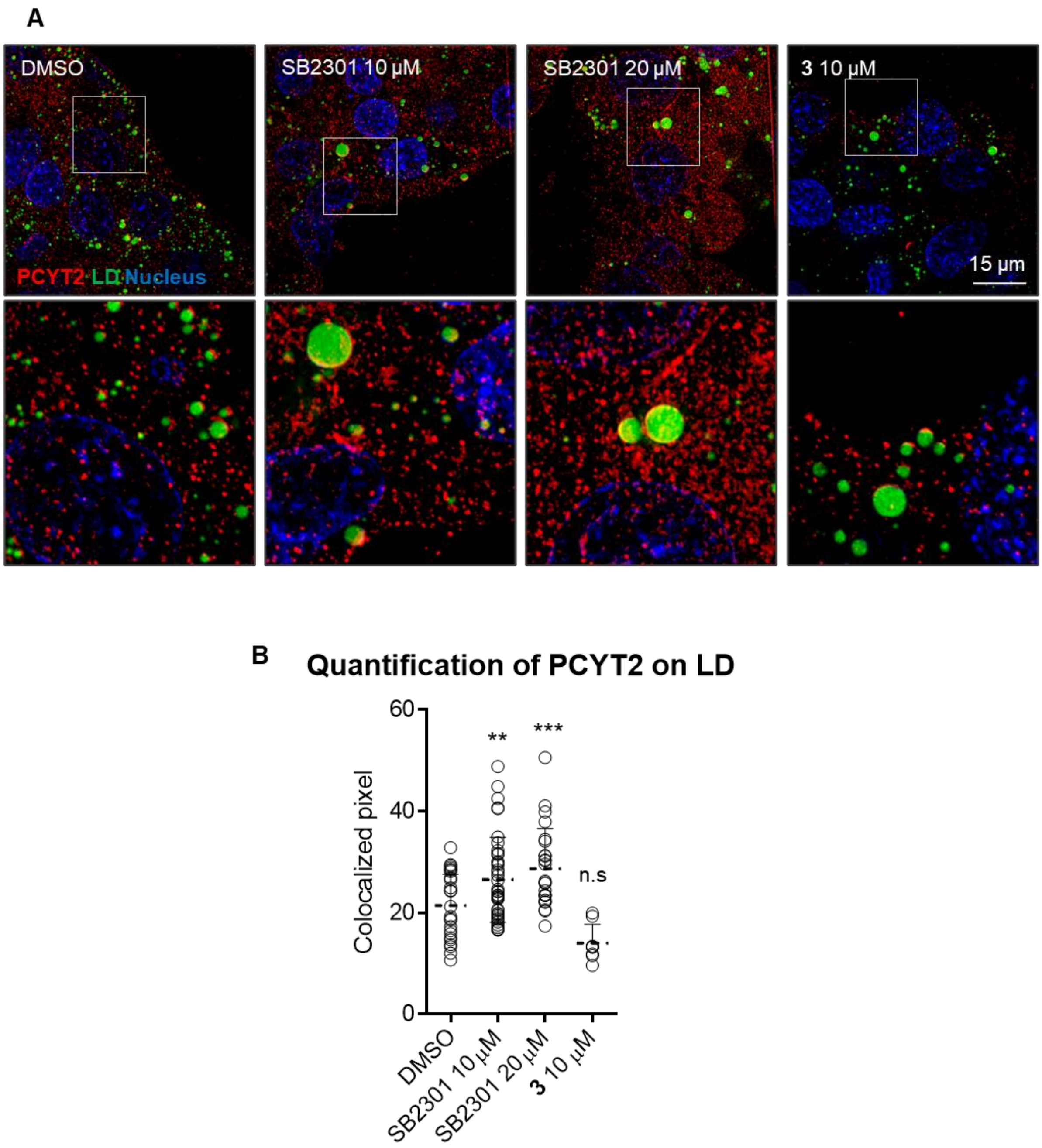

Supplementary Figure 16. PCYT2 translocation to LD surface upon SB2301 treatment. (A) Representative immunofluorescence images of PCYT2 (red), LDs (green, BODIPY 493/503) and nucleus (blue). HepG2 cells were treated with 10, 20 μM of SB2301 or 10 μM of **3** (negative compound) for 6 h followed by cell fixation. PCYT2 was labeled with anti-PCYT2 antibody and LDs were stained with BODIPY 493/503. (B) Quantification of PCYT2 on the LD. Images were selected randomly from biological triplicates. The superimposed area between LD and PCYT2 on each experimental condition was analyzed with ImageJ software. All data were shown in dot plots with the mean ± SD. Data were analyzed using an unpaired t test. $^{**}P = 0.0038$, $^{***}P = 0.0002$.

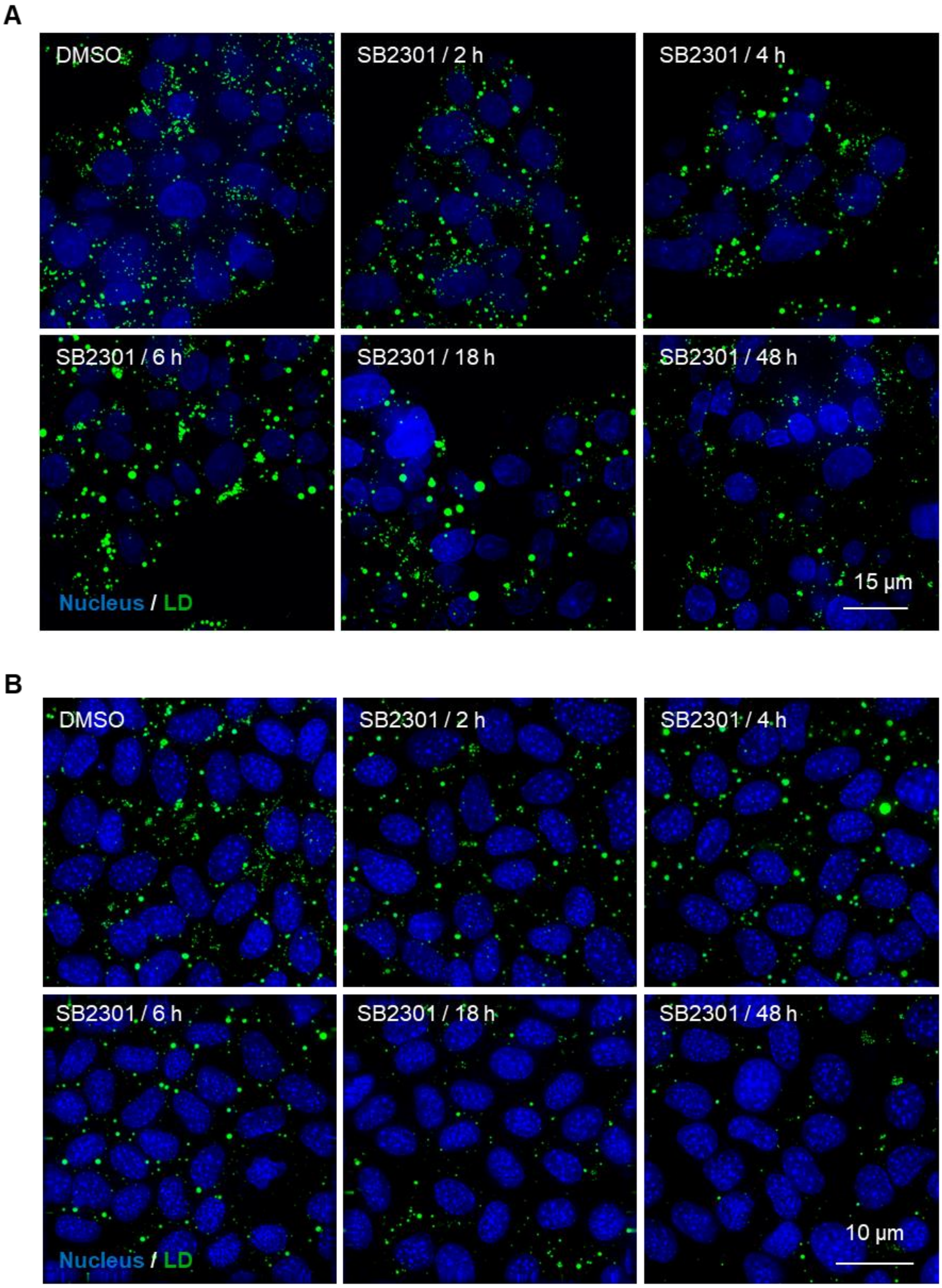

Supplementary Figure 17. Dynamic changes in LD size upon SB2301 treatment. Representative immunofluorescence images on (A) HepG2 cells and (B) AML12 cells. Cells were treated with 20 µM of SB2301 for the indicated times. Cells were fixed, and LDs were stained with BODIPY 493/503.

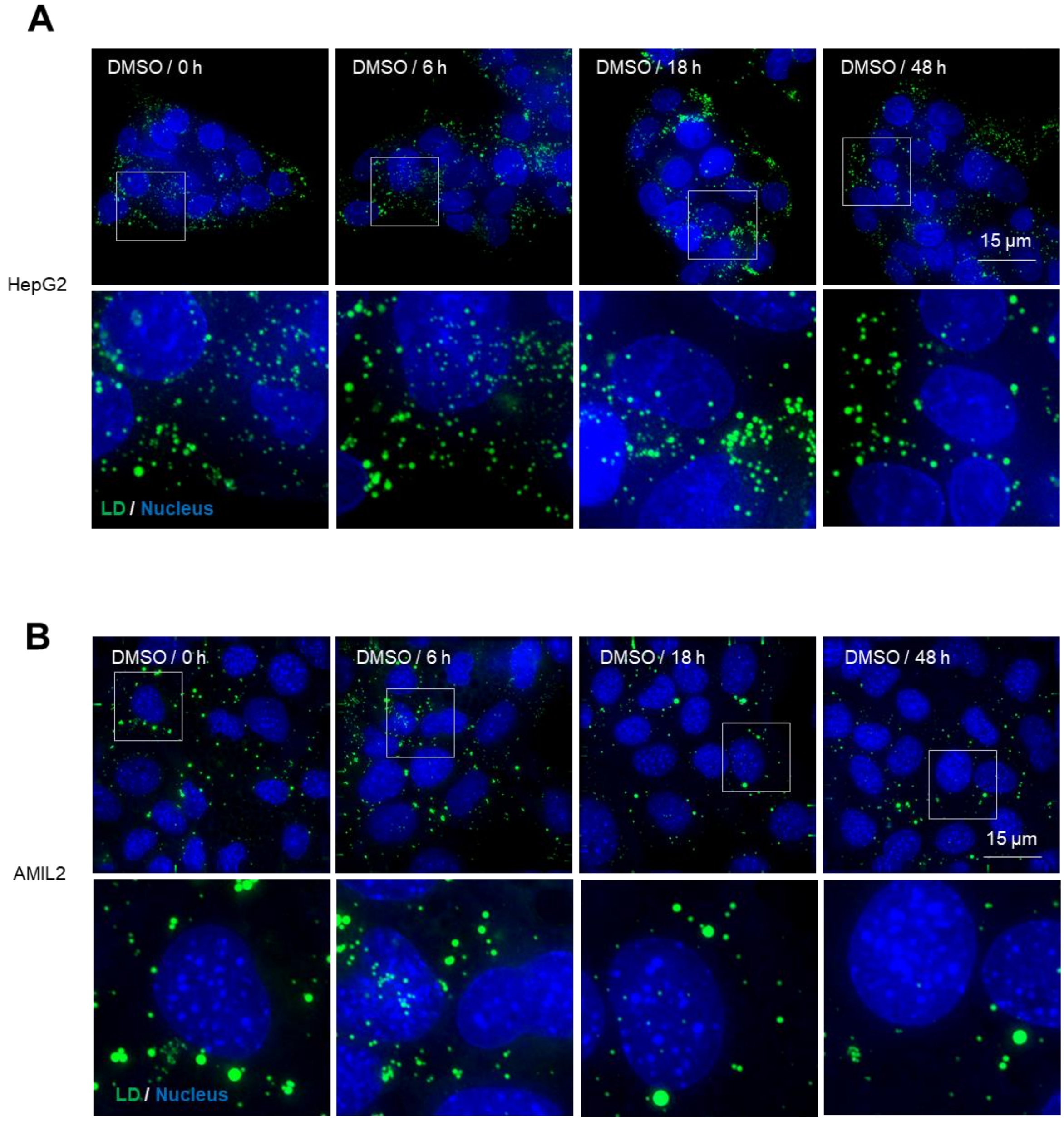

Supplementary Figure 18. Time-dependent monitoring of LD morphology upon DMSO treatment. Representative immunofluorescence images on (A) HepG2 cells and (B) AML12 cells. Cells were treated with DMSO for the indicated times. Cells were fixed and LDs were stained with BODIPY 493/503.

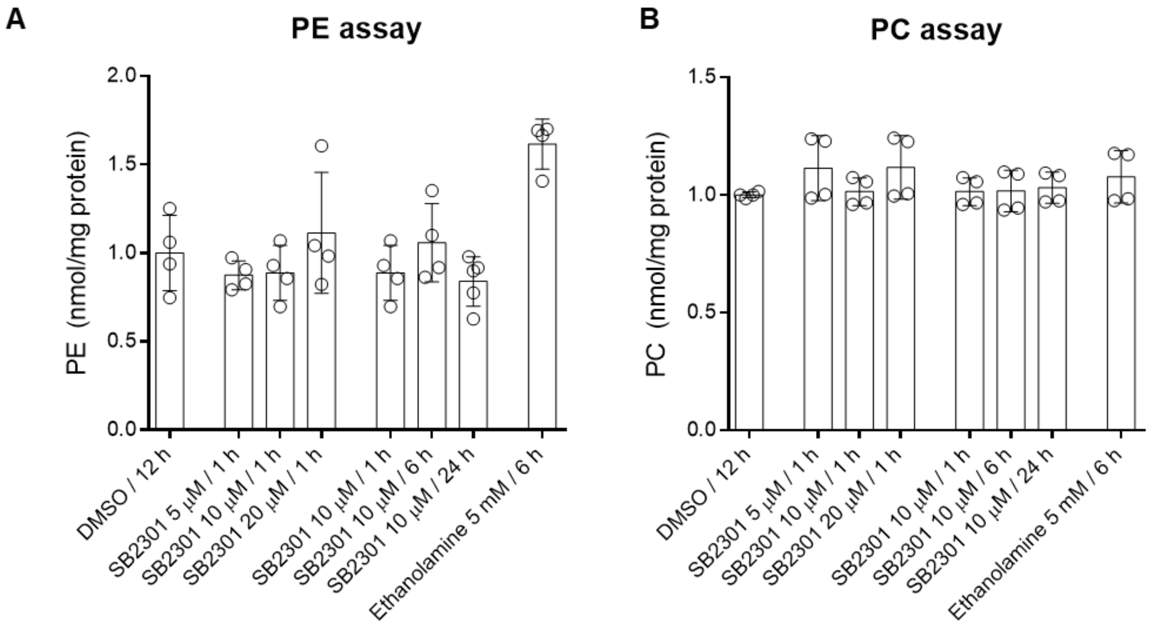

Supplementary Figure 19. PE and PC quantification assay. Phospholipids were extracted from compound-treated HepG2 cells. Total PE and PC amount per protein amount of each sample was normalized to DMSO. Each compound was treated with indicated times and concentrations. All data were shown as the mean ± SD.

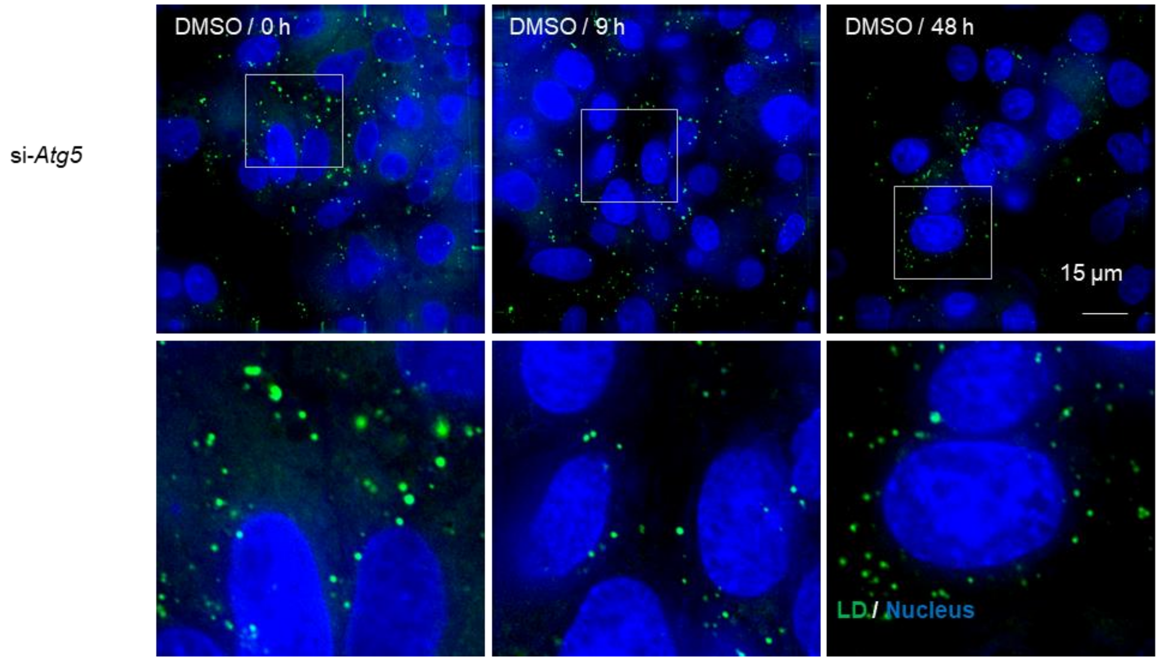

Supplementary Figure 20. Representative LD fluorescence images on *Atg5* knockdown HepG2 cells. DMSO was treated on *Atg5*-depleted cells for 0, 9, or 48 h.

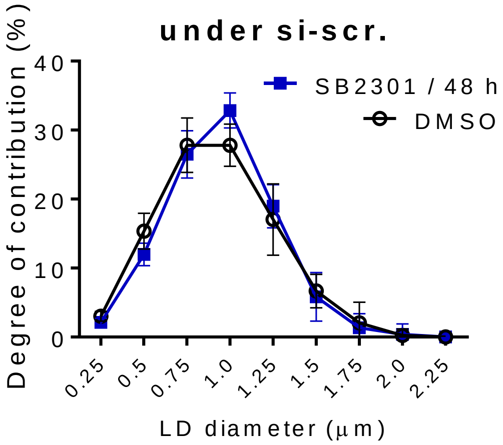

Supplementary Figure 21. HepG2 was treated with 20 μM of scrambled RNA for 48 h, and then 20 μM of SB2301 was treated for 48 h. The degree of contribution of LDs was analyzed. All data were shown as the mean ± SD.

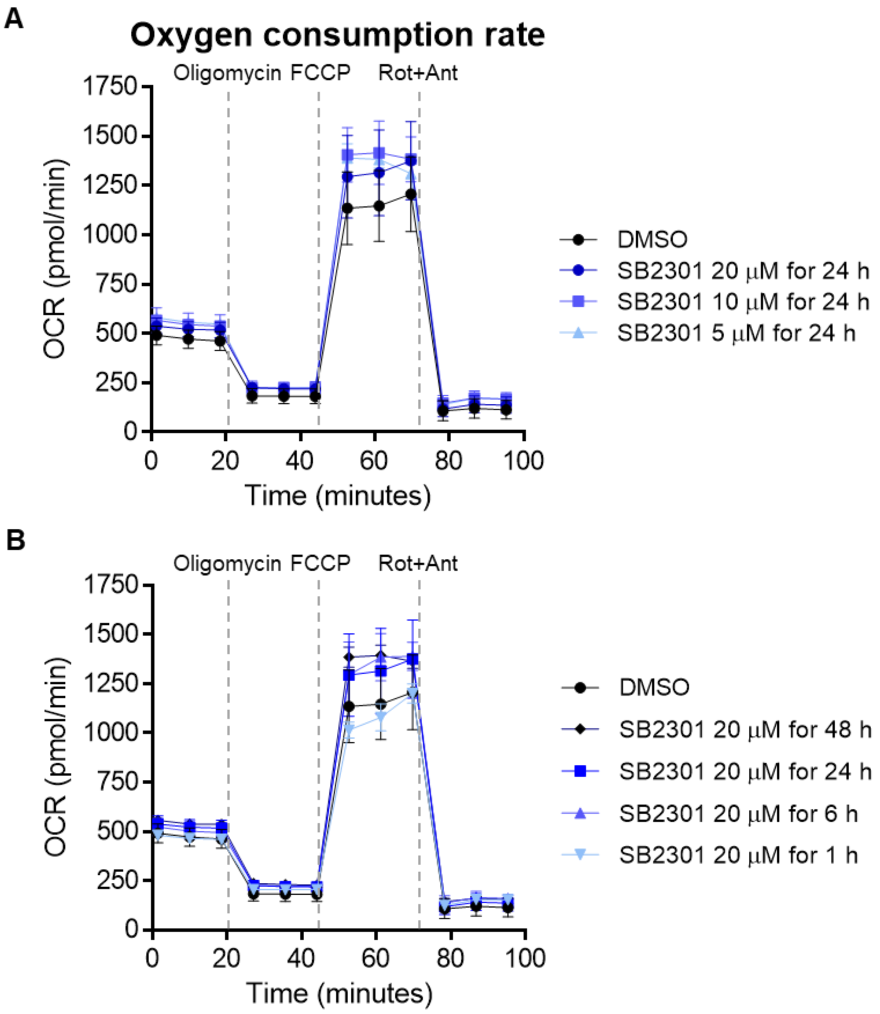

Supplementary Figure 22. Oxygen consumption rate (OCR) measurement. AML12 cells were treated with (A) 5, 10, or 20 µM of SB2301 for 24 h or (B) 20 µM of SB2301 for 1, 6, 24, or 48 h. All data were shown as the mean ± SD. Rot+Ant; rotenone + antimycin A.

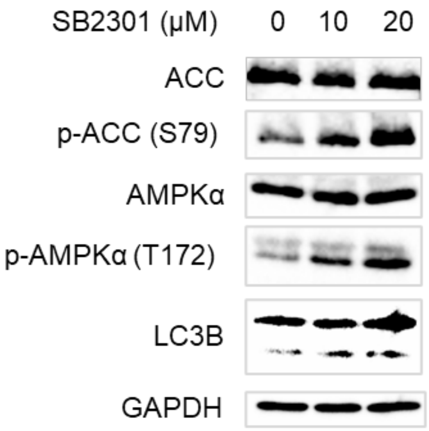

Supplementary Figure 23. Immunoblot results of mitochondrial beta-oxidation. AML12 cells were treated with SB2301 for 24 h.

Supplementary Figure 24. Uncropped full blot images

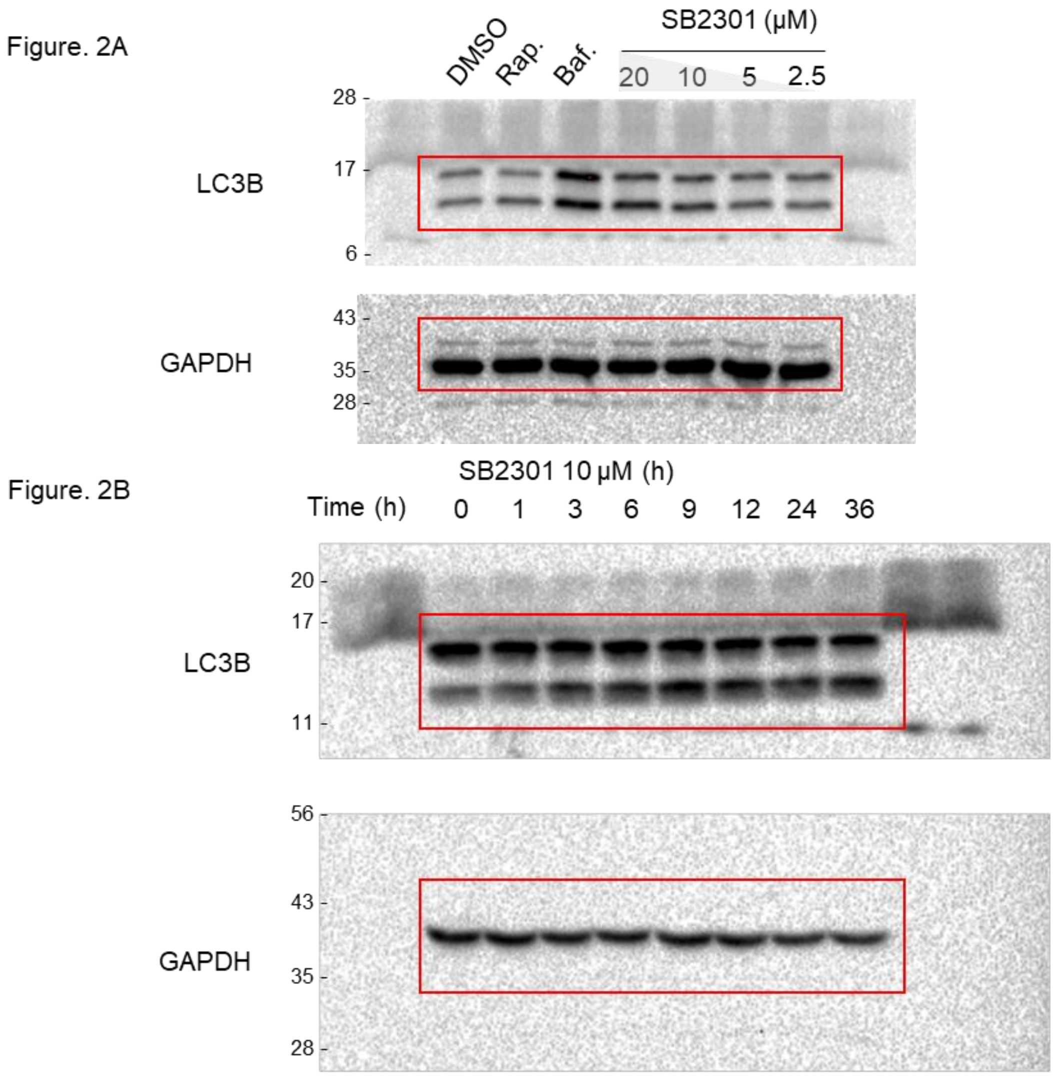

Figure. 2C

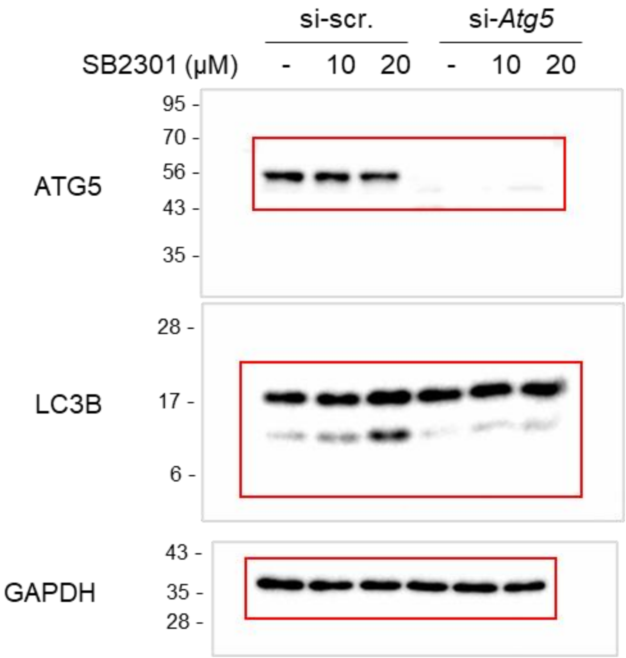

Figure. 2D

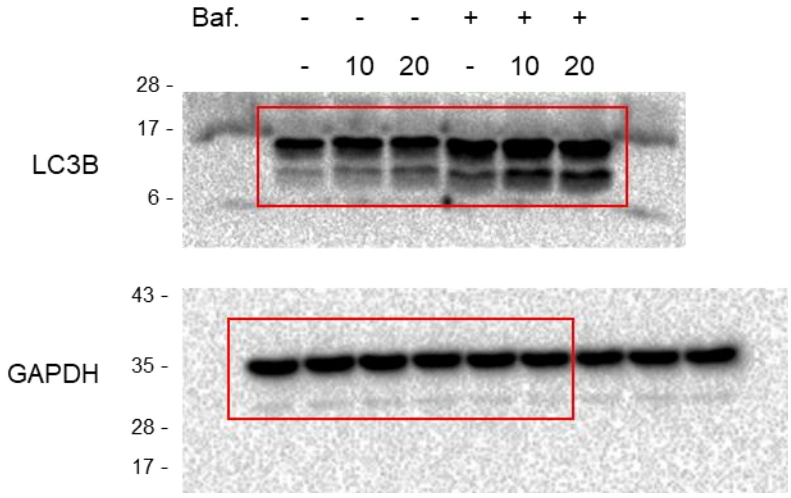

Figure. 3B

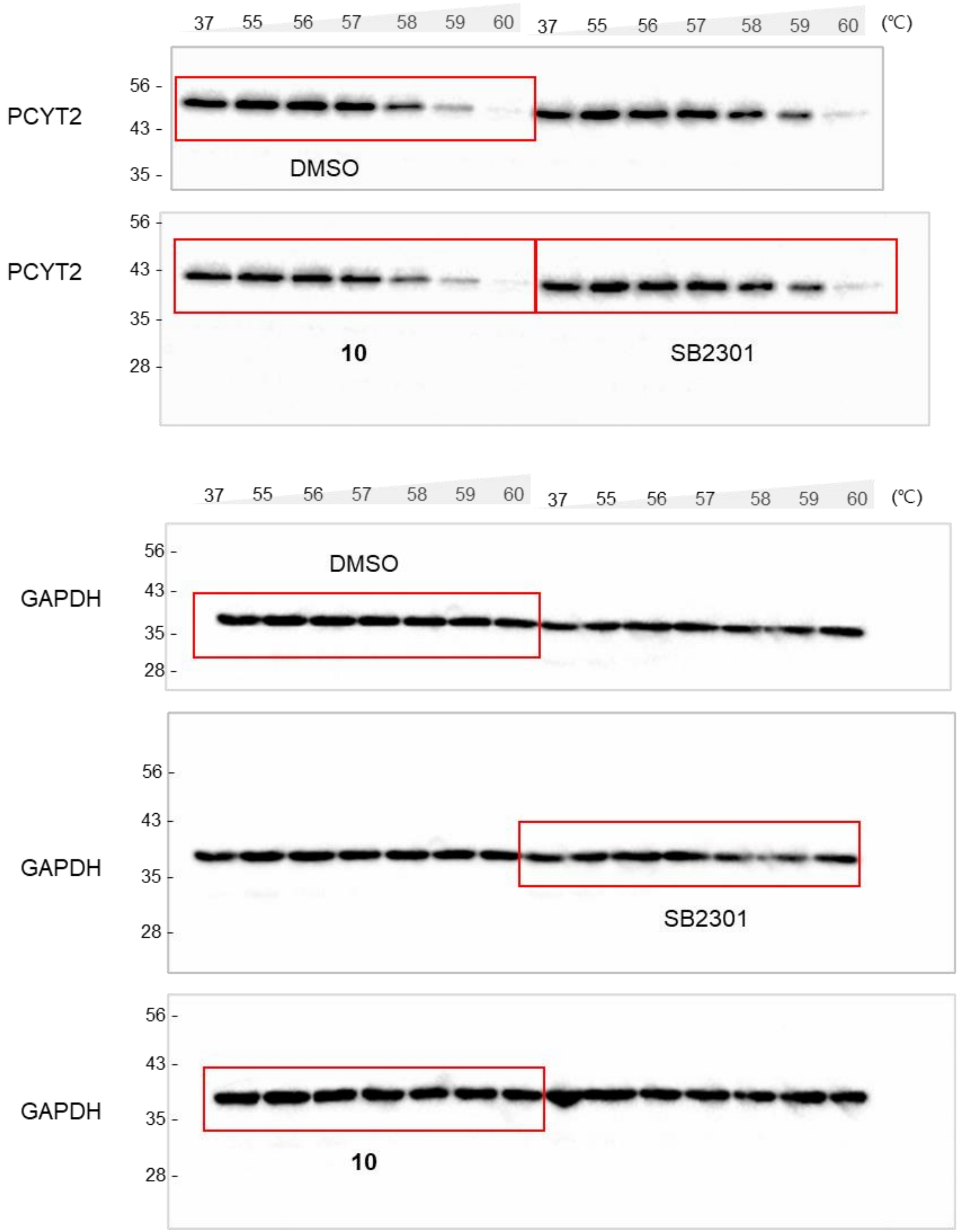

37 55 56 57 58 59 60 37 55 56 57 58 59 60 (℃)
PCYT2
56 -
43 -
35 -
DMSO
PCYT2
56 -
43 -
35 -
28 -
10
SB2301
37 55 56 57 58 59 60 37 55 56 57 58 59 60 (℃)
GAPDH
56 -
43 -
35 -
28 -
DMSO
GAPDH
56 -
43 -
35 -
28 -
SB2301
GAPDH
56 -
43 -
35 -
28 -
10

Supplementary Figure. 3B

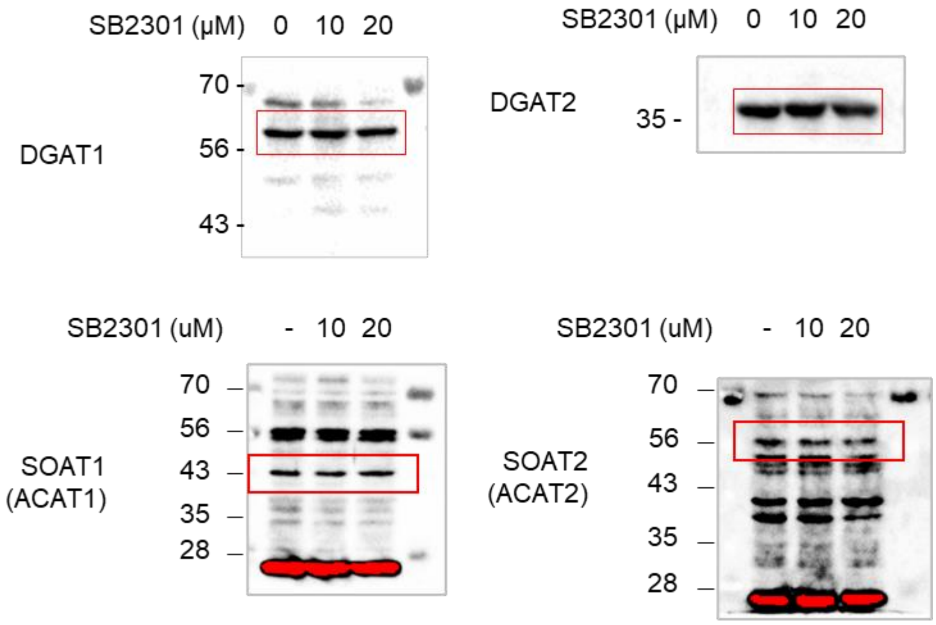

Supplementary Figure. 10A

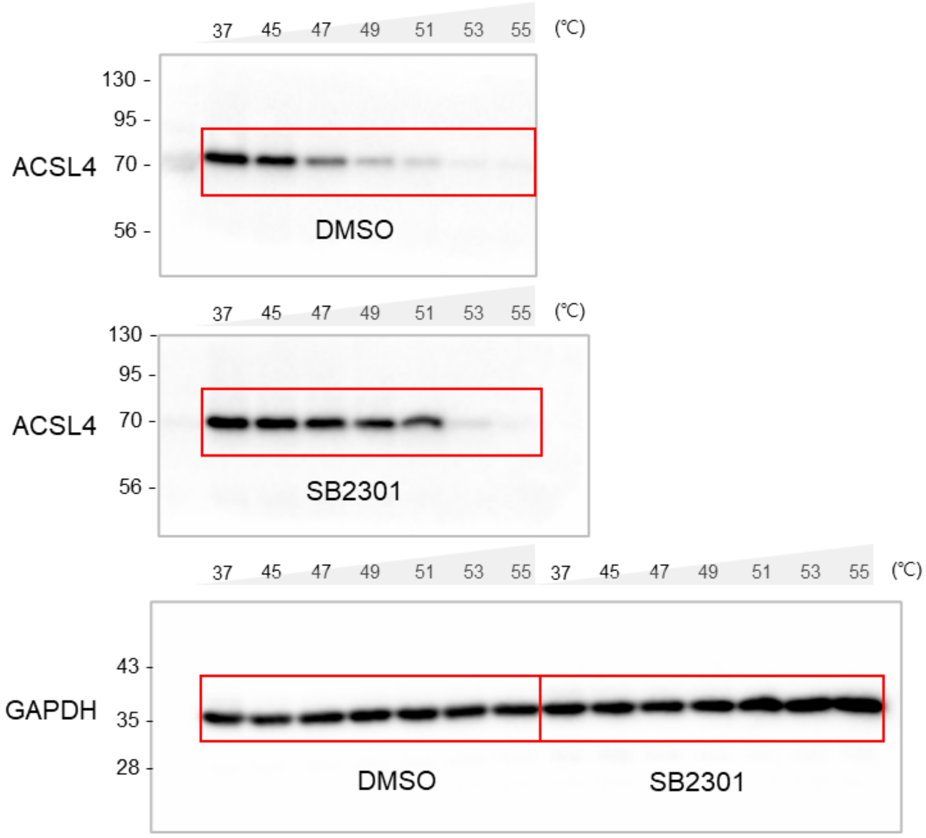

Supplementary Figure. 10B

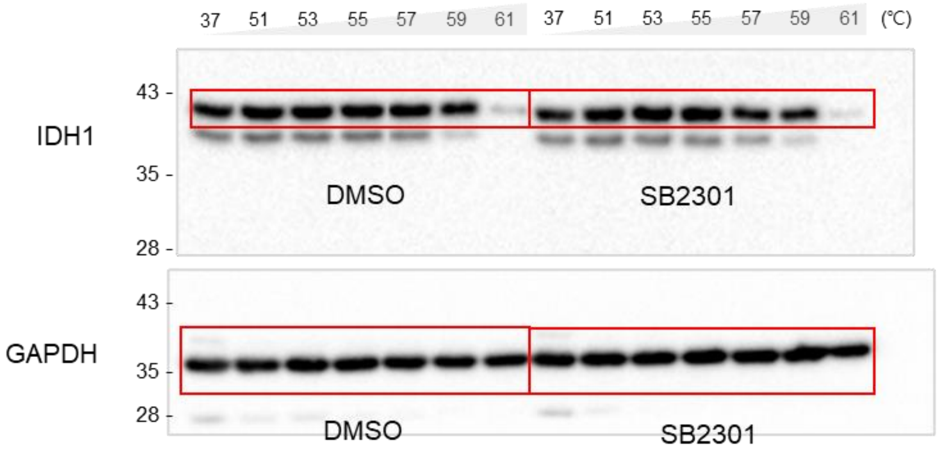

Supplementary Figure. 10C

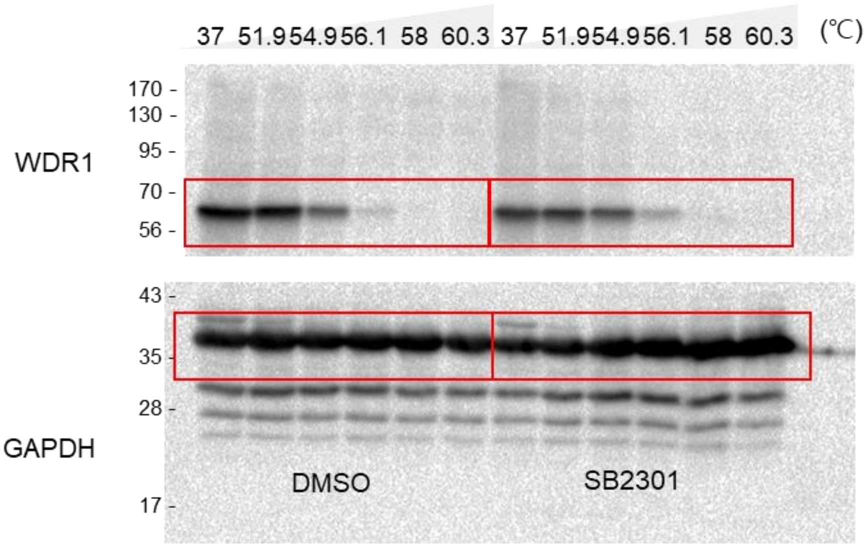

Supplementary Figure. 12

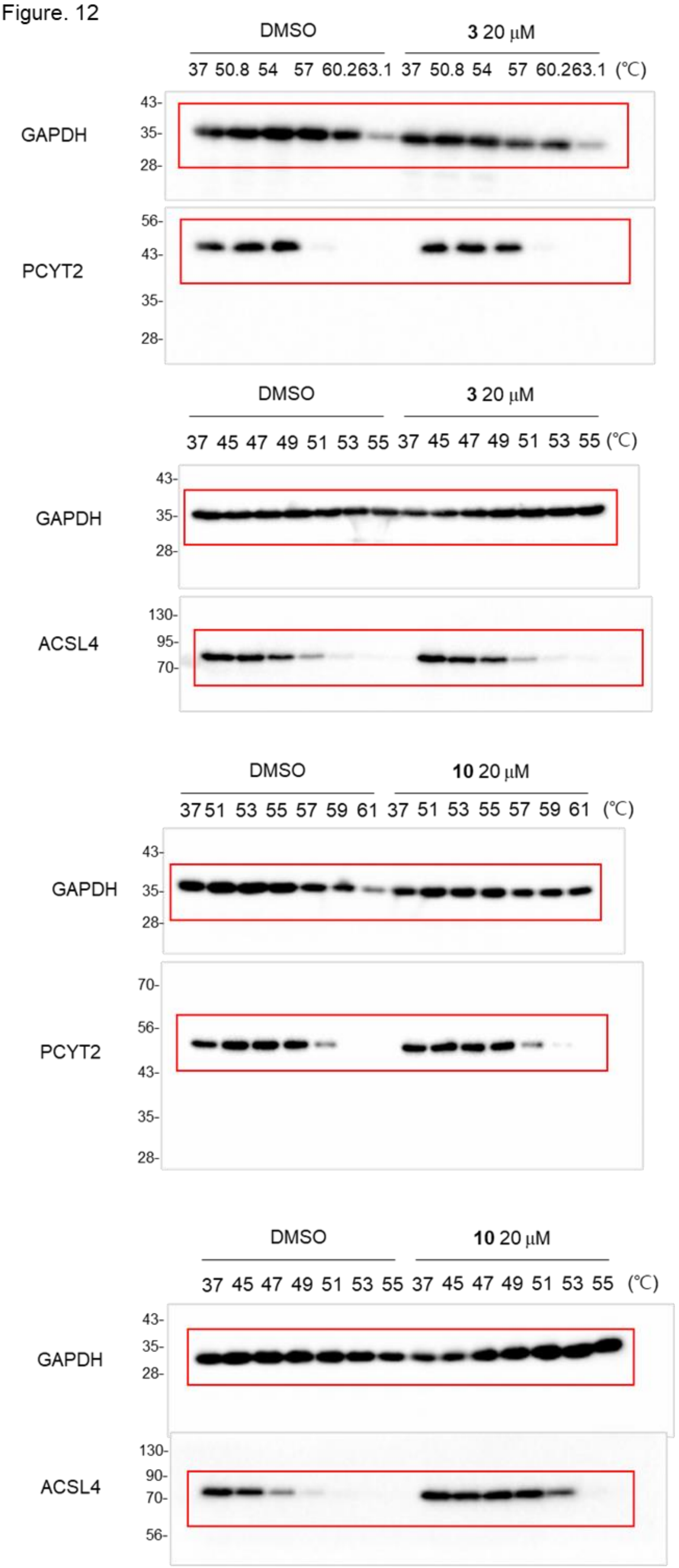

Supplementary Figure. 23

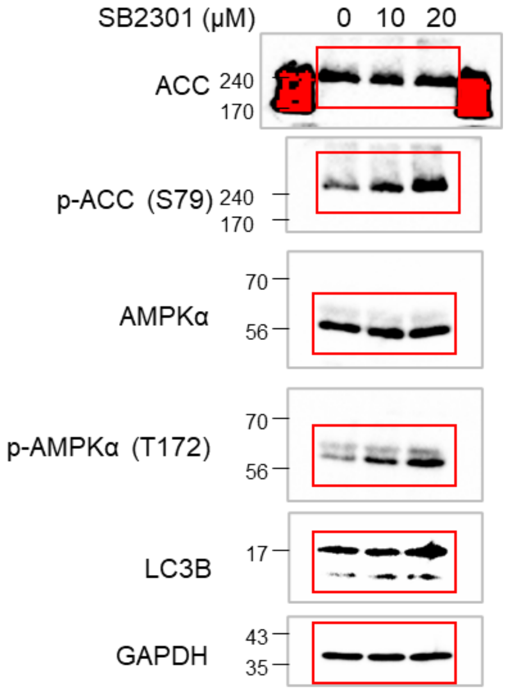

II. Supplementary Notes

Supplementary Scheme 1. Synthesis of (3-(quinolin-6-yl)phenol 1,4-disubstituted 1,2,3-triazole libraries

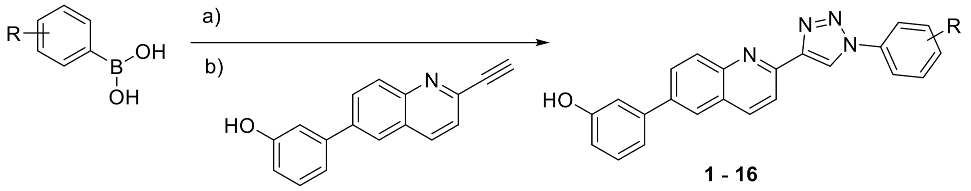

**1**: R = 2-OCH_3	**4**: R = 2-CF_3	**7**[a]: R = 2-CN	**10**: R = 2-Cl	**13**: R = 2-OH
**2**: R = 3-OCH_3	**5**: R = 3-CF_3	**8**[a]: R = 2-CN	**11**: R = 3-F	**14**: R = 2-NO_2
**3**: R = 4-OCH_3	**6**: R = 4-CF_3	**9**[a]: R = 3-CN	**12** R = 4-Br	**15**: R = 2-CH_3
				**16**: R = H

Reagents and conditions: a) NaN_3 (1.1 equiv.), $CuSO_4$ (0.1 equiv.), MeOH, r.t., air; b) 3-(2-ethynylquinolin-6-yl)phenol (1.0 equiv.), sodium ascorbate (0.4 equiv.), MeOH:H_2O (1:1), 50 ºC

Supplementary Scheme 2. Phosphatidylethanolamine (PE) synthesis pathway

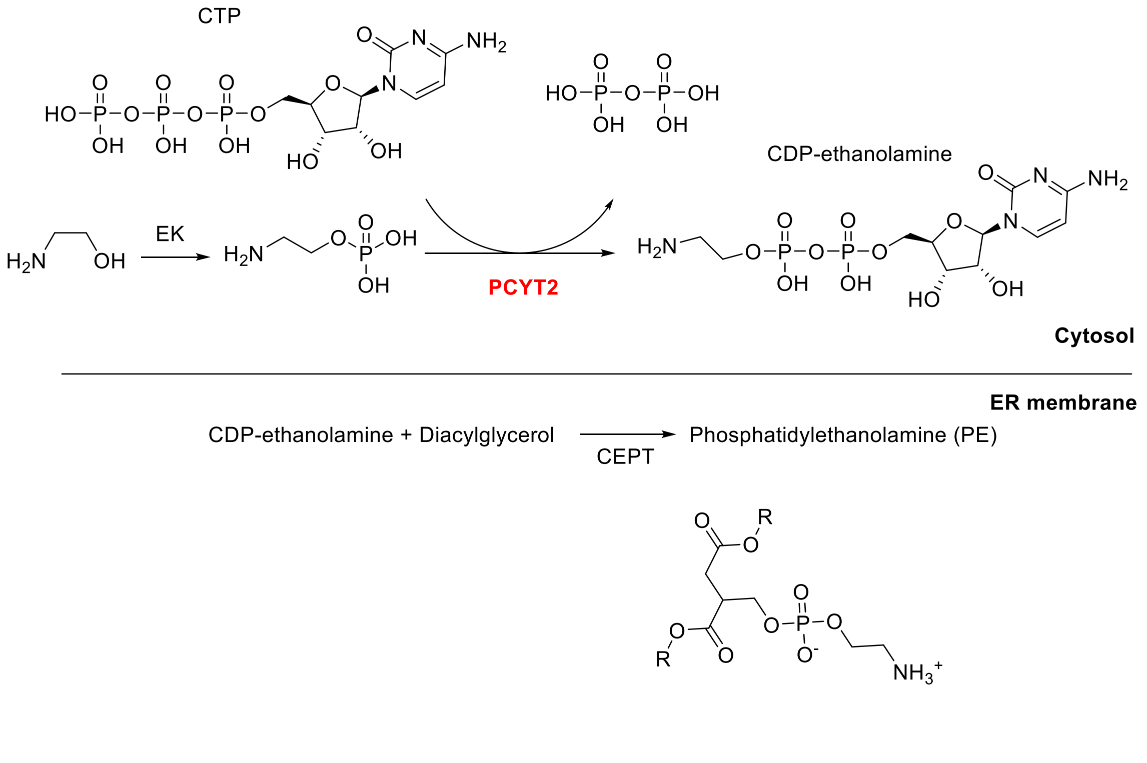

Supplementary Note 1. General Information of Synthetic Protocols

All commercially available reagents and solvents were used without further purification unless noted otherwise. All the solvents were purchased from commercial venders. ^{1}H and ^{13}C NMR spectra were obtained using Agilent 400-NMR [Agilent Technologies] and Varian Inova-500 [Varian Associates]. Chemical shifts were reported in ppm from tetramethylsilane (TMS) as internal standard or the residual solvent peak ($CDCl_3$; ^{1}H: δ = 7.26 ppm; ^{13}C: δ = 77.16 ppm / $(CD_3)_2OD$; ^{1}H: δ = 2.05 ppm; ^{13}C: δ = 49.00 ppm). Multiplicity was indicated as follows: s (singlet), d (doublet), t (triplet), q (quartet), m (multiplet), dd (doublet of doublet), dt (doublet of triplet), td (triplet of doublet), brs (broad singlet), and so on. Coupling constants are reported in hertz. Mass spectrometric analysis was conducted by LCMS-2020 [Shimadzu]. The conversion of starting materials was monitored by thin–layer chromatography (TLC) using pre-coated glass-backed plates (silica gel 60; F_{254} = 0.25 mm), and the reaction components were visualized by observation under UV light (254 and 365 nm) or by treatment of TLC plates with visualizing agents such as $KMnO_4$, phosphomolybdic acid, and ninhydrin followed by heating. Products were purified by flash column chromatography on silica gel (230-400 mesh) using a mixture of EtOAc/hexane or MeOH/CH_2Cl_2 as eluents.

Supplementary Note 2. Synthetic Procedures and Characterization of All New Compounds

To a solution of *3-(2-ethynylquinolin-6-yl)phenol* in MeOH:H_2O (1:1), azides (1.2 equiv.), $CuSO_4$ (0.1 equiv.) and sodium ascorbate (0.4 equiv.) were added. After stirring at 50 ºC, until starting materials were consumed (the reaction completion was checked by TLC), the reaction mixture was diluted with EA and washed with deionized water and brine. The combined organic layer was dried with anhydrous Na_2SO_4(s). After removing the solvent under the reduced pressure, the residue was purified by silica-gel flash column chromatography to obtain the desired compounds (**1**–**17**).

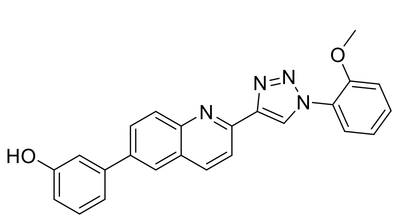

Compound 1: ^{1}H NMR (400 MHz, $(CD_3)_2SO$): δ 9.64 (s, 1H), 9.07 (s, 1H), 8.57 (d, *J* = 8.6 Hz, 1H), 8.32 (d, *J* = 8.5 Hz, 1H), 8.26 (d, *J* = 2.0 Hz, 1H), 8.07–8.12 (m, 2H), 7.74 (dd, *J* = 7.9, 1.7 Hz, 1H), 7.59 (td, *J* = 7.8, 1.4 Hz, 1H), 7.39–7.31 (m, 2H), 7.26 (d, *J* = 7.7 Hz, 1H), 7.22–7.18 (m, 2H), 6.85–6.83 (m, 1H), 3.92 (s, 3H); ^{13}C NMR (100 MHz, $(CD_3)_2SO$): δ 158.0, 151.9, 150.0, 147.2, 147.0, 140.7, 138.1, 137.6, 131.1, 130.1, 129.2, 129.1, 127.6, 126.0, 125.8, 125.6, 125.3, 120.9, 118.8, 117.8, 114.9, 113.8, 113.1, 56.2; R_f = 0.31 (EtOAc/Hexane1:3); LRMS(ESI+): Calcd for $C_{24}H_{19}N_4O_2^+$ $[M+H]^+$ 395.2, found 395.2.

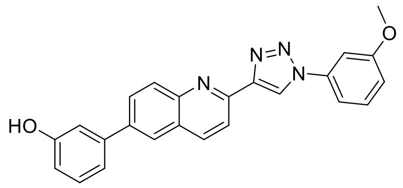

Compound 2: ^{1}H NMR (400 MHz, $(CD_3)_2SO$): δ 9.64 (s, 1H), 9.55 (s, 1H), 8.56 (s, 1H), 8.32 (d, *J* = 8.6 Hz, 1H), 8.26 (s, 1H), 8.15–8.03 (m, 2H), 7.67 (d, *J* = 7.3 Hz, 2H), 7.53 (t, *J* = 8.4 Hz, 1H), 7.33 (t, *J* = 7.8 Hz, 1H), 7.26 (d, *J* = 7.7 Hz, 1H), 7.22 (t, *J* = 2.0 Hz, 1H), 7.09 (d, *J* = 8.6 Hz, 1H), 6.85 (d, *J* = 7.9 Hz, 1H), 3.90 (s, 3H); ^{13}C NMR (100 MHz, $(CD_3)_2SO$): δ 160.9, 158.6, 150.4, 148.8, 147.6, 141.2, 138.7, 138.2, 138.1, 131.3, 130.6, 129.7, 129.5, 128.2, 125.8, 122.5, 119.2, 118.2, 115.3, 114.2, 112.6, 106.1, 55.8; R_f = 0.31 (EtOAc/Hexane1:3); LRMS(ESI+): Calcd for $C_{24}H_{19}N_4O_2^+$ $[M+H]^+$ 395.2, found 395.2.

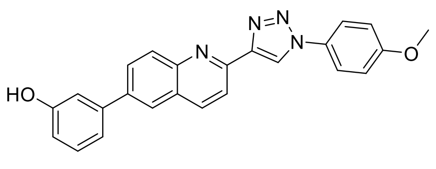

Compound 3: ^{1}H NMR (400 MHz, $(CD_3)_2CO$): δ 9.07 (s, 1H), 8.52 (d, *J* = 8.6 Hz, 1H), 8.40 (d, *J* = 8.6 Hz, 1H), 8.22 (d, *J* = 2.0 Hz, 1H), 8.12–8.05 (m, 3H), 7.99 (d, *J* = 9.0 Hz, 1H), 7.3–7.30 (m, 3H), 7.20 (d, *J* = 9.1 Hz, 2H), 6.93–6.90 (m, 1H), 3.93 (s, 3H); ^{13}C NMR (100 MHz, $(CD_3)_2SO$): δ 160.1, 158.6, 150.5, 148.7, 147.6, 141.2, 138.7, 138.1, 130.7, 130.4, 129.7, 129.5, 128.2, 125.8, 122.4, 122.3, 119.2, 118.3, 115.4, 115.3, 114.2, 55.7; R_f = 0.31 (EtOAc/Hexane1:3); LRMS(ESI+): Calcd for $C_{24}H_{19}N_4O_2^+$ $[M+H]^+$ 395.2, found 395.2.

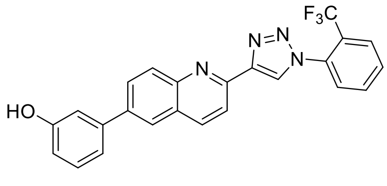

Compound 4 (SB2301): ^{1}H NMR (400 MHz, $(CD_3)_2SO$): δ 9.66 (s, 1H), 9.43 (s, 1H), 8.60 (d, *J* = 8.6 Hz, 1H), 8.35–8.25 (m, 3H), 8.13–7.97 (m, 4H), 7.9–7.85 (m, 1H), 7.37–7.18 (m, 3H), 6.89–6.81 (m, 1H); ^{13}C NMR (100 MHz, $(CD_3)_2SO$): δ 158.6, 150.2, 148.0, 147.5, 141.2, 138.8, 138.3, 134.6, 131.9, 130.6, 129.9, 129.7, 129.6, 128.2, 127.9, 127.1, 125.8, 125.6, 125.3, 119.2, 118.2, 115.4, 114.2; R_f = 0.33 (EtOAc/Hexane1:3); HRMS(ESI+): Calcd for $C_{24}H_{16}F_3N_4O^+$ $[M+H]^+$ 433.1271, found 433.1266.

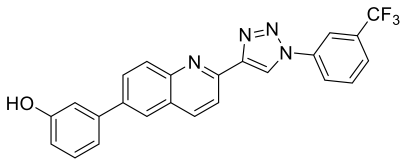

Compound 5: ^{1}H NMR (400 MHz, $CDCl_3$) : δ 9.39 (s, 1H), 8.59–8.50 (m, 2H), 8.50–8.38 (m, 3H), 8.22 (d, *J* = 2.7 Hz, 1H), 8.13–8.04 (m, 2H), 7.93 (d, *J* = 8.1 Hz, 2H), 7.38–7.29 (m, 3H), 6.92 (dd, *J* = 7.8, 2.3 Hz, 1H); ^{13}C NMR (100 MHz, $CDCl_3$): δ 157.3, 155.0, 154.0, 153.6, 145.9, 141.2, 133.1, 132.5, 130.4, 128.9, 127.8, 127.4, 127.3, 127.0, 124.8, 123.2, 122.7, 119.3, 115.1, 111.7, 108.3, 103.7, 102.0; R_f = 0.33 (EtOAc/Hexane1:3); LRMS(ESI+): Calcd for $C_{24}H_{16}F_3N_4O^+$ $[M+H]^+$ 433.1, found 433.1.

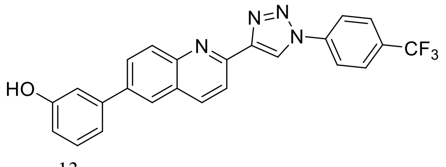

Compound 6: ^{1}H NMR (400 MHz, $(CD_3)_2SO$): δ 9.65 (s, 1H), 8.57 (d, *J* = 8.6 Hz, 1H), 8.36–8.30 (m, 3H), 8.25 (d, *J* = 1.8 Hz, 1H), 8.12–8.00 (m, 4H), 7.33 (t, *J* = 7.8 Hz, 1H), 7.26–7.24 (m, 1H), 7.21 (s, 1H), 6.84 (d, *J* = 7.9 Hz, 1H); ^{13}C NMR (100 MHz, $(CD_3)_2SO$): δ 158.1, 149.6, 148.7, 147.1, 140.8, 139.4, 138.5, 137.9, 130.4, 129.5, 129.2, 127.9, 127.4, 125.5, 125.3, 122.6, 122.4, 121.0, 119.0, 118.1, 115.2, 114.0; R_f = 0.33 (EtOAc/Hexane1:3); LRMS(ESI+): Calcd for $C_{24}H_{16}F_3N_4O^+$ $[M+H]^+$ 433.1, found 433.1.

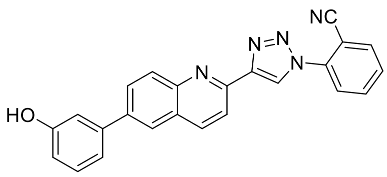

Compound 7: ^{1}H NMR (400 MHz, $(CD_3)_2SO$): δ 9.68 (s, 1H), 8.59 (d, *J* = 8.7 Hz, 1H), 8.33 (t, *J* = 7.5 Hz, 3H), 8.27 (s, 1H), 8.15–8.06 (m, 4H), 7.33 (t, *J* = 7.8 Hz, 1H), 7.26 (d, *J* = 7.7 Hz, 1H), 7.20 (s, 1H), 6.84 (d, *J* = 8.0 Hz, 1H); ^{13}C NMR (100 MHz, $(CD_3)_2SO$): δ 158.6, 150.0, 149.2, 147.6, 141.2, 140.0, 138.9, 138.3, 134.8, 130.7, 129.9, 129.5, 128.2, 125.8, 122.9, 122.8, 121.3 119.3 118.6 118.3 115.4 114.2, 111.7; R_f = 0.32 (EtOAc/Hexane1:3); LRMS(ESI+): Calcd for $C_{24}H_{16}N_5O^+$ $[M+H]^+$ 390.1, found 390.2.

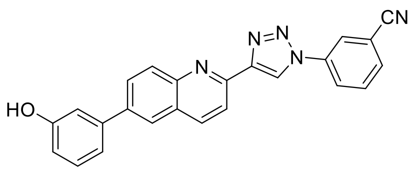

Compound 8: ^{1}H NMR (400 MHz, $(CD_3)_2SO$): δ 9.63–9.64 (m, 2H), 8.64 (s, 1H), 8.58 (d, *J* = 8.6 Hz, 1H), 8.46 (d, *J* = 8.2 Hz, 1H), 8.32 (d, *J* = 8.6 Hz, 1H), 8.27 (s, 1H), 8.10 (t, *J* = 6.8 Hz, 2H), 8.01 (d, *J* = 7.7 Hz, 1H), 7.85 (t, *J* = 8.0 Hz, 1H), 7.33 (t, *J* = 7.8 Hz, 1H), 7.26 (d, *J* = 7.7 Hz, 1H), 7.21 (s, 1H), 6.85 (d, *J* = 7.9 Hz, 1H); ^{13}C NMR (100 MHz, $(CD_3)_2SO$): δ 158.6, 150.1, 149.1, 147.6, 141.2, 138.9, 138.3, 137.5, 133.1, 131.9, 130.7, 129.9, 129.6, 128.3, 125.8, 125.5, 124.3, 122.9, 122.8, 119.2, 118.3, 115.4, 114.3, 113.3; R_f = 0.32 (EtOAc/Hexane1:3); LRMS(ESI+): Calcd for $C_{24}H_{16}N_5O^+$ $[M+H]^+$ 390.1, found 390.2.

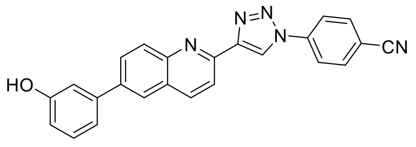

Compound 9: ^{1}H NMR (400 MHz, $(CD_3)_2SO$): δ 9.67 (d, *J* = 14.2 Hz, 2H), 8.60 (d, *J* = 8.6 Hz, 1H), 8.34 (t, *J* = 7.7 Hz, 3H), 8.28 (s, 1H), 8.15 (d, *J* = 8.8 Hz, 1H), 8.13–8.04 (m, 1H), 7.34 (t, *J* = 7.8 Hz, 1H), 7.26 (d, *J* = 7.7 Hz, 1H), 7.21 (s, 1H), 6.84 (d, *J* = 8.0 Hz, 1H); ^{13}C NMR (100 MHz, $(CD_3)_2SO$): δ 158.6, 150.4, 148.8, 147.6, 141.2, 138.7, 138.2, 137.1, 130.6, 130.4, 129.7, 129.5, 129.4, 128.2, 125.8, 122.4, 120.8, 119.2, 118.3, 115.4, 114.2; R_f = 0.32 (EtOAc/Hexane1:3); LRMS(ESI+): Calcd for $C_{24}H_{16}N_5O^+$ $[M+H]^+$ 390.1, found 390.1.

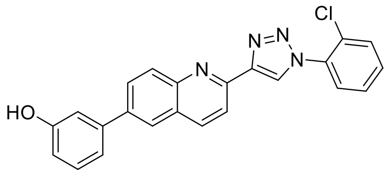

Compound 10: ^{1}H NMR (400 MHz, $(CD_3)_2SO$): δ 9.65 (s, 1H), 9.25 (s, 1H), 8.59 (d, *J* = 8.6 Hz, 1H), 8.34 (d, *J* = 8.6 Hz, 1H), 8.27 (s, 1H), 8.11–8.07 (m, 2H), 7.83 (d, *J* = 7.8 Hz, 3H), 7.71–7.62 (m, 3H), 7.33 (t, *J* = 7.8 Hz, 2H), 7.26 (d, *J* = 7.9 Hz, 2H), 7.22 (s, 1H), 6.84 (d, *J* = 7.9 Hz, 1H); ^{13}C NMR (100 MHz, $(CD_3)_2SO$): δ 158.6, 150.3, 148.0, 147.5, 141.2, 138.8, 138.2, 135.0, 133.7, 132.4, 131.1, 130.6, 129.7, 129.6, 129.3, 129.0, 128.2, 126.7, 125.8, 119.2, 118.2, 115.4, 114.2; R_f = 0.33 (EtOAc/Hexane1:3); LRMS(ESI+): Calcd for $C_{23}H_{16}ClN_4O^+$ $[M+H]^+$ 399.1, found 399.0.

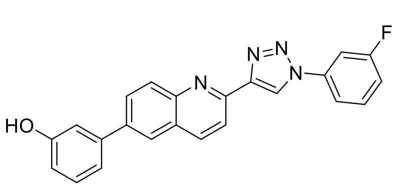

Compound 11: ^{1}H NMR (400 MHz, $(CD_3)_2SO$): δ 9.65 (s, 2H), 9.54 (s, 1H), 8.55 (d, *J* = 8.6 Hz, 2H), 8.30 (d, *J* = 8.5 Hz, 1H), 8.27–8.23 (m, 1H), 8.11–7.96 (m, 5H), 7.67 (q, *J* = 8.2 Hz, 2H), 7.42–7.28 (m, 4H), 7.25–7.21 (m, 2H), 6.84 (d, *J* = 8.0 Hz, 1H); ^{13}C NMR (100 MHz, $(CD_3)_2SO$): δ 164.3, 161.9, 158.6, 150.2, 148.9, 147.5, 141.2, 138.8, 138.2, 132.3, 130.6, 129.8, 129.5, 128.2, 125.8, 122.6, 119.2, 118.3, 116.6, 116.1, 115.9, 115.4, 114.2; R_f = 0.33 (EtOAc/Hexane1:3); LRMS(ESI+): Calcd for $C_{23}H_{16}FN_4O^+$ $[M+H]^+$ 383.1, found 383.1.

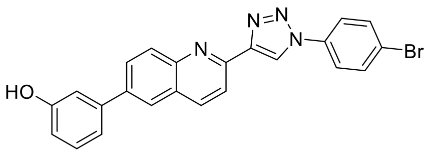

Compound 12: ^{1}H NMR (400 MHz, $(CD_3)_2SO$): δ 9.64 (s, 1H), 9.55 (s, 1H), 8.58 (d, *J* = 8.6 Hz, 1H), 8.32 (d, *J* = 8.6 Hz, 1H), 8.26 (d, *J* = 1.7 Hz, 1H), 8.14–8.03 (m, 4H), 7.85 (d, *J* = 8.9 Hz, 2H), 7.33 (t, *J* = 7.8 Hz, 1H), 7.26 (d, *J* = 8.0 Hz, 1H), 7.21 (t, *J* = 1.8 Hz, 1H), 6.84 (dd, *J* = 7.9, 1.4 Hz, 1H); ^{13}C NMR (100 MHz, $(CD_3)_2SO$): δ 158.6, 150.0, 149.2, 147.6, 141.2, 140.0, 138.9, 138.3, 134.8, 130.7, 129.9, 128.2, 125.8, 122.8, 121.3, 119.3, 118.6, 118.3, 115.4, 114.2, 111.7; R_f = 0.33 (EtOAc/Hexane1:3); LRMS(ESI+): Calcd for $C_{23}H_{16}BrN_4O^+$ $[M+H]^+$ 433.1, found 433.1.

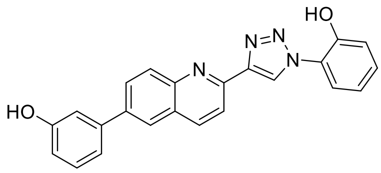

Compound 13:^{1}H NMR (400 MHz, CD_3OD): δ 9.31 (s, 1H), 8.71 (d, *J* = 8.7 Hz, 1H), 8.35 (d, *J* = 8.7 Hz, 1H), 8.22–8.13 (m, 3H), 7.76 (d, *J* = 8.0 Hz, 1H), 7.36 (t, *J* = 7.8 Hz, 1H), 7.29 (t, *J* = 7.8 Hz, 1H), 7.20–7.10 (m, 3H), 7.03 (t, *J* = 7.7 Hz, 1H), 6.82 (d, *J* = 8.0 Hz, 1H); ^{13}C NMR (100 MHz, CD_3OD): δ 159.3, 150.7, 146.1, 143.6, 141.9, 141.5, 134.9, 133.3, 131.8, 131.3, 129.8, 129.1, 128.2, 128.1, 126.7, 125.7, 125.5, 121.1, 120.5, 119.6, 118.1, 116.4, 115.1; R_f = 0.27 (EtOAc/Hexane1:3); LRMS(ESI+): Calcd for $C_{23}H_{17}N_4O_2^+$ $[M+H]^+$ 381.1, found 381.2.

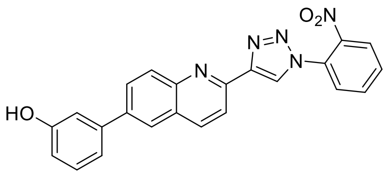

Compound 14: ^{1}H NMR (400 MHz, $(CD_3)_2SO$): δ 9.66 (s, 1H), 9.43 (s, 1H), 8.60 (d, *J* = 8.6 Hz, 1H), 8.35–8.24 (m, 3H), 8.13–7.98 (m, 4H), 7.9–7.85 (m, 1H), 7.89 (td, *J* = 7.7, 1.7 Hz, 1H), 7.33 (t, *J* = 7.8 Hz, 1H), 7.26 (d, *J* = 7.9 Hz, 1H), 7.22 (t, *J* = 2.1 Hz, 1H), 6.84 (d, *J* = 7.0 Hz, 1H); ^{13}C NMR (100 MHz, $(CD_3)_2SO$) δ 158.6, 150.1, 148.5, 147.5, 144.6, 141.2, 138.9, 138.3, 135.1, 132.0, 130.6, 129.8, 129.6, 129.6, 128.4, 128.2, 126.1, 125.9, 125.8, 119.2, 118.3, 115.4, 114.2; R_f = 0.29 (EtOAc/Hexane1:3); LRMS(ESI+): Calcd for $C_{23}H_{16}N_5O_3^+$ $[M+H]^+$ 410.1, found 410.1.

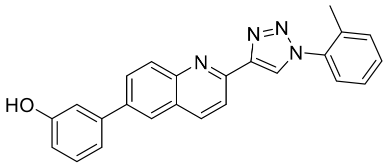

Compound 15: ^{1}H NMR (400 MHz, $(CD_3)_2SO$): δ 9.16 (s, 1H), 8.59 (d, *J* = 8.6 Hz, 1H), 8.33 (d, *J* = 8.6 Hz, 1H), 8.26 (s, 1H), 8.13–8.02 (m, 2H), 7.61–7.40 (m, 4H), 7.33 (t, *J* = 7.8 Hz, 1H), 7.25 (d, *J* = 7.2 Hz, 1H), 7.22 (d, *J* = 7.9 Hz, 1H), 2.25 (s, 3H); ^{13}C NMR (100 MHz, $(CD_3)_2SO$): δ 158.1, 149.9, 147.1, 146.8, 140.7, 138.3, 137.9, 136.2, 133.3, 131.5, 130.2, 130.1, 129.4, 129.0, 127.7, 127.1, 126.2, 125.7, 125.4, 118.9, 117.9, 115.0, 113.9, 17.6; R_f = 0.34 (EtOAc/Hexane1:3); LRMS(ESI+): Calcd for $C_{24}H_{19}N_4O^+$ $[M+H]^+$ 379.2, found 379.2.

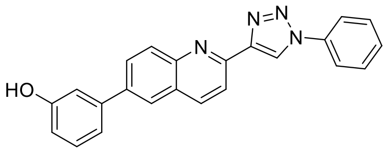

Compound 16: ^{1}H NMR (400 MHz, $(CD_3)_2SO$): δ 9.66 (s, 1H), 8.67 (d, *J* = 8.6 Hz, 1H), 8.33 (s, 1H), 8.23–8.10 (m, 3H), 8.08–8.02 (m, 2H), 7.93 (s, 1H), 7.58–7.57 (m, 3H), 7.35 (t, *J* = 7.8 Hz, 1H), 7.28 (d, *J* = 7.7 Hz, 1H), 7.23 (s, 1H), 6.86 (dd, *J* = 7.9, 2.4 Hz, 1H); ^{13}C NMR (100 MHz, $(CD_3)_2SO$): δ 158.6, 150.4, 148.8, 147.6, 141.2, 138.8, 138.2, 137.1, 130.6, 130.4, 129.8, 129.5, 129.4, 128.2, 125.8, 122.4, 120.8, 119.2, 118.3, 115.4, 114.2; R_f = 0.35 (EtOAc/Hexane1:3); LRMS(ESI+): Calcd for $C_{23}H_{17}N_4O^+$ $[M+H]^+$ 365.1, found 365.2.

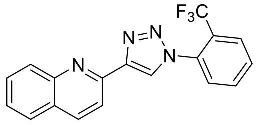

Compound 17: ^{1}H NMR (500 MHz, $CDCl_3$) δ 8.95 (s, 1H), 8.38 (d, *J* = 8.5 Hz, 1H), 8.27 (d, *J* = 8.5 Hz, 1H), 8.08 (d, *J* = 8.5 Hz, 1H), 7.90 – 7.88 (m, 1H), 7.84 – 7.79 (m, 2H), 7.72 (t, *J* = 7.7 Hz, 1H), 7.62 (td, *J* = 7.6, 1.2 Hz, 1H), 7.53 (t, *J* = 7.5 Hz, 1H).; ^{13}C NMR (100 MHz, $CDCl_3$): δ 150.2, 148.7, 148.3, 137.1, 135.0, 131.1, 131.0, 129.9, 129.3, 129.1, 128.1, 128.0, 127.9, 127.8, 126.6, 125.6, 125.0, 118.9; R_f = 0.41 (EtOAc/Hexane1:3); LRMS(ESI+): Calcd for $C_{18}H_{12}F_3N_4^+$ $[M+H]^+$ 341.1, found 341.0.

III. Supplementary Tables

Supplementary Table 1. Structure and activity relationship (SAR) study over LD reduction activity

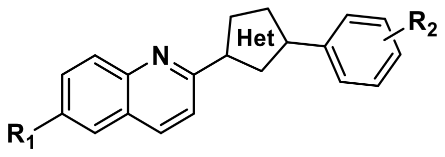

Cpd.	R_1	Het	R_2	LD reduction (%) Efficacy	LD reduction (IC_{50}, µM) Potency
**1**	3-Hydroxyphenyl	1,4-triazole	2-OCH_3	50.0	6.7
**2**[a, b]	3-Hydroxyphenyl	1,4-triazole	3-OCH_3	29.8	10.2
**3**	3-Hydroxyphenyl	1,4-triazole	4-OCH_3	19.0	3.7
**4**	3-Hydroxyphenyl	1,4-triazole	2-CF_3	64.5	4.4
**5**[b]	3-Hydroxyphenyl	1,4-triazole	3-CF_3	46.6	N/A
**6**	3-Hydroxyphenyl	1,4-triazole	4-CF_3	35.9	32.0
**7**	3-Hydroxyphenyl	1,4-triazole	2-CN	30.4	14.8
**8**[a]	3-Hydroxyphenyl	1,4-triazole	3-CN	-7.1	27.6
**9**[b]	3-Hydroxyphenyl	1,4-triazole	4-CN	-3.9	N/A
**10**[a]	3-Hydroxyphenyl	1,4-triazole	2-Cl	2.7	10.1
**11**	3-Hydroxyphenyl	1,4-triazole	3-F	19.2	20.6
**12**[a]	3-Hydroxyphenyl	1,4-triazole	4-Br	36.8	4.7
**13**	3-Hydroxyphenyl	1,4-triazole	2-OH	12.7	11.6
**14**[a]	3-Hydroxyphenyl	1,4-triazole	2-NO_2	48.5	7.9
**15**[b]	3-Hydroxyphenyl	1,4-triazole	2-CH_3	40.9	2.6
**16**[b]	3-Hydroxyphenyl	1,4-triazole	H	7.0	>1000
**17**[b]	H	1,4-triazole	2-CF_3	-5.6	>1000
**18**[b]	3-Hydroxyphenyl	isoxazole	2-CF_3	42.3	9.5
**19**	H	isoxazole	2-CF_3	-6.7	40.0
**20**[a]	3-Hydroxyphenyl	isoxazole	H	47.6	26.0

[a] Cell's morphology was changed
[b] Compound was insoluble in media at 10 µM
N/A; Not applicable

Supplementary Table 2. Identified target protein list from LC/MS/MS

Spot No.	Match to	Molecular Weight	Mascot Score	Queries matched	Sequence Coverage (%)	Protein
①	ACSL4_HUMAN	79137	428	47	37	Long-chain-fatty-acid--CoA ligase 4
	DDX17_HUMAN	72326	278	21	30	Probable ATP-dependent RNA helicase DDX17
	DHB4_HUMAN	79636	101	7	9	Peroxisomal multifunctional enzyme type 2
	LDHA_HUMAN	36665	98	3	9	L-lactate dehydrogenase A chain
②	ST1A1_HUMAN	34175	165	24	20	Sulfotransferase 1A1
③-1	ERG7_HUMAN	83255	321	19	25	Lanosterol synthase
	ALBU_HUMAN	69321	82	5	6	Serum albumin precursor
③-2	PRDX3_HUMAN	27675	181	11	20	Thioredoxin-dependent peroxide reductase, mitochondrial precursor
③-3	WDR1_HUMAN	66152	345	30	27	WD repeat protein 1
④-1	PAPS1_HUMAN	70788	949	73	36	Bifunctional 3'-phosphoadenosine 5'-phosphosulfate synthetase 1
④-2	PCY2_HUMAN	43808	541	48	44	Ethanolamine-phosphate cytidylyltransferase
	PRS8_HUMAN	45597	96	2	5	26S protease regulatory subunit 8
⑤	PCY2_HUMAN	43808	654	43	44	Ethanolamine-phosphate cytidylyltransferase
	IDHC_HUMAN	46630	480	31	37	Isocitrate dehydrogenase [NADP] cytoplasmic
⑥	PRDX3_HUMAN	27675	1815	169	41	Thioredoxin-dependent peroxide reductase, mitochondrial precursor

Supplementary Table 3. qPCR primer and si-RNA sequences

[qPCR primer sequences]

Gene	Protein	Forward primer sequence	Reverse primer sequence
GAPDH	GAPDH	AGGGCTGCTTTTAACTCTGGT	CCCCACTTGATTTTGGAGGGA
PLIN2 / ADFP	Perilipin-2 / ADFP	CTCATGGGTAGAGTGGAAAAGGAGCATTGG	TTGGATGTTGGACAGGAGGGTGTGGCACGT
MLXIPL	Carbohydrate-responsive element-binding protein / ChREBP	GTCACGAAGCCACACACG	GAGACAAGATCCGCCTGAAC
LPIN1	Phosphatidate phosphatase LPIN1	GATGTCAATGCACCCTGAGA	GTGTTTGCAATACAAAGGCG
LSS	Lanosterol synthase	TATTTCCACAAGCGTTTCCC	TGAAGCAAACTCCCCAGG
PPARA	Peroxisome proliferator-activated receptor alpha / PPAR-alpha	CATTACGGAGTCCACGCGT	ACCAGCTTGAGTCGAATCGTT
PPARG	Peroxisome proliferator-activated receptor gamma / PPAR-gamma	GATGACAGCGACTTGGCAA	CTTCAATGGGCTTCACATTCA
SCD	Stearoyl-CoA desaturase / hSCD1	TGGGTTGGCTGCTTGTG	GCGTGGGCAGGATGAAG
SREBF1	Sterol regulatory element-binding protein 1 / SREBP-1	AGGGAAGTCACTGTCTTGGTTG	CTGCTGACCGACATCGAA
THRSP	Thyroid hormone-inducible hepatic protein	AGGCCTTTCTGCTCTCATCA	AAATGACGGGACAAGTTTGG
Mitochondrial DNA targeting gene		CCCACAAACCCCATTACTAAACCCA	TTTCATCATGCGGAGATTGTTGGATGG

[si-RNA sequences]

Gene	Sense	antisense
Atg5 (Ambion, s18158)	AUG AGC UUC AAU UGC AUC CTT	AAG GAU GCA AUU GAA GCU CAU
Acsl4 (Bioneer, 2182-1)	CA CAC ACU UCG ACU CAC UT	UA GUG AGU CGA AGU GUG UG
Pcyt2 (Bioneer, 5833-2)	CU CAU CGU CCA GCG GAU CA	UG AUC CGC UGG ACG AUG AG
Idh1 (Bioneer, 3417-1)	GC UGU GCA UUA AAC UUG CA	UG CAA GUU UAA UGC ACA GC
Ubb (Bioneer, 7314-1)	GCC GUA CUC UUU CUG ACU A	UAG UCA GAA AGA GUA CGG C

IV. Copies of ^{1}H and ^{13}C NMR Spectra

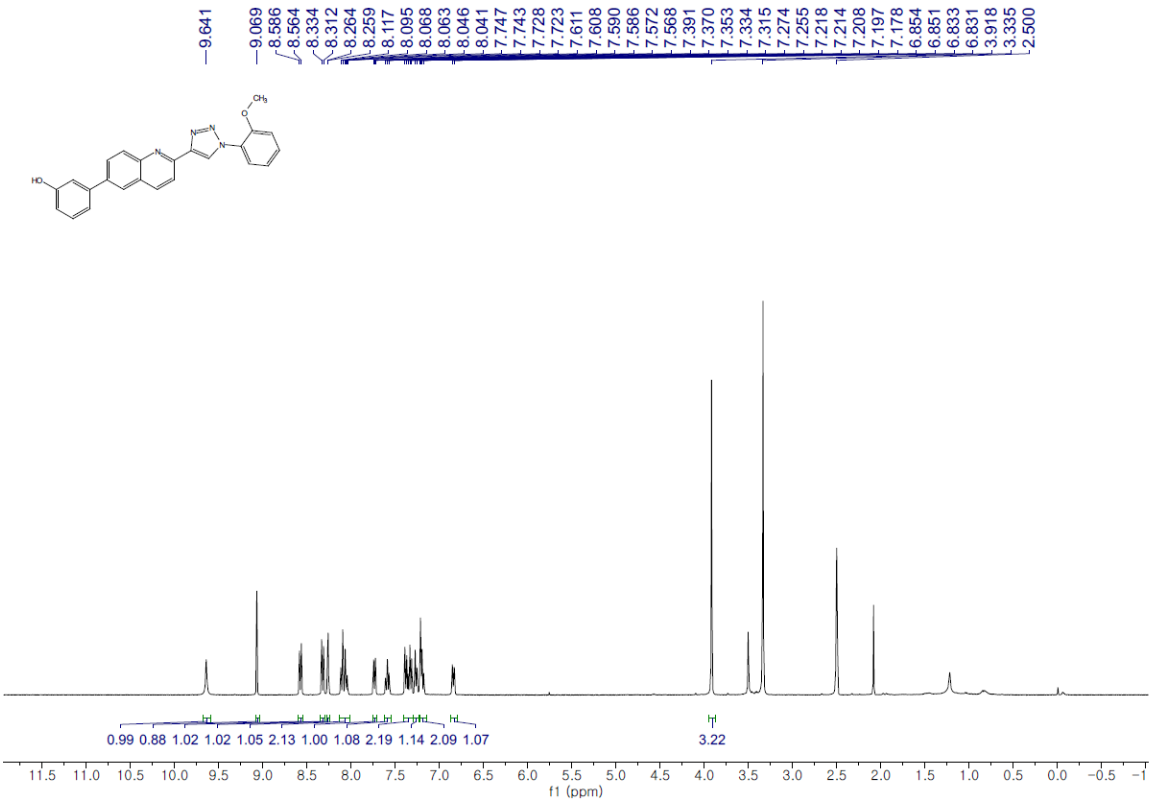

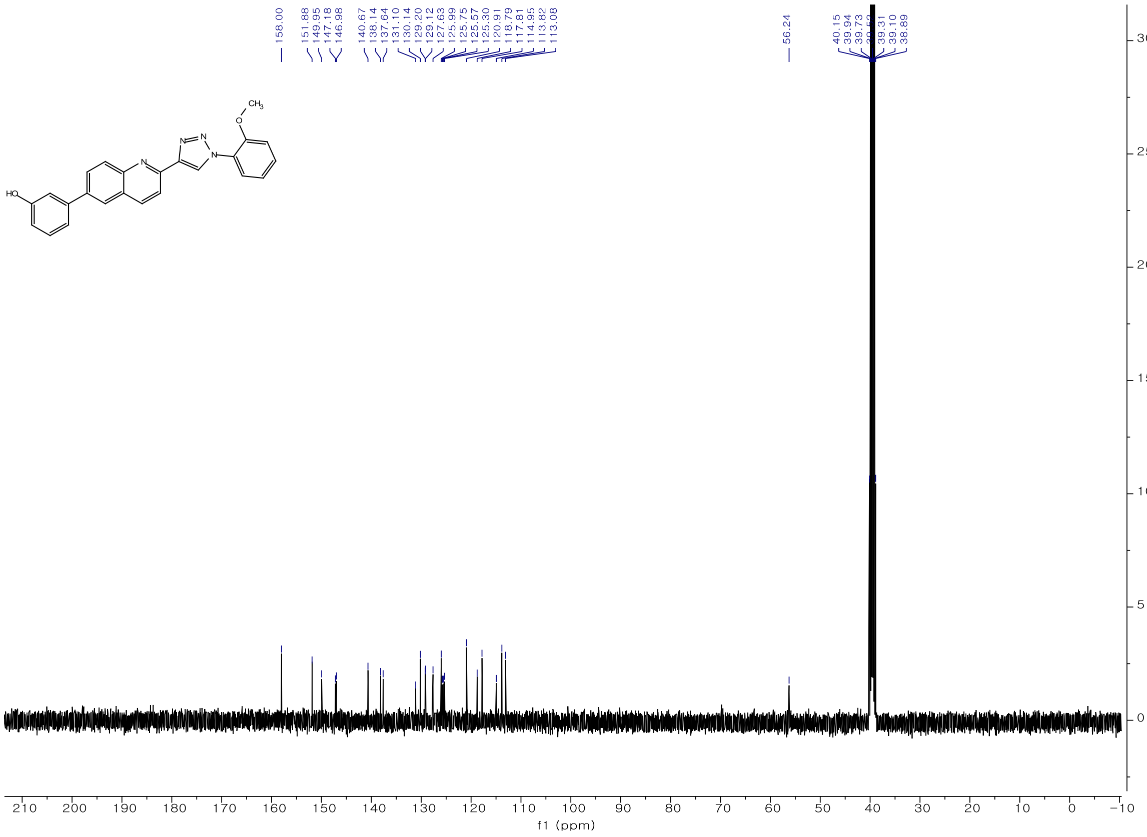

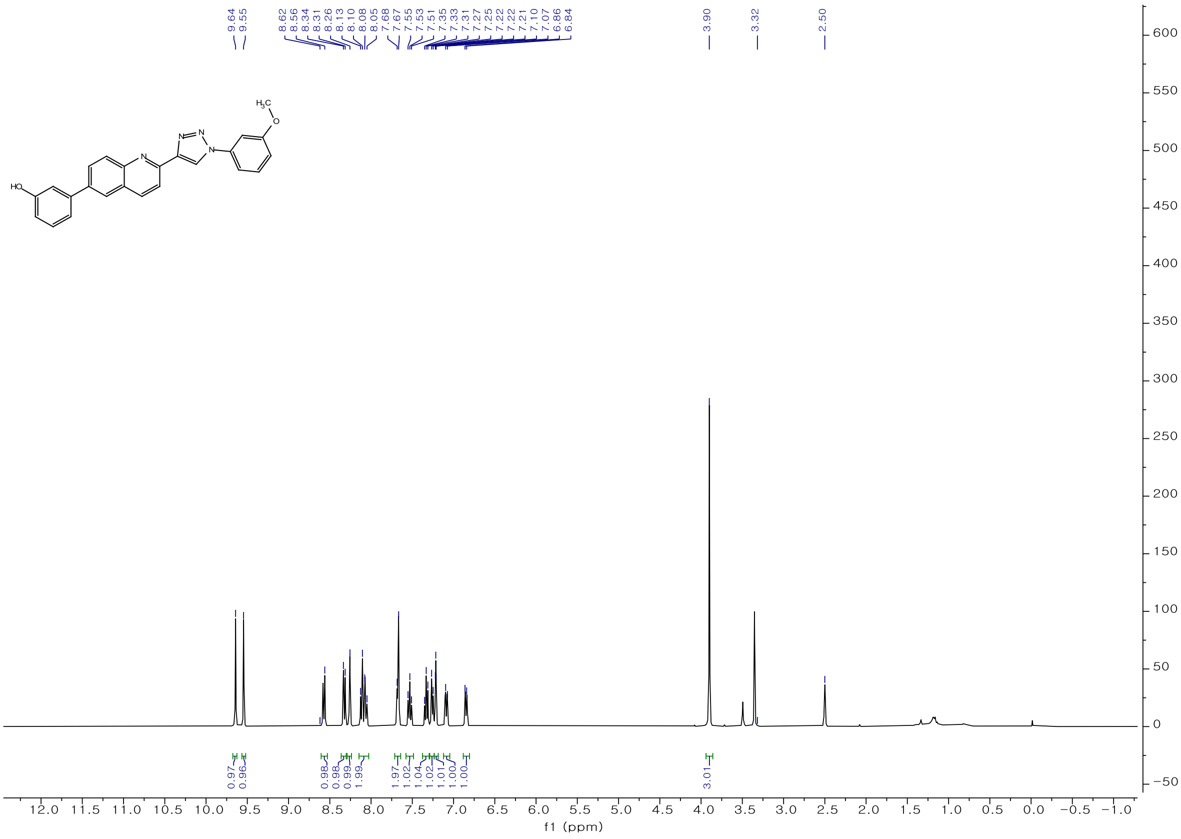
9.64
9.55
8.62
8.56
8.34
8.31
8.26
8.13
8.10
8.08
8.05
7.68
7.67
7.55
7.53
7.51
7.35
7.33
7.31
7.27
7.25
7.22
7.22
7.21
7.10
7.07
6.86
6.84
3.90
3.32
2.50
H_3C
O
N
N
N
N
HO
0.97
0.96
0.98
0.98
0.99
1.99
1.97
1.02
1.04
1.02
1.01
1.00
1.00
3.01
f1 (ppm)

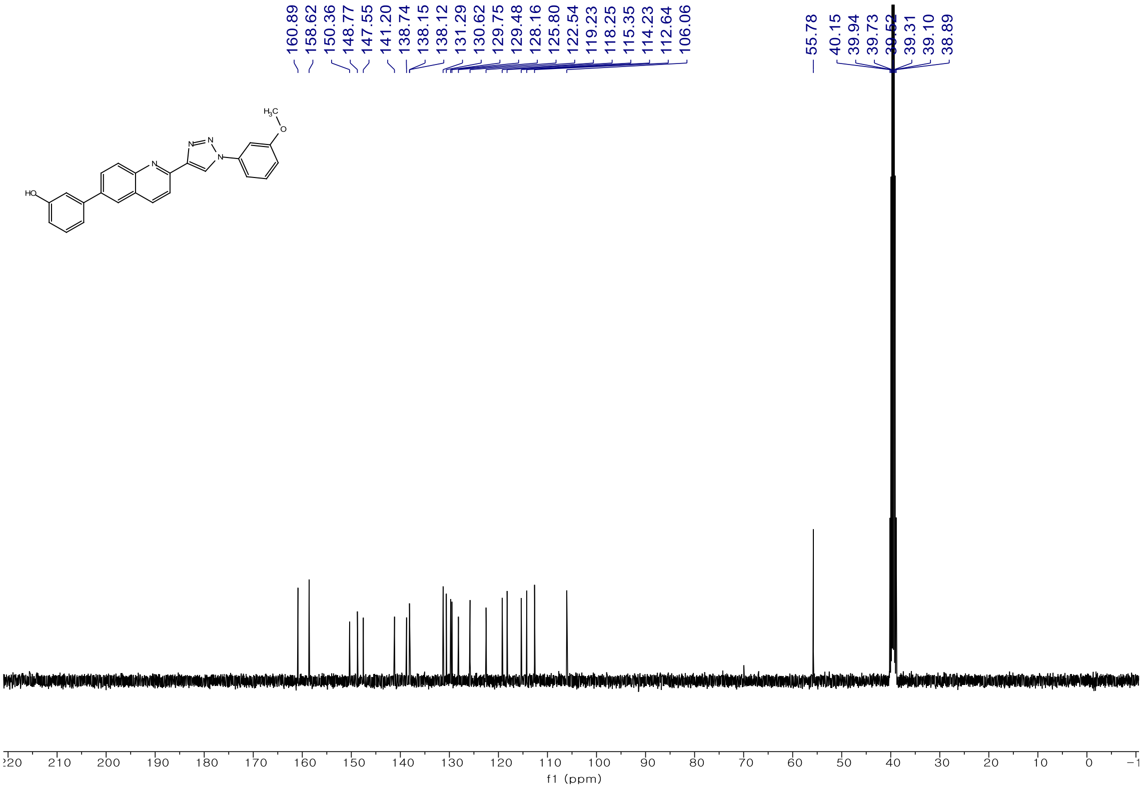
160.89
158.62
150.36
148.77
147.55
141.20
138.74
138.15
138.12
131.29
130.62
129.75
129.48
128.16
125.80
122.54
119.23
118.25
115.35
114.23
112.64
106.06
55.78
40.15
39.94
39.73
39.52
39.31
39.10
38.89
H_3C
O
N
N
N
N
HO
f1 (ppm)

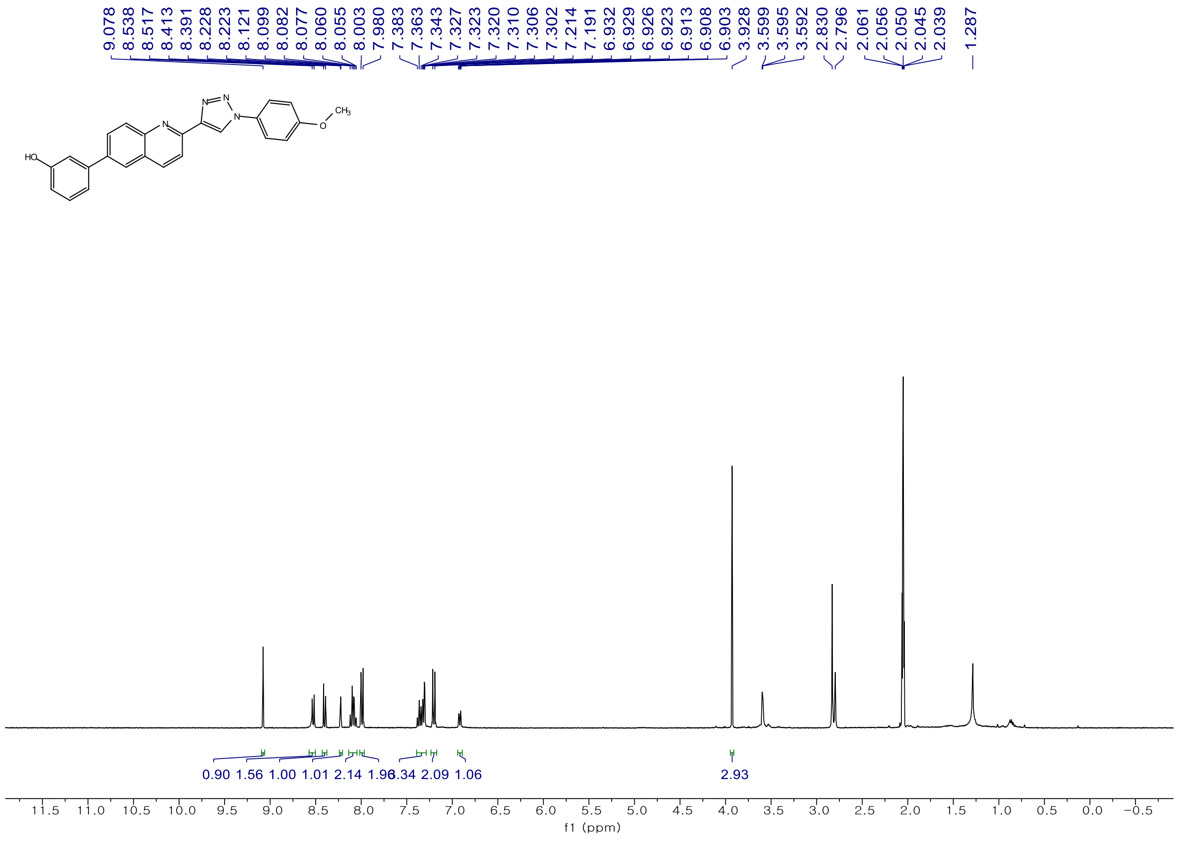

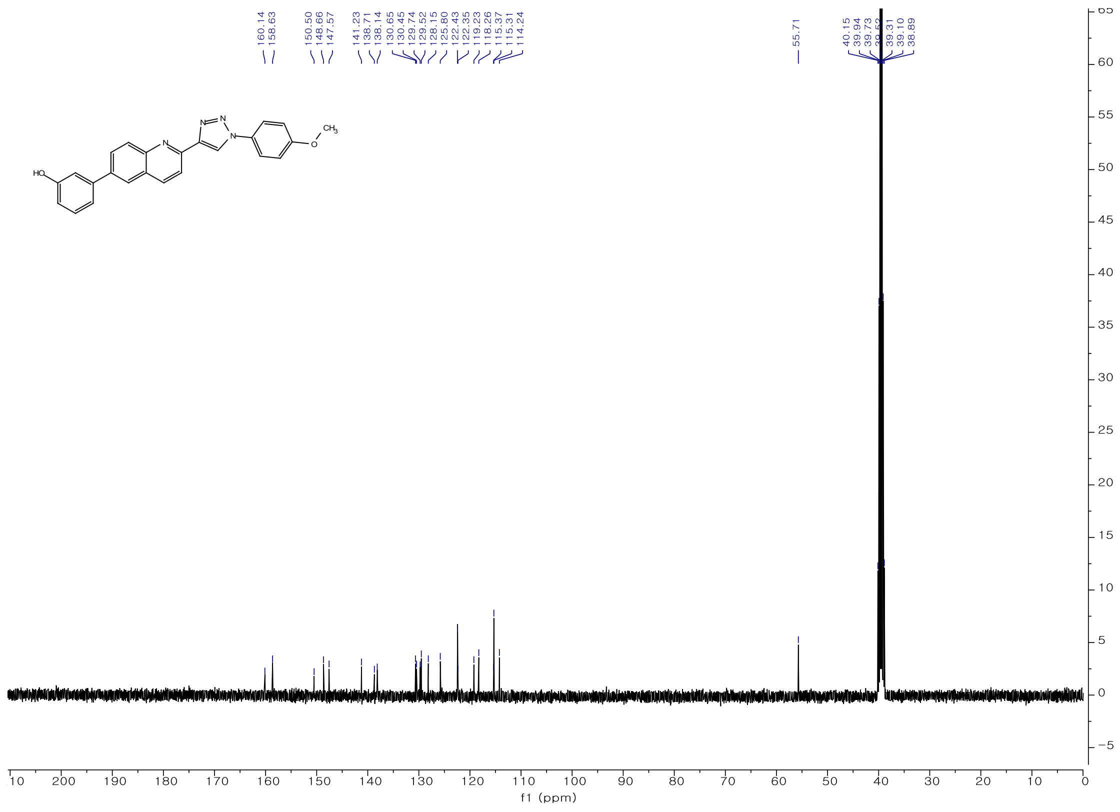

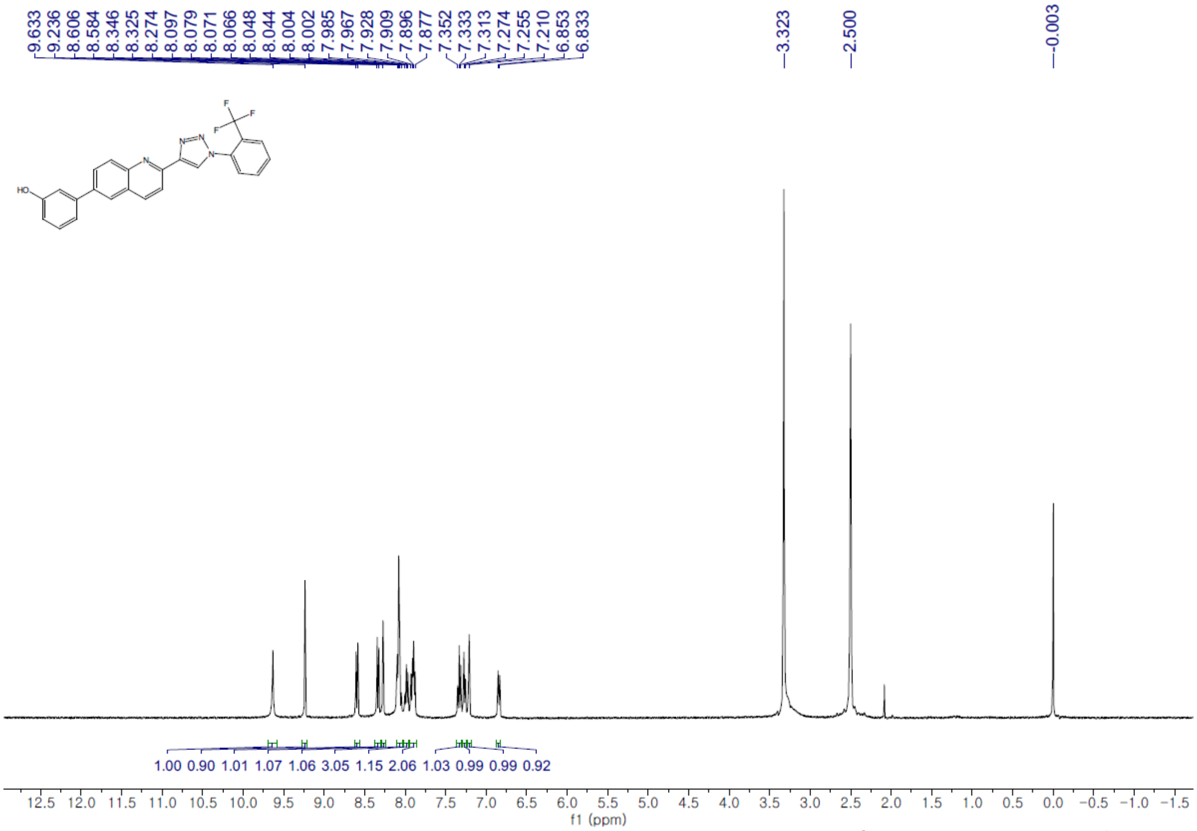
9.633 9.236 8.606 8.584 8.346 8.325 8.274 8.097 8.079 8.071 8.066 8.048 8.044 8.004 8.002 7.985 7.967 7.928 7.909 7.896 7.877 7.352 7.333 7.313 7.274 7.255 7.210 6.853 6.833
3.323
2.500
-0.003
HO
N
N
N
N
F
F
F
1.00 0.90 1.01 1.07 1.06 3.05 1.15 2.06 1.03 0.99 0.99 0.92
12.5 12.0 11.5 11.0 10.5 10.0 9.5 9.0 8.5 8.0 7.5 7.0 6.5 6.0 5.5 5.0 4.5 4.0 3.5 3.0 2.5 2.0 1.5 1.0 0.5 0.0 -0.5 -1.0 -1.5
f1 (ppm)

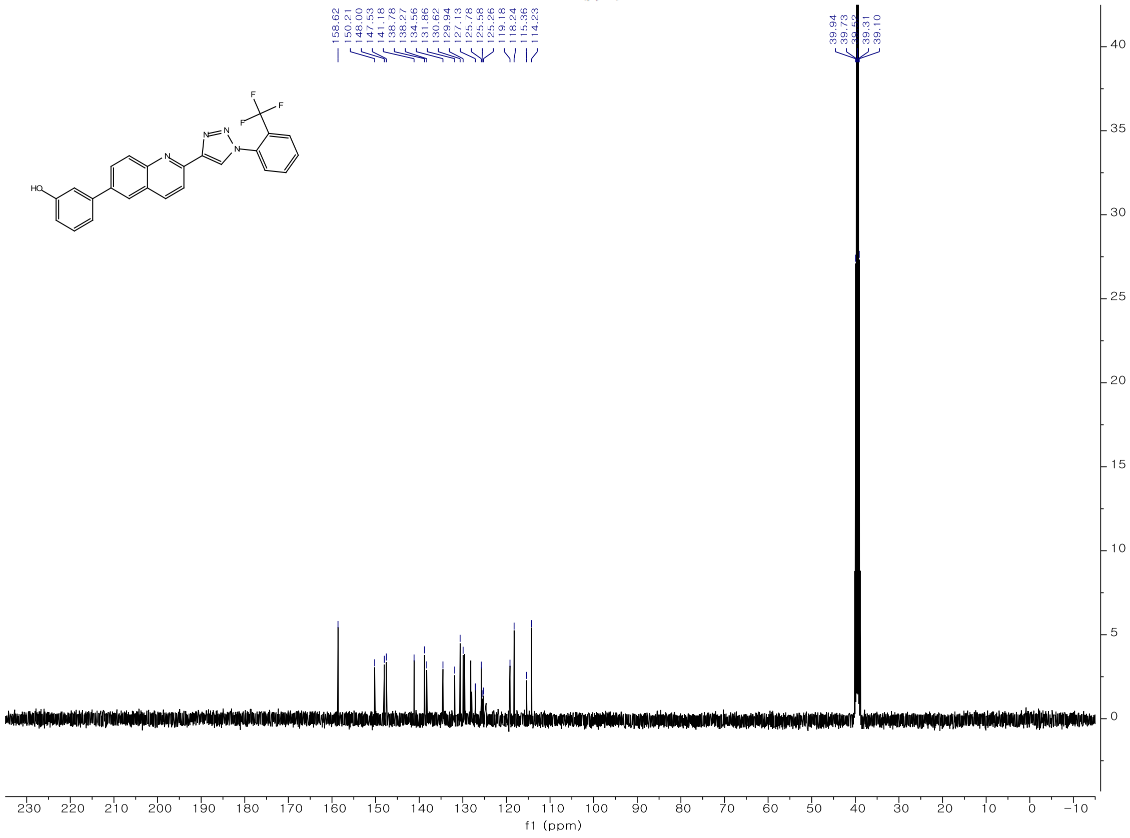
158.62 150.21 148.00 147.53 141.18 138.78 138.27 134.56 131.86 130.62 129.94 127.13 125.78 125.58 125.26 119.18 118.24 115.36 114.23
39.94 39.73 39.52 39.31 39.10
HO
N
N
N
N
F
F
F
40
35
30
25
20
15
10
5
0
230 220 210 200 190 180 170 160 150 140 130 120 110 100 90 80 70 60 50 40 30 20 10 0 -10
f1 (ppm)

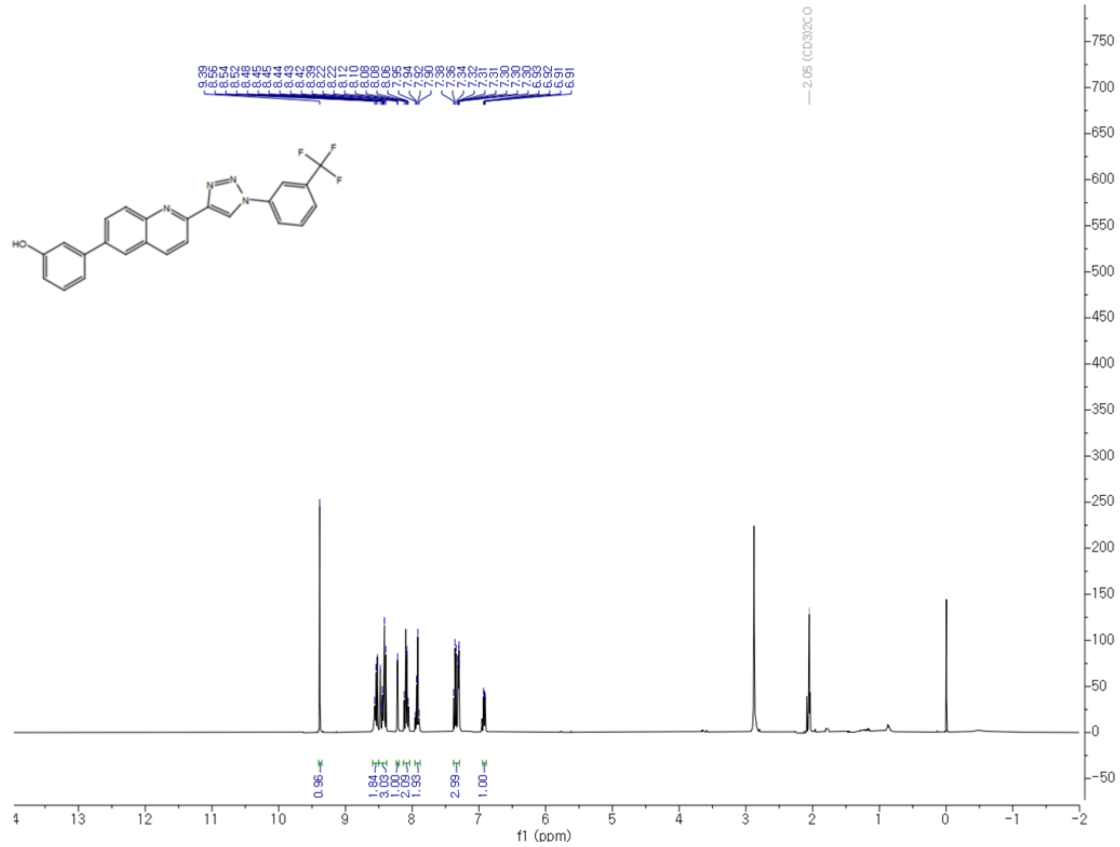

9.39 8.56 8.54 8.52 8.48 8.45 8.44 8.43 8.42 8.39 8.22 8.22 8.12 8.10 8.08 8.06 7.95 7.94 7.92 7.90 7.38 7.36 7.34 7.32 7.31 7.30 7.30 7.30 6.93 6.92 6.91 6.91
2.05 (CD3)2CO
HO
N
F
0.96
1.84
3.03
1.00
2.09
1.93
2.99
1.00
f1 (ppm)

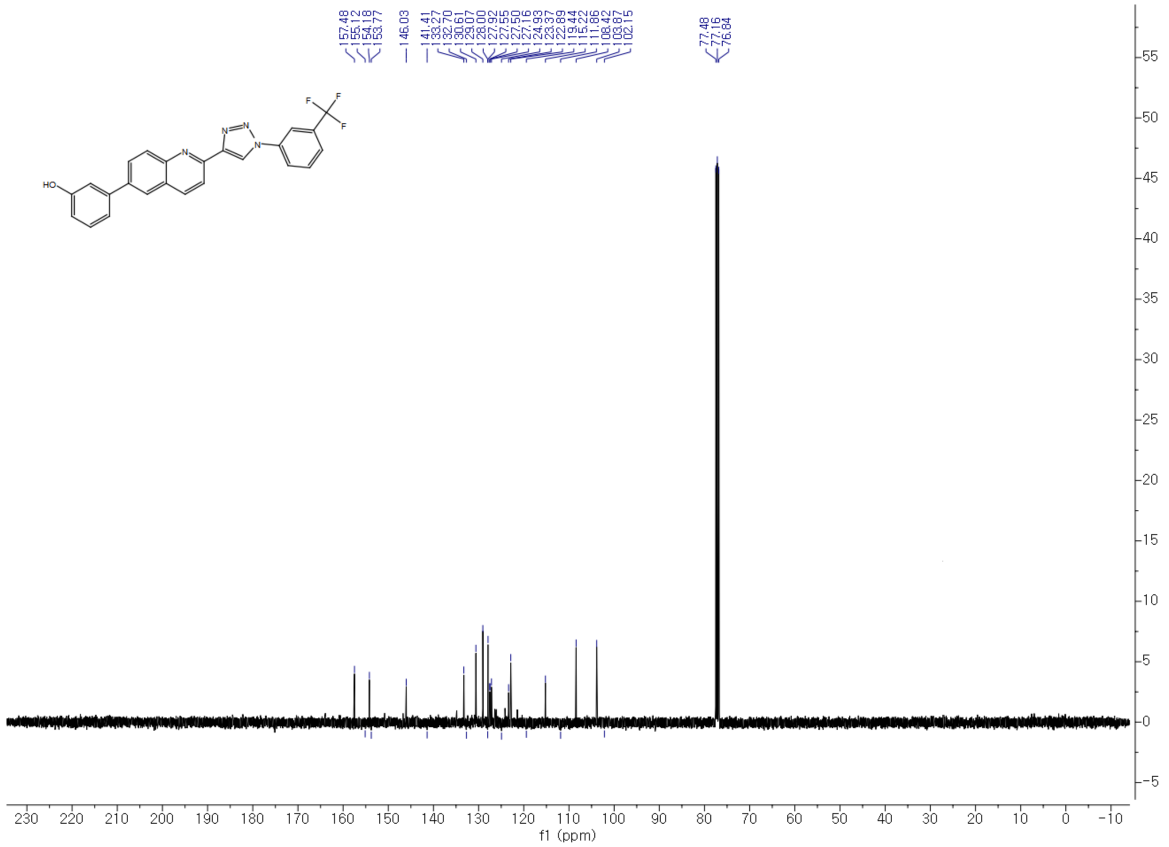

157.48 155.12 154.18 153.77
146.03
141.41 133.27 132.70 130.61 130.07 128.00 127.92 127.55 127.50 127.16 124.93 123.37 122.89 119.44 115.22 111.86 108.42 103.87 102.15
77.48 77.16 76.84
HO
N
F
f1 (ppm)

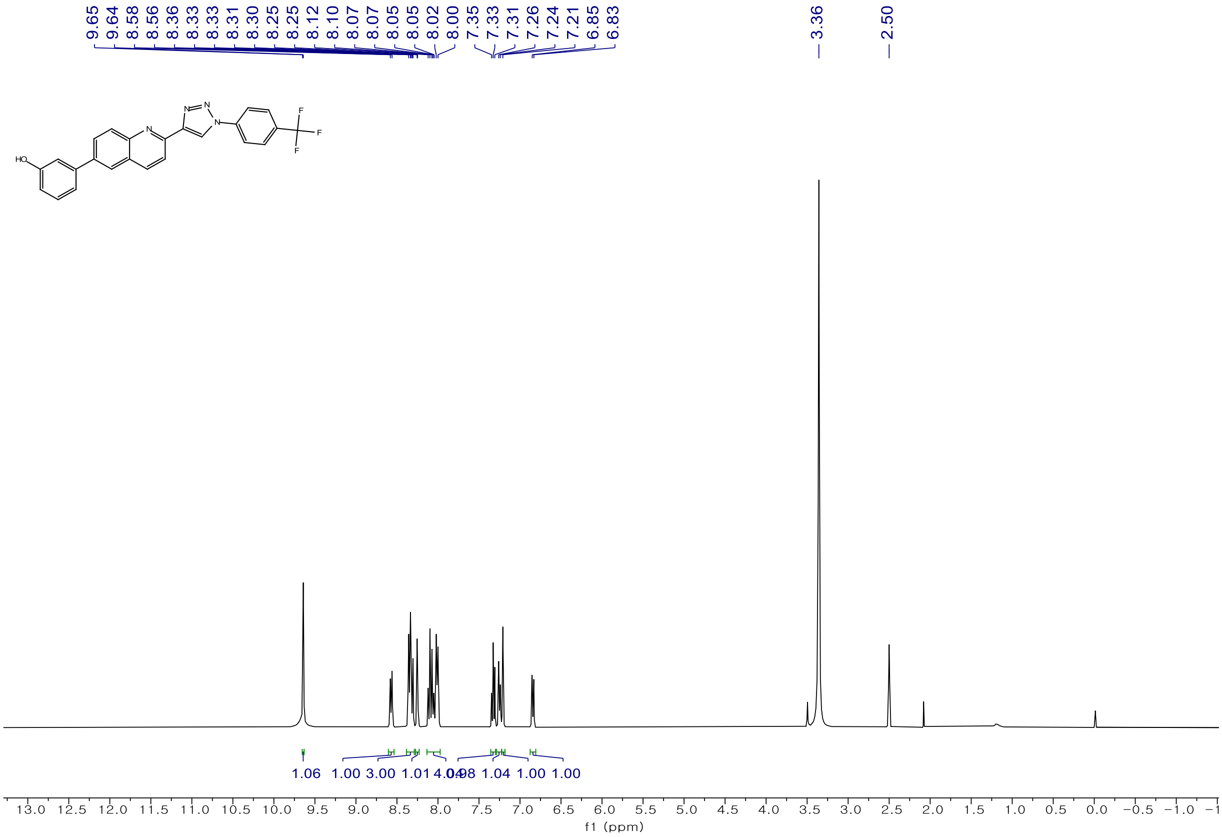
9.65 9.64 8.58 8.56 8.36 8.33 8.33 8.31 8.30 8.25 8.12 8.10 8.07 8.05 8.05 8.02 8.00 7.35 7.33 7.31 7.26 7.24 7.21 6.85 6.83
3.36
2.50
HO
N
N=N
F
F
F
1.06 1.00 3.00 1.01 4.04 0.98 1.04 1.00 1.00
f1 (ppm)

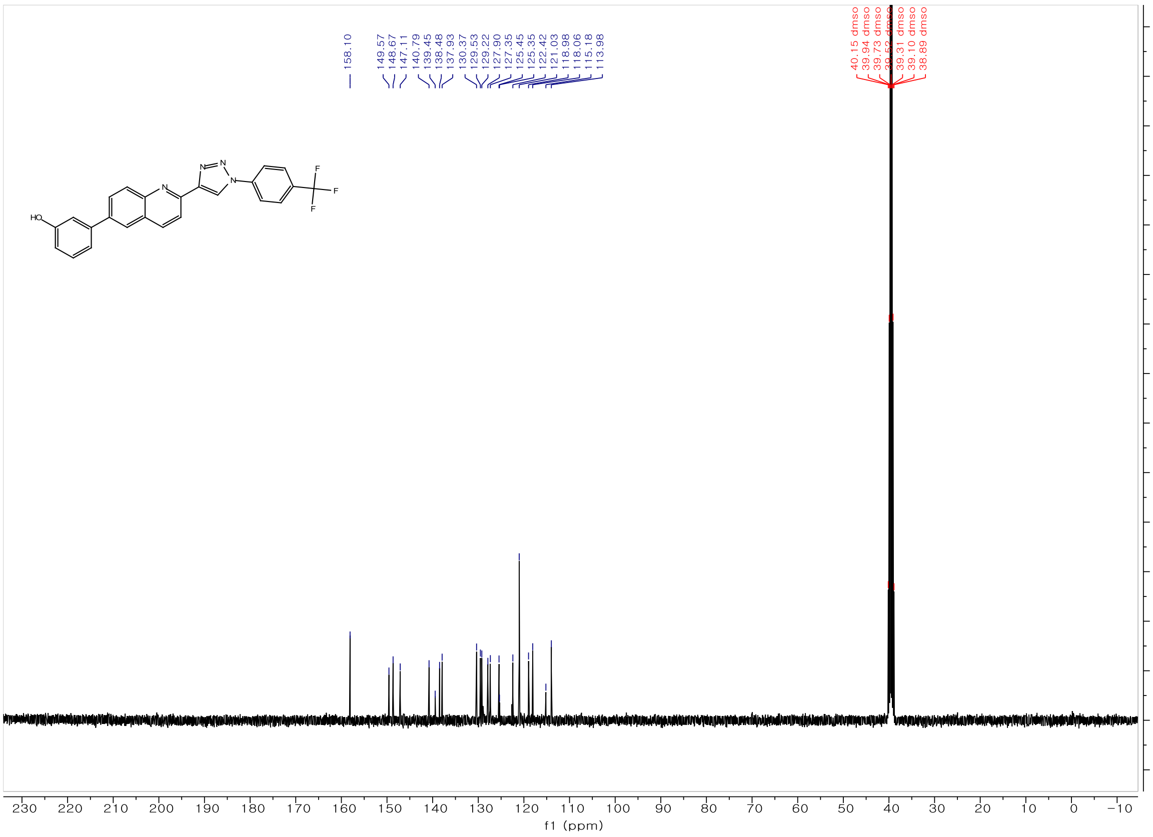
158.10
149.57 148.67 147.11 140.79 139.45 138.48 137.93 130.37 129.53 129.22 127.90 127.35 125.45 125.35 122.42 121.03 118.98 118.06 115.18 113.98
40.15 dmso 39.94 dmso 39.73 dmso 39.52 dmso 39.31 dmso 39.10 dmso 38.89 dmso
HO
N
N=N
F
F
F
f1 (ppm)

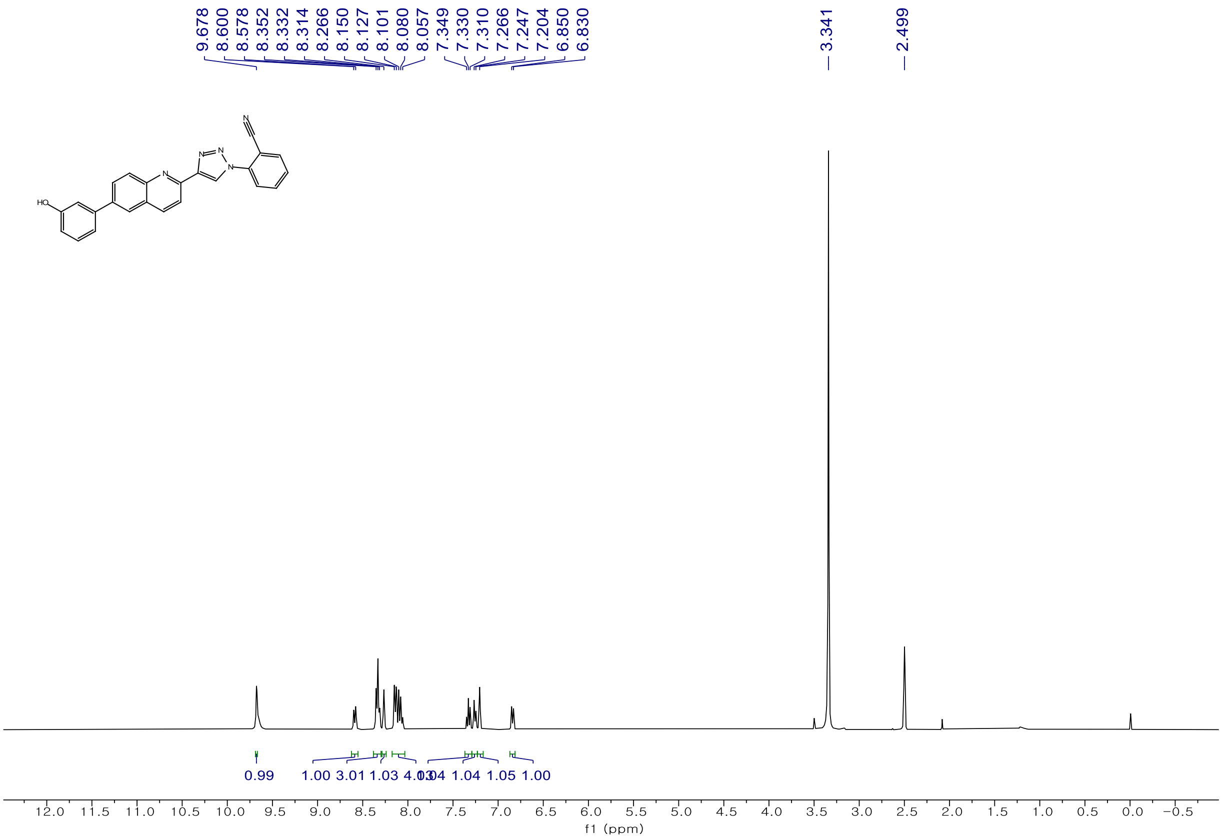
9.678
8.600
8.578
8.352
8.332
8.314
8.266
8.150
8.127
8.101
8.080
8.057
7.349
7.330
7.310
7.266
7.247
7.204
6.850
6.830
3.341
2.499
HO
N
0.99
1.00 3.01 1.03 4.03 1.04 1.04 1.05 1.00
12.0 11.5 11.0 10.5 10.0 9.5 9.0 8.5 8.0 7.5 7.0 6.5 6.0 5.5 5.0 4.5 4.0 3.5 3.0 2.5 2.0 1.5 1.0 0.5 0.0 −0.5
f1 (ppm)

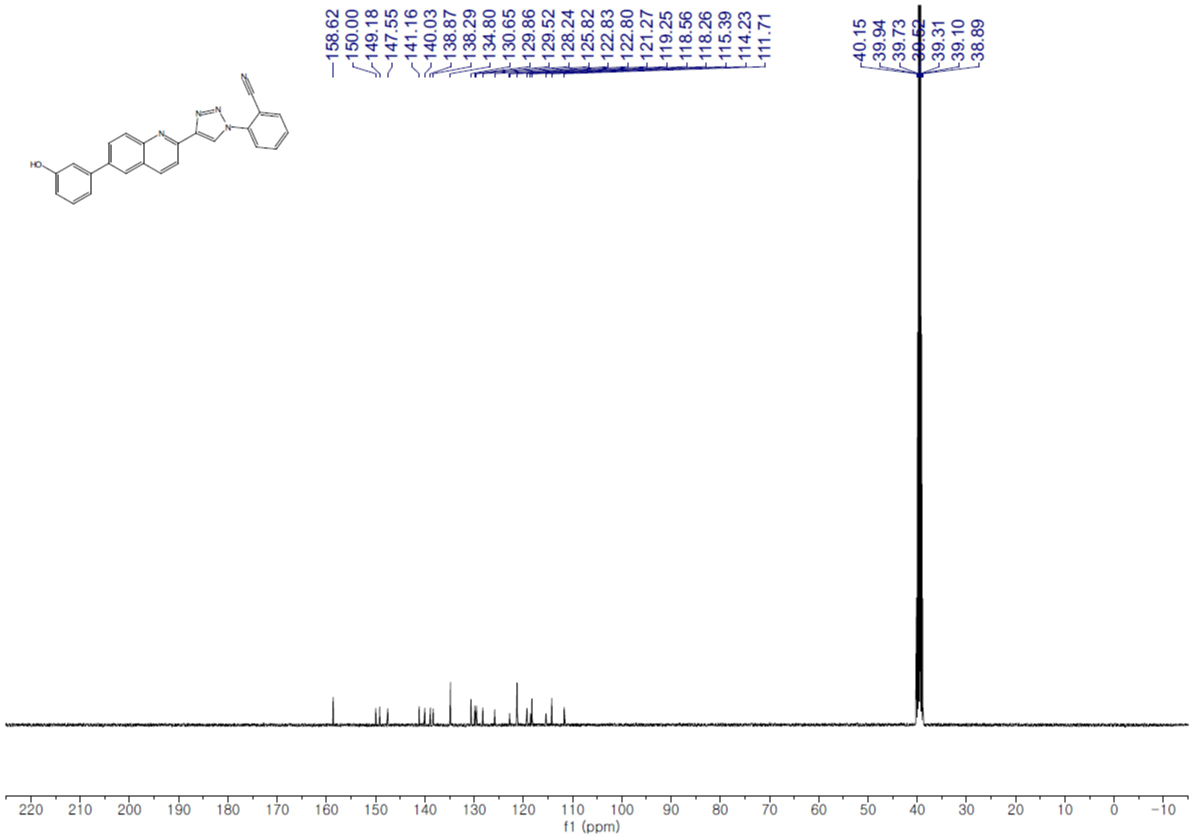
158.62
150.00
149.18
147.55
141.16
140.03
138.87
138.29
134.80
130.65
129.86
129.52
128.24
125.82
122.83
122.80
121.27
119.25
118.56
118.26
115.39
114.23
111.71
40.15
39.94
39.73
39.52
39.31
39.10
38.89
HO
N
220 210 200 190 180 170 160 150 140 130 120 110 100 90 80 70 60 50 40 30 20 10 0 −10
f1 (ppm)

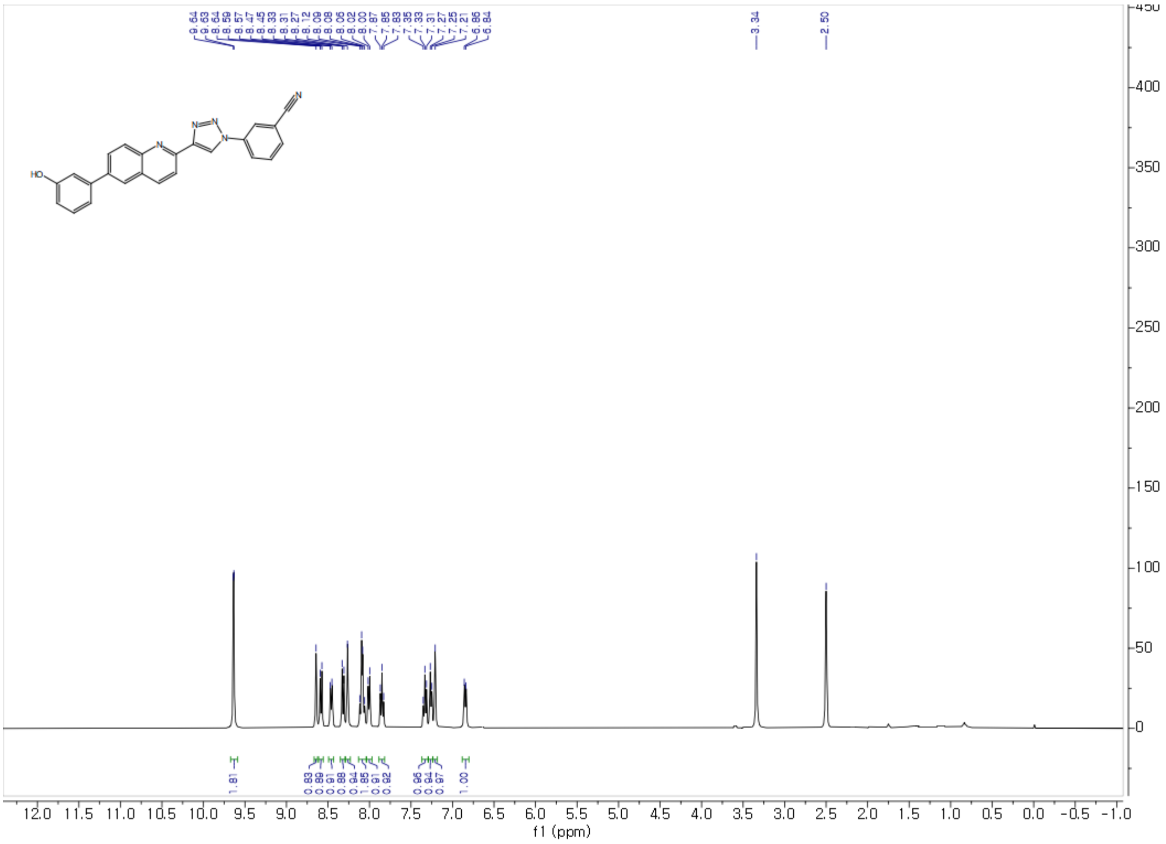

9.64
9.63
8.64
8.59
8.57
8.47
8.45
8.33
8.31
8.27
8.12
8.09
8.08
8.05
8.02
8.00
7.97
7.85
7.83
7.35
7.33
7.31
7.27
7.25
7.21
6.86
6.84
3.34
2.50
HO
N
N=N
N
f1 (ppm)

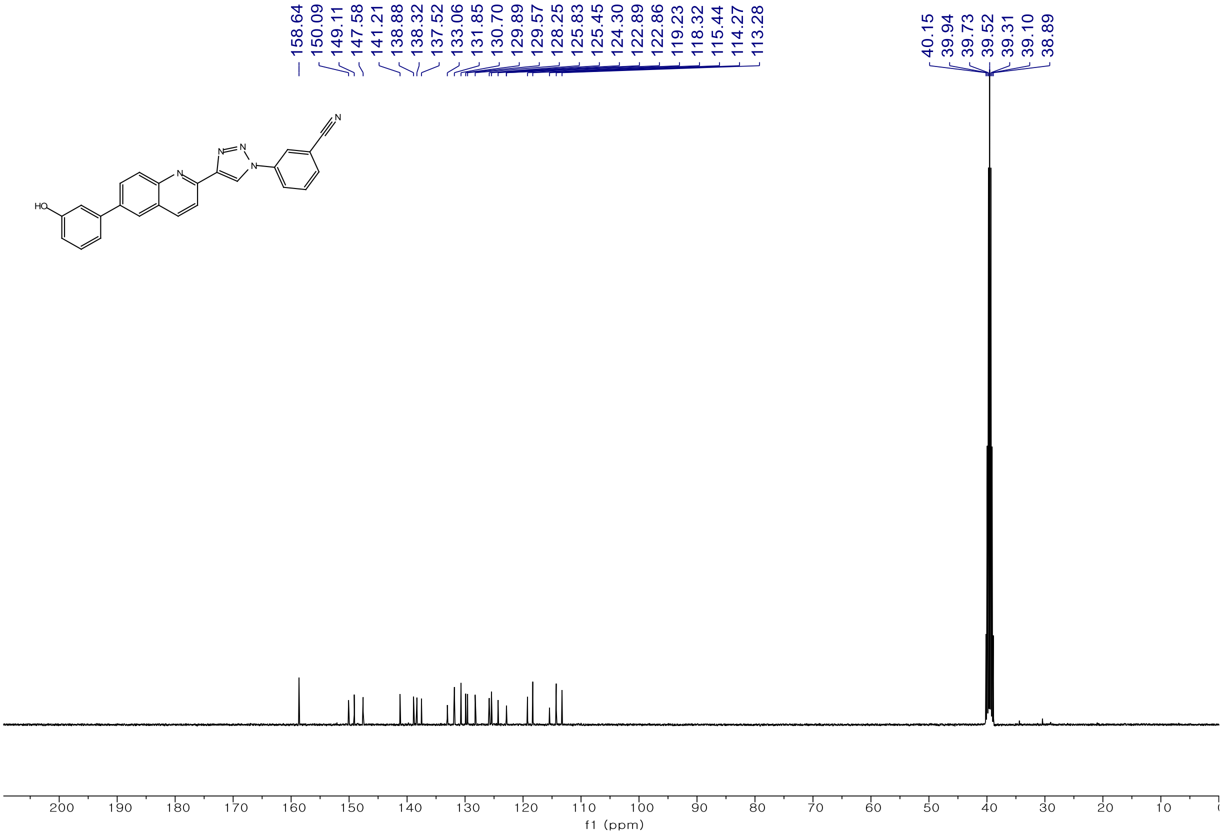

158.64
150.09
149.11
147.58
141.21
138.88
138.32
137.52
133.06
131.85
130.70
129.89
129.57
128.25
125.83
125.45
124.30
122.89
122.86
119.23
118.32
115.44
114.27
113.28
40.15
39.94
39.73
39.52
39.31
39.10
38.89
HO
N
f1 (ppm)

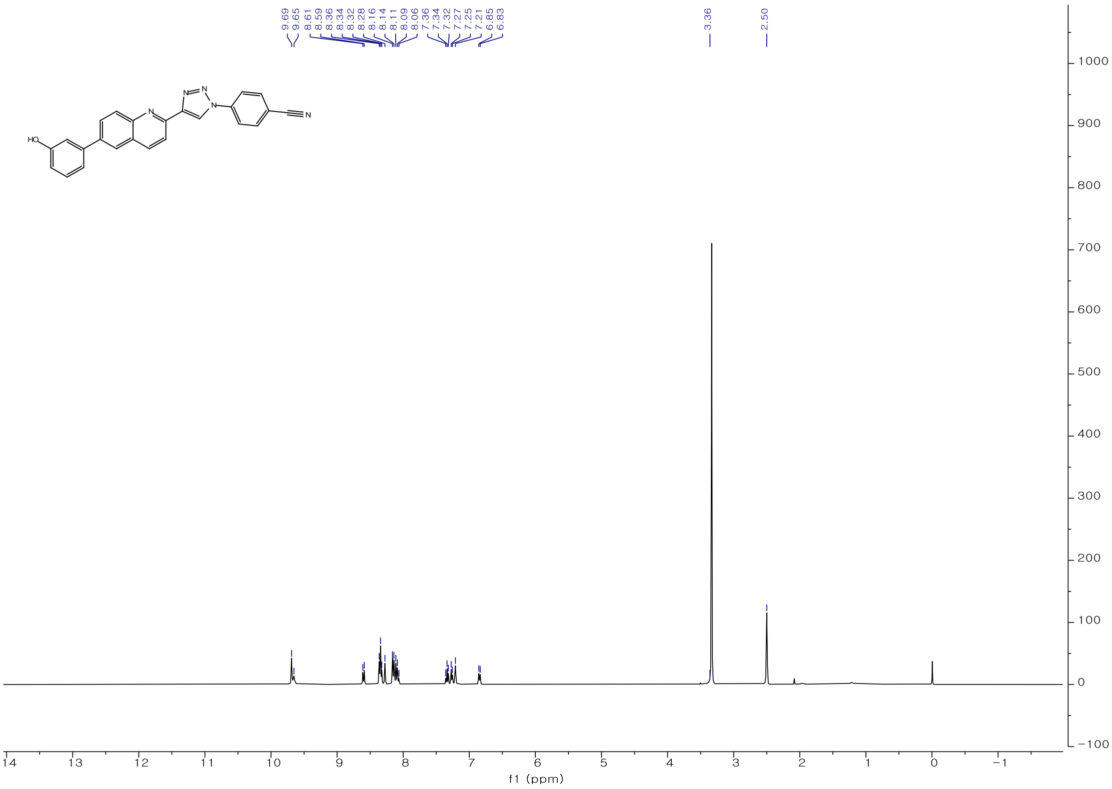
9.69
9.65
8.61
8.59
8.36
8.34
8.32
8.28
8.16
8.14
8.11
8.09
8.06
7.36
7.34
7.32
7.27
7.25
7.21
6.85
6.83
3.36
2.50
HO
N
f1 (ppm)

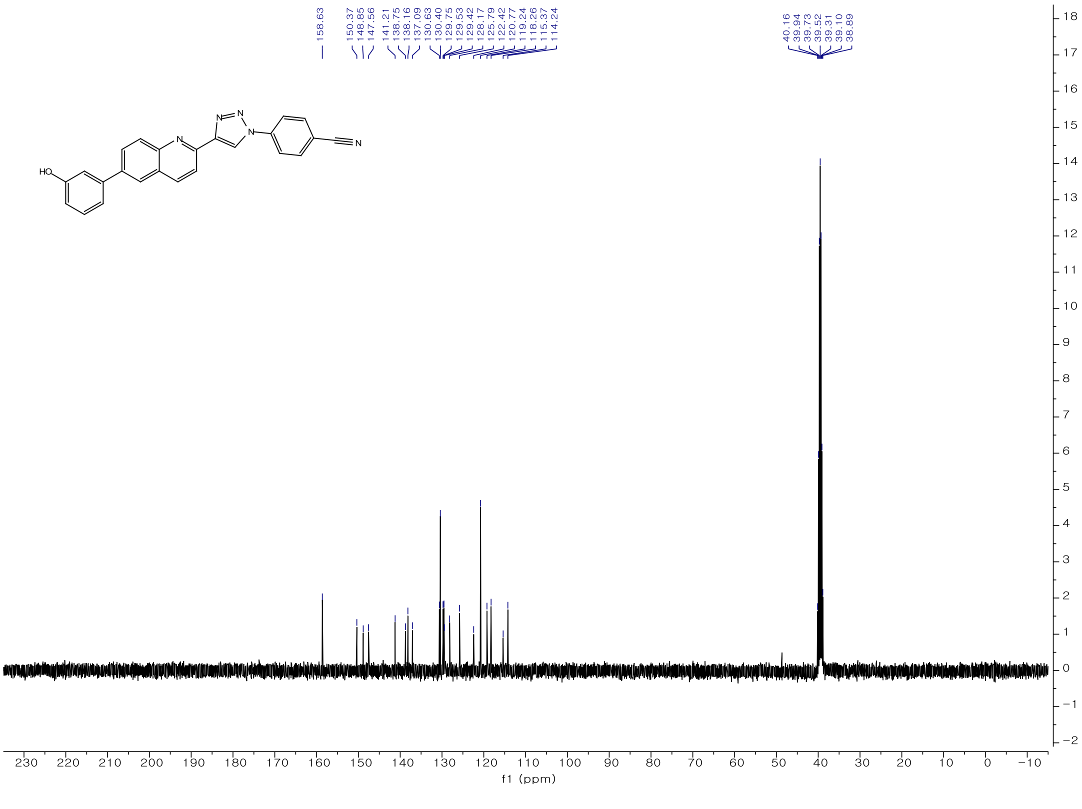
158.63
150.37
148.85
147.56
141.21
138.75
138.16
137.09
130.63
130.40
129.75
129.53
129.42
128.17
125.79
122.42
120.77
119.24
118.26
115.37
114.24
40.16
39.94
39.73
39.52
39.31
39.10
38.89
HO
N
f1 (ppm)

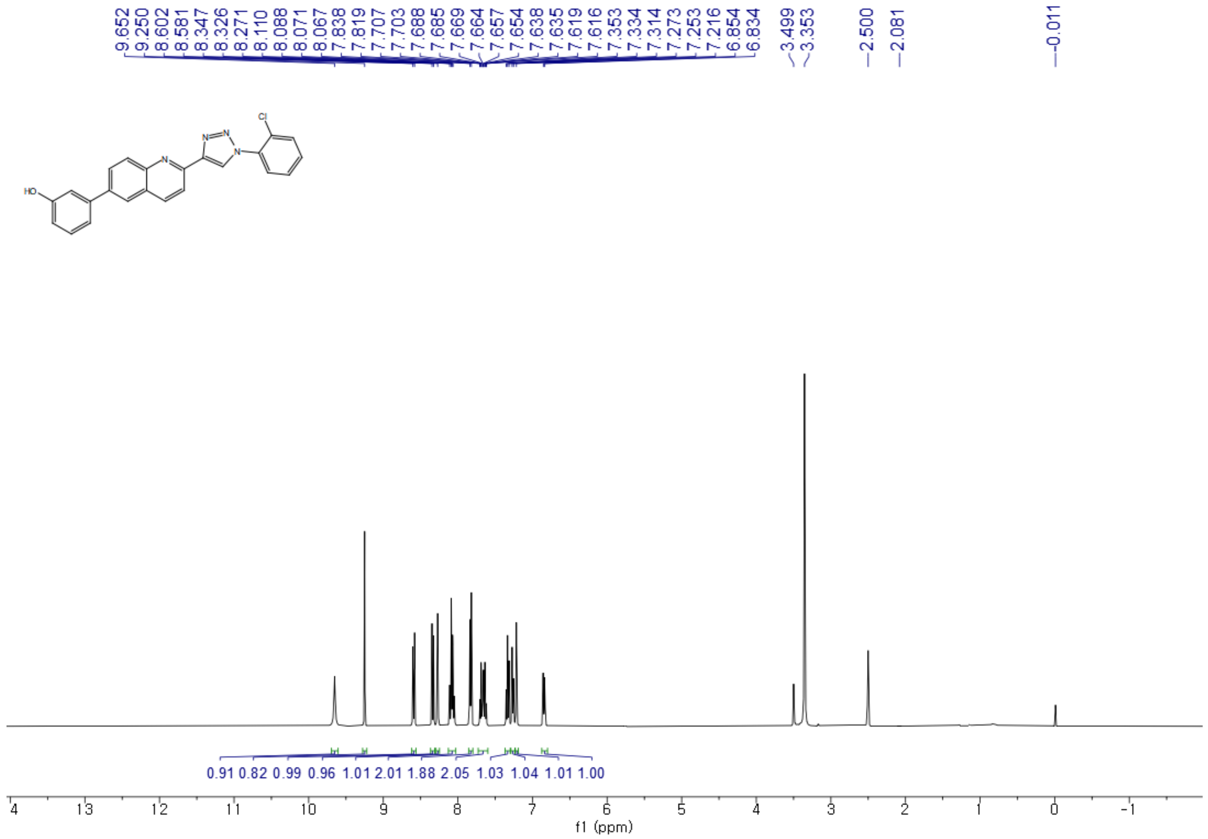

9.652
9.250
8.602
8.581
8.347
8.326
8.271
8.110
8.088
8.071
8.067
7.838
7.819
7.707
7.703
7.688
7.685
7.669
7.664
7.657
7.654
7.638
7.635
7.619
7.616
7.353
7.334
7.314
7.273
7.253
7.216
6.854
6.834
3.499
3.353
2.500
2.081
-0.011
HO
N
N
N
N
Cl
0.91 0.82 0.99 0.96 1.01 2.01 1.88 2.05 1.03 1.04 1.01 1.00
f1 (ppm)

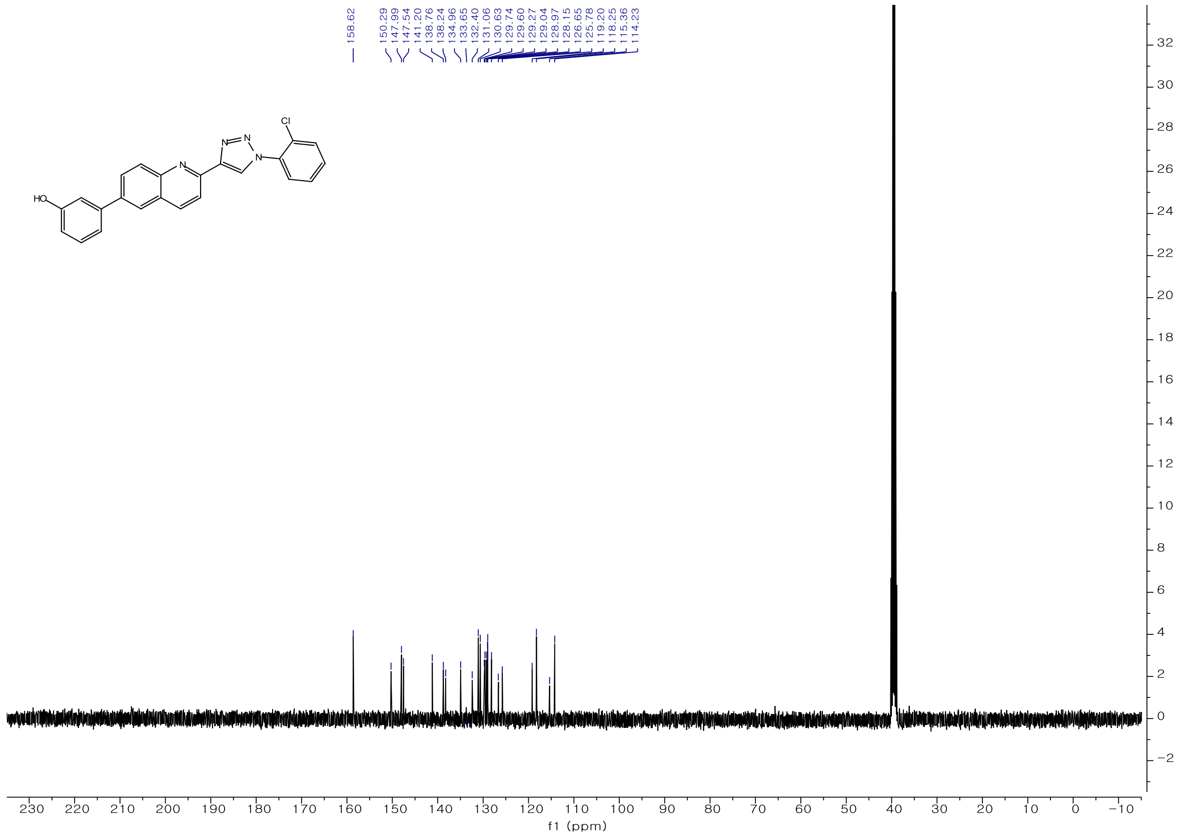

158.62
150.29
147.99
147.54
141.20
138.76
138.24
134.96
133.65
132.40
131.06
130.63
129.74
129.60
129.27
129.04
128.97
128.15
126.65
125.78
119.20
118.25
115.36
114.23
HO
N
N
N
N
Cl
f1 (ppm)

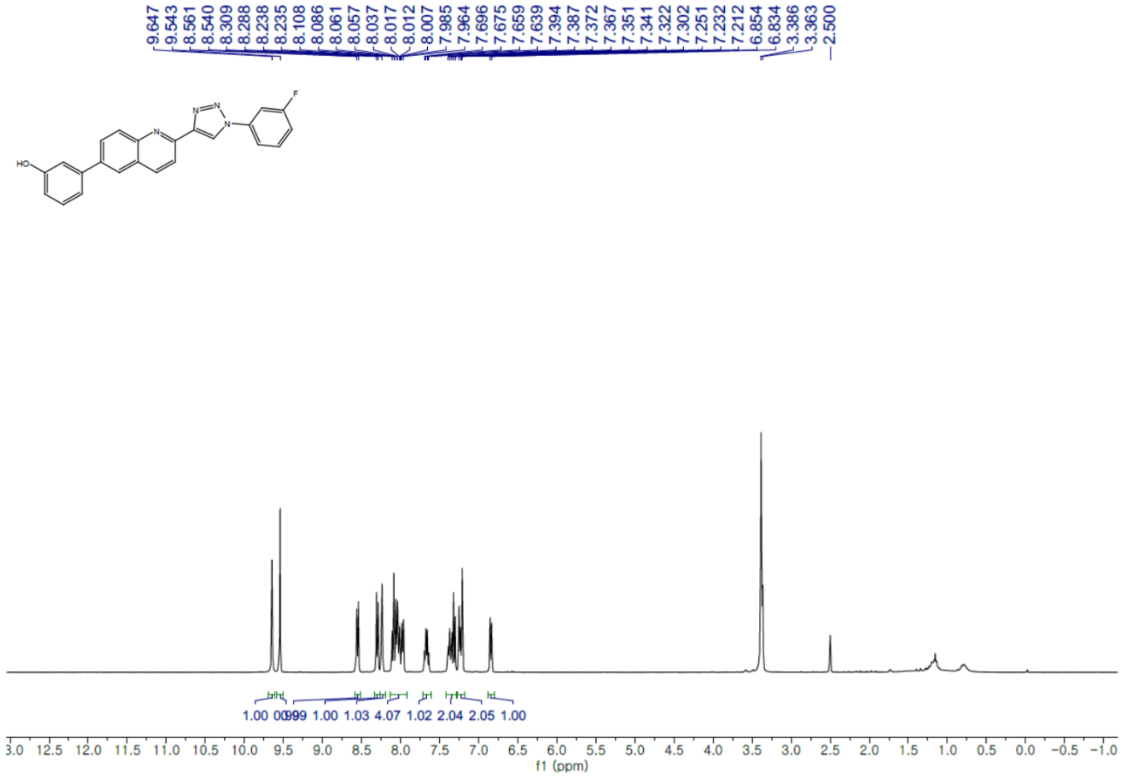

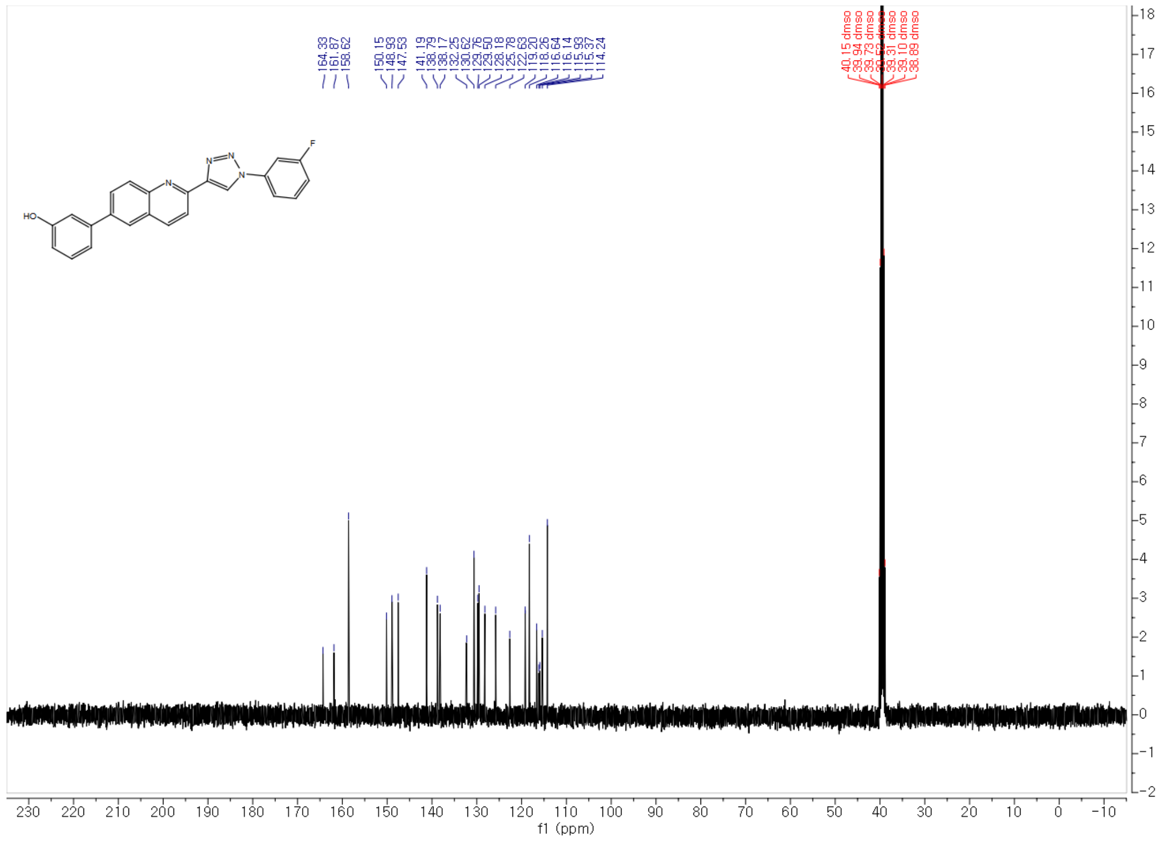

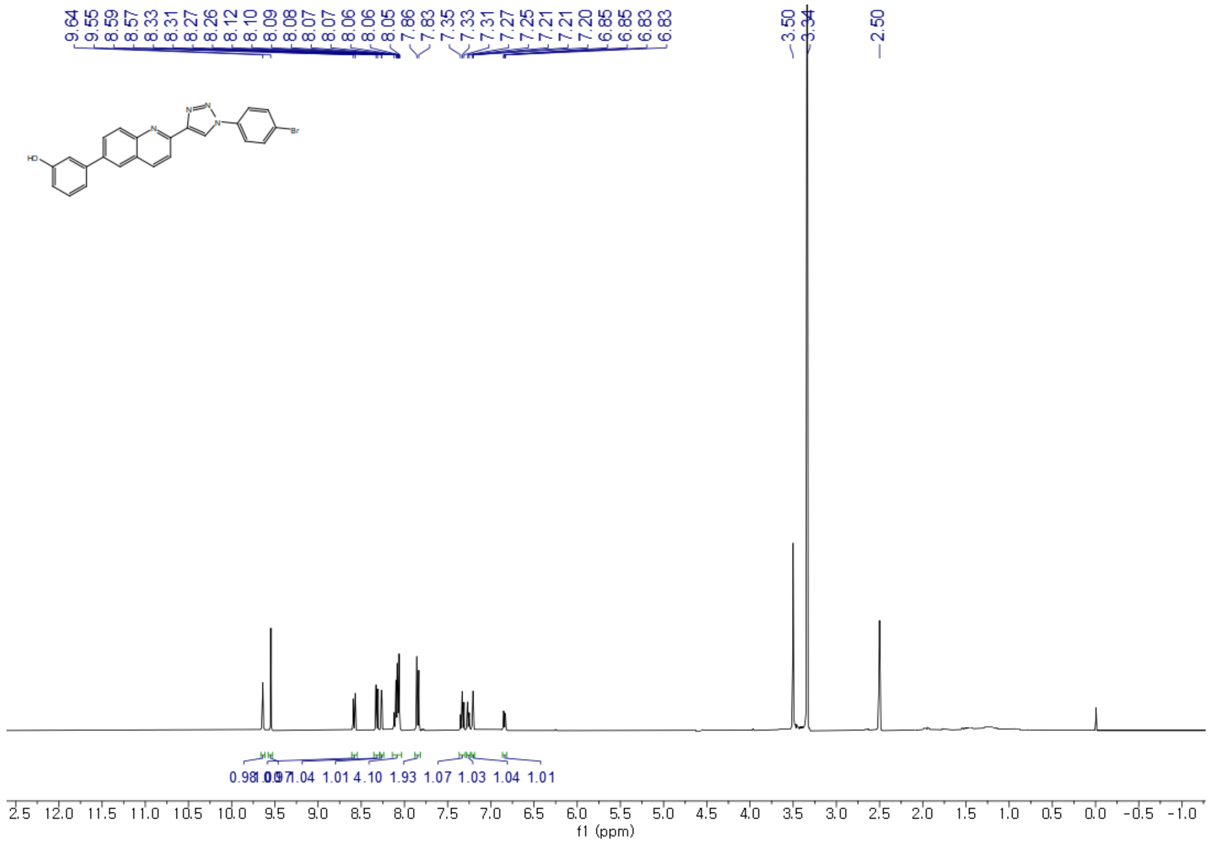

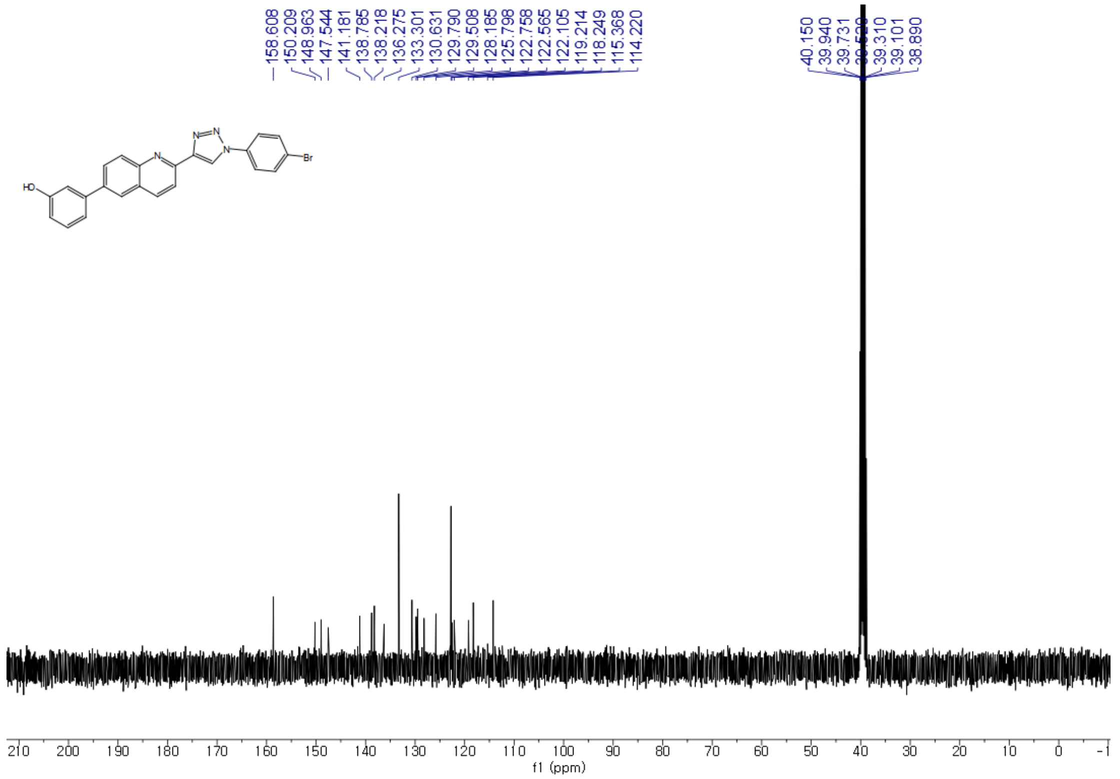

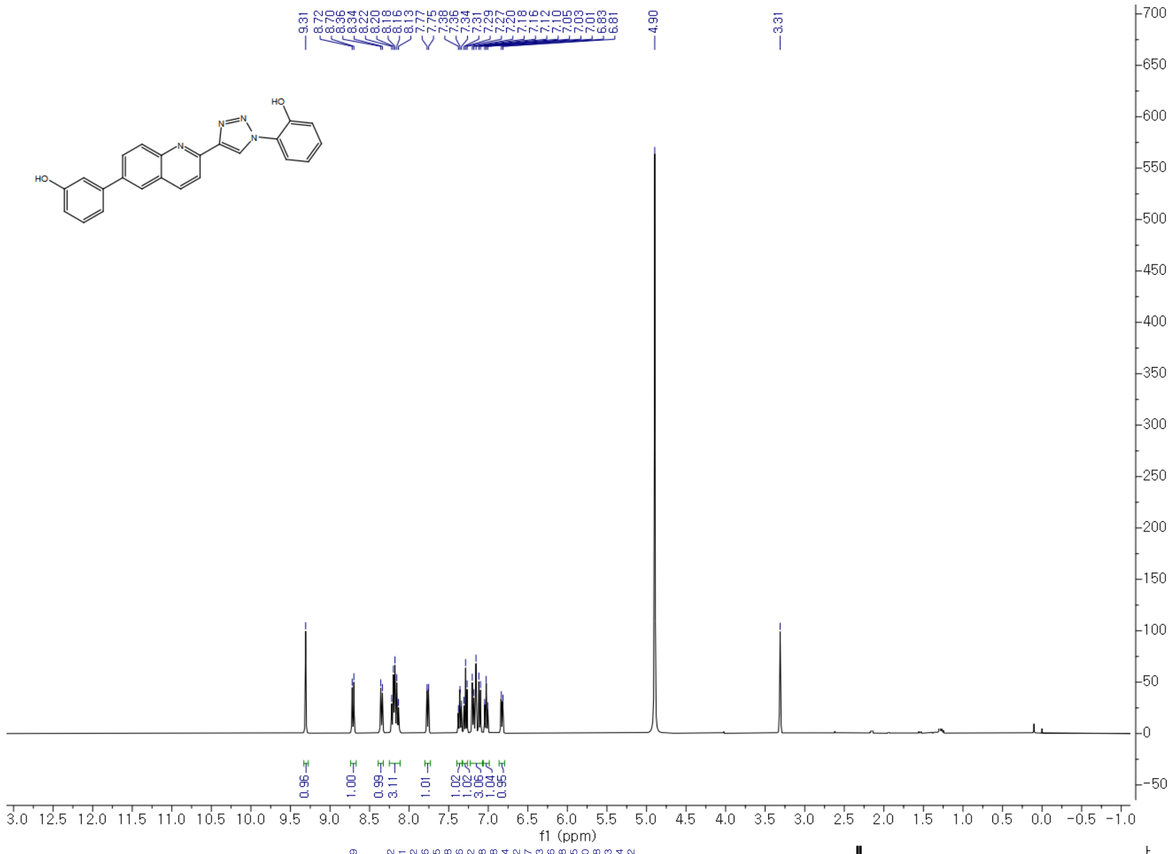

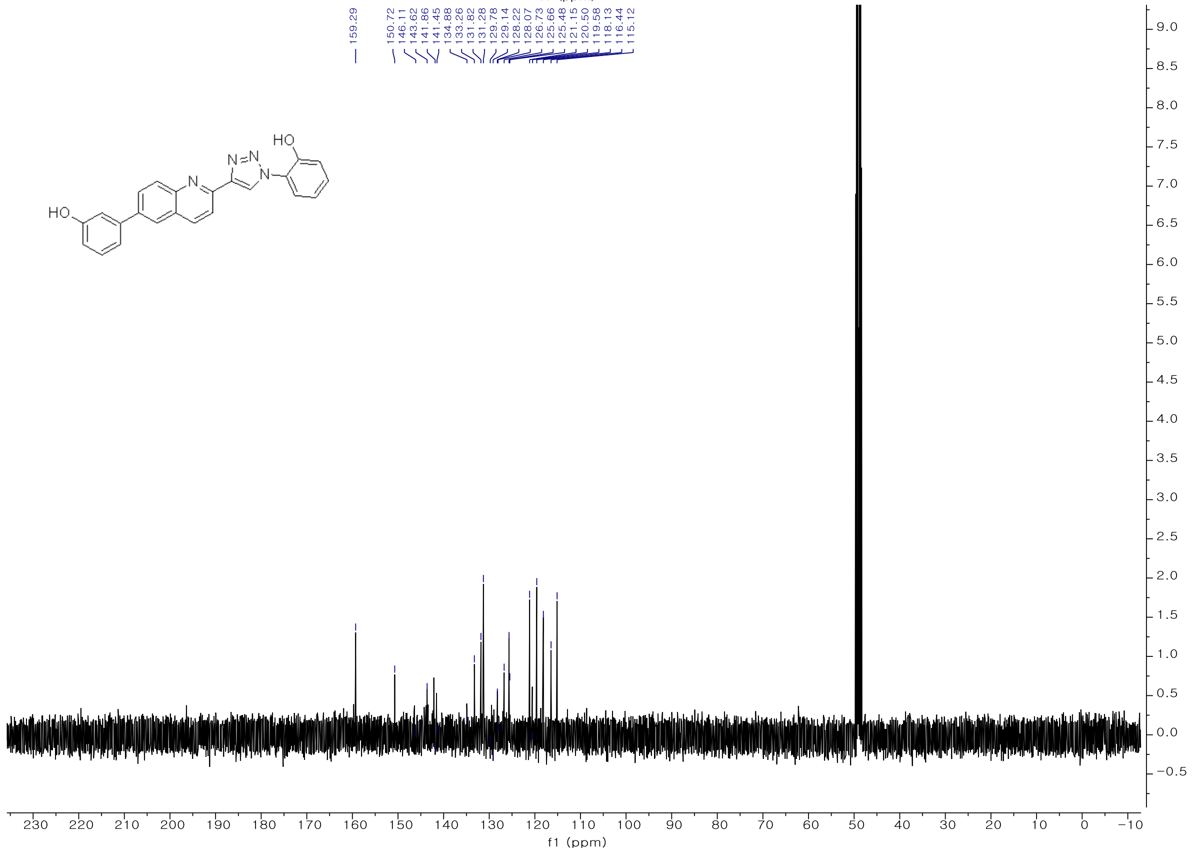

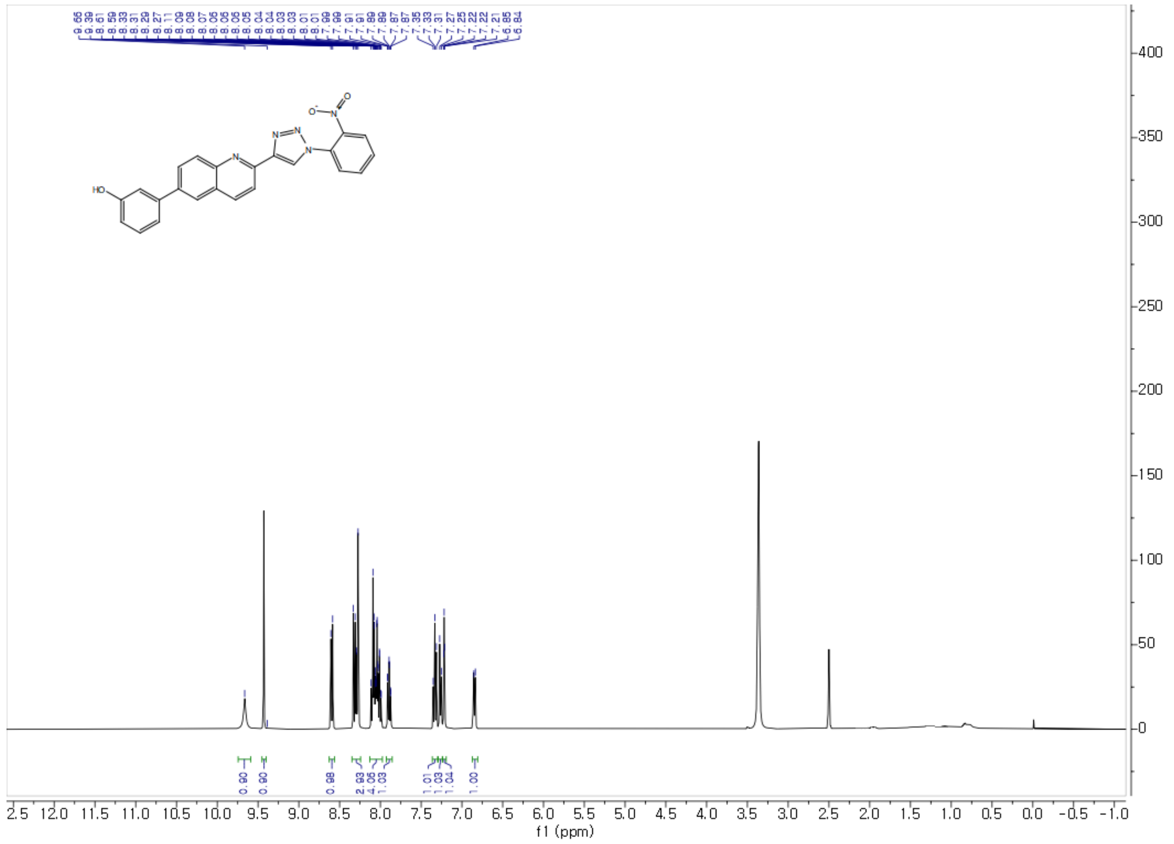

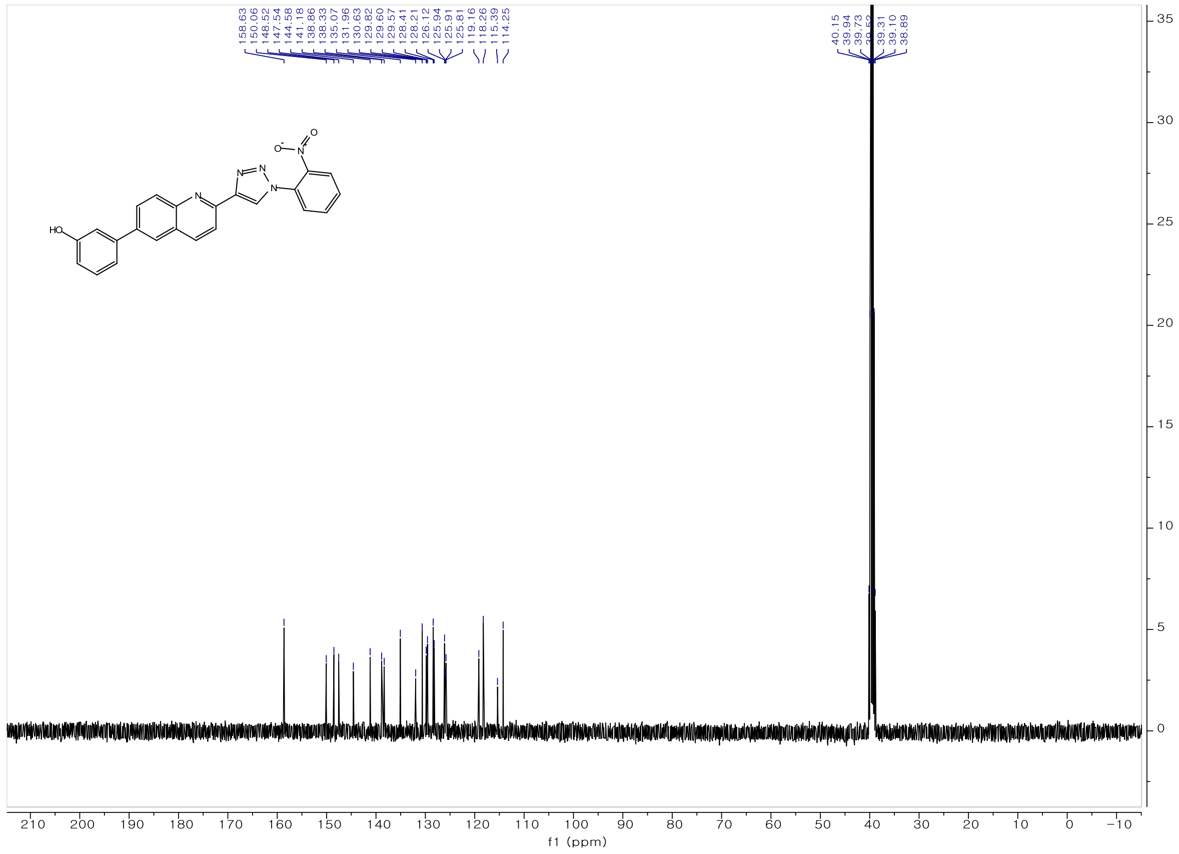

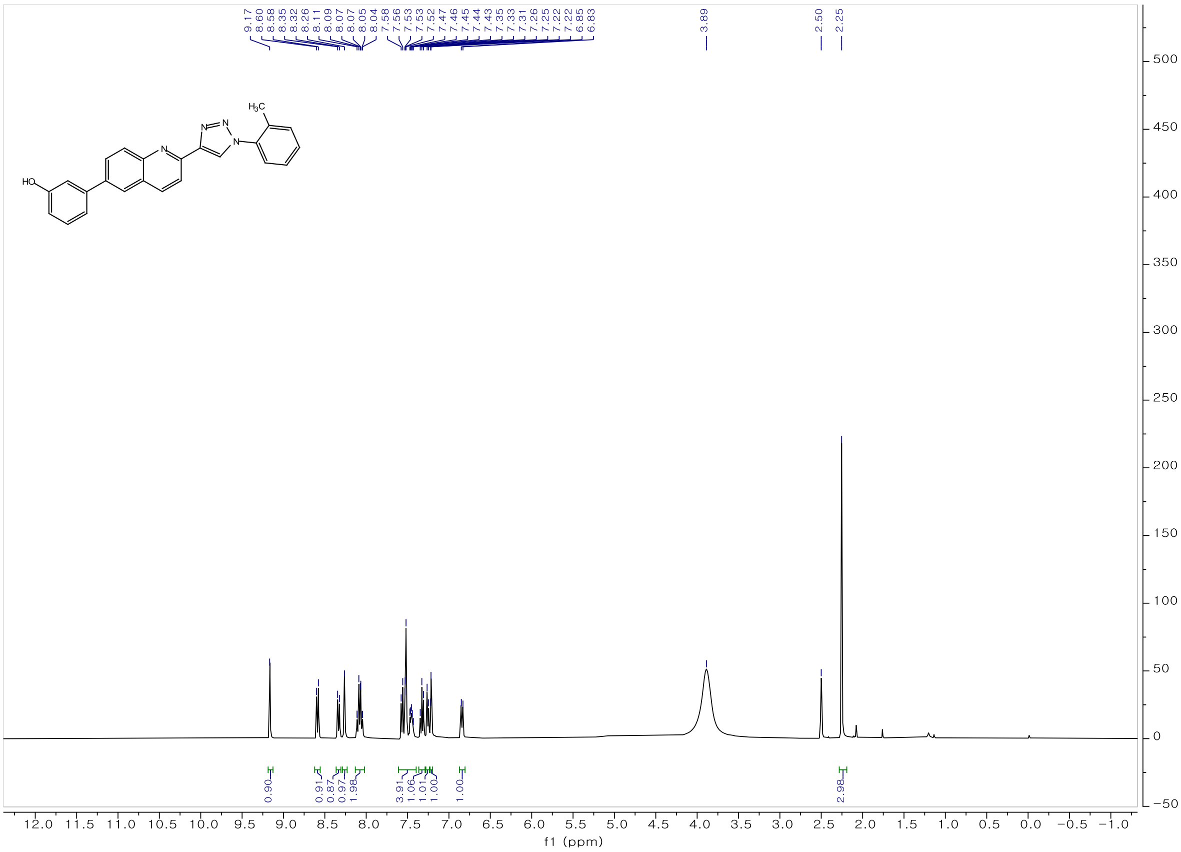

9.17
8.60
8.58
8.35
8.32
8.26
8.11
8.09
8.07
8.05
8.04
7.58
7.56
7.53
7.53
7.52
7.47
7.46
7.45
7.44
7.43
7.35
7.33
7.31
7.26
7.25
7.22
7.22
6.85
6.83
3.89
2.50
2.25
H3C
N
N=N
HO
0.90
0.91
0.87
0.97
1.98
3.91
1.06
1.01
1.00
1.00
2.98
f1 (ppm)

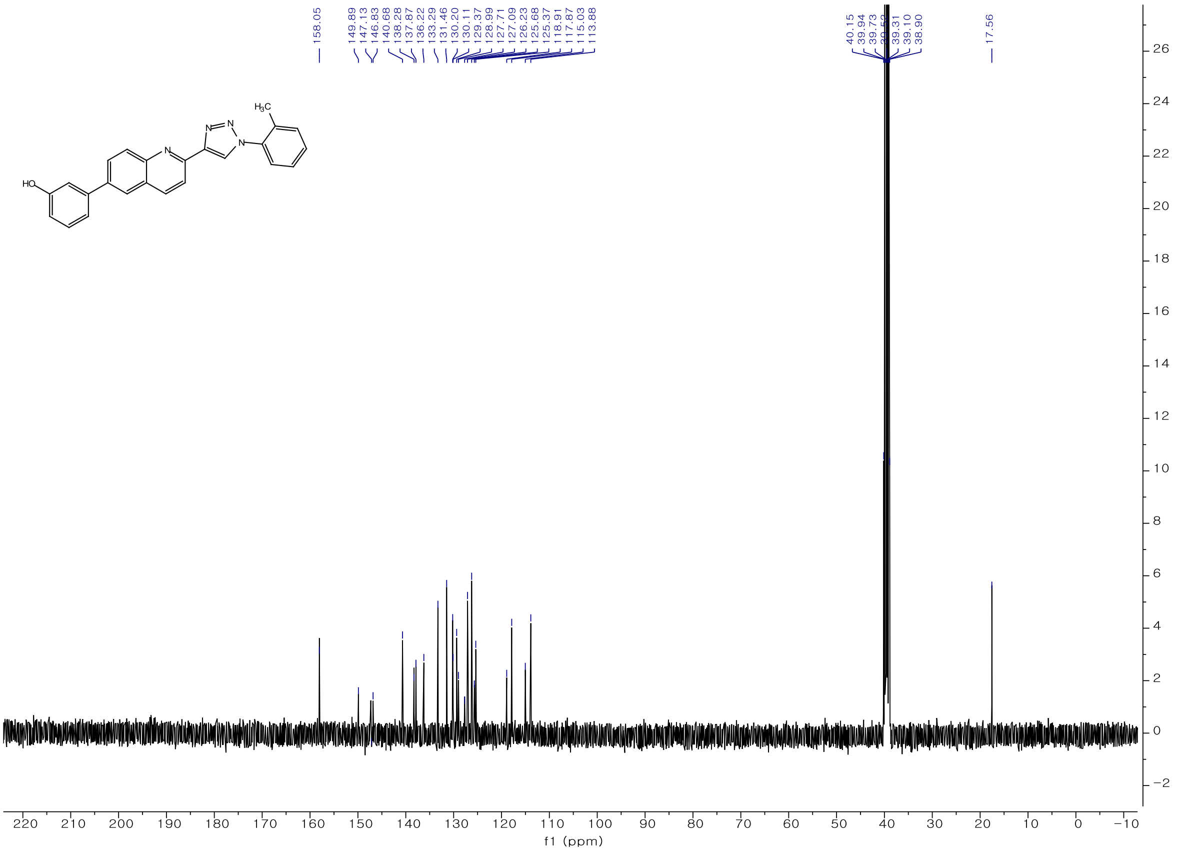

158.05
149.89
147.13
146.83
140.68
138.28
137.87
136.22
133.29
131.46
130.20
130.11
129.37
128.99
127.71
127.09
126.23
125.68
125.37
118.91
117.87
115.03
113.88
40.15
39.94
39.73
39.31
39.10
38.90
17.56
H3C
N
N=N
HO
f1 (ppm)

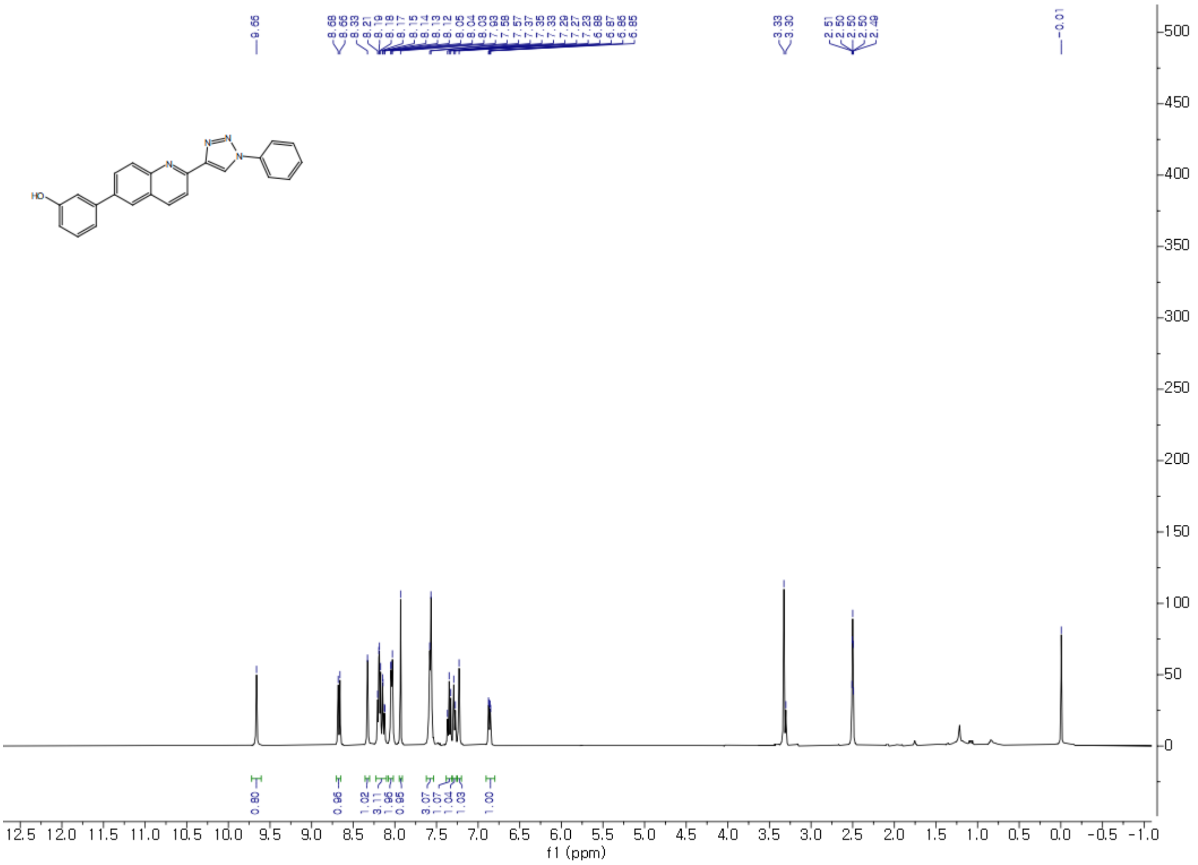
9.56
8.68
8.66
8.33
8.21
8.19
8.18
8.17
8.15
8.14
8.13
8.12
8.05
8.04
8.03
7.93
7.58
7.57
7.37
7.35
7.33
7.29
7.27
7.23
6.88
6.87
6.86
6.85
3.33
3.30
2.51
2.50
2.50
2.49
-0.01
HO
N
N
N
N
0.80
0.96
1.02
3.11
1.96
0.95
3.07
1.07
1.04
1.03
1.00
12.5 12.0 11.5 11.0 10.5 10.0 9.5 9.0 8.5 8.0 7.5 7.0 6.5 6.0 5.5 5.0 4.5 4.0 3.5 3.0 2.5 2.0 1.5 1.0 0.5 0.0 -0.5 -1.0
f1 (ppm)
500 450 400 350 300 250 200 150 100 50 0

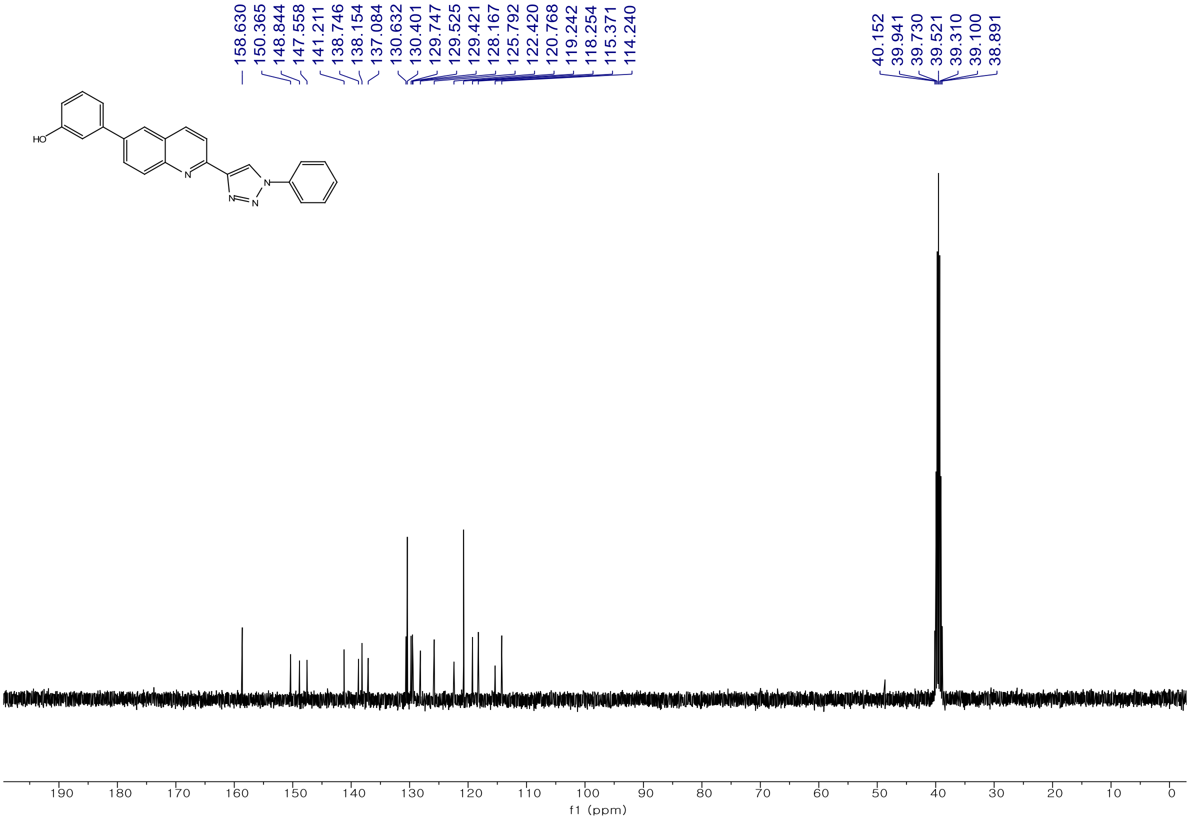
158.630
150.365
148.844
147.558
141.211
138.746
138.154
137.084
130.632
130.401
129.747
129.525
129.421
128.167
125.792
122.420
120.768
119.242
118.254
115.371
114.240
40.152
39.941
39.730
39.521
39.310
39.100
38.891
HO
N
N
N
N
190 180 170 160 150 140 130 120 110 100 90 80 70 60 50 40 30 20 10 0
f1 (ppm)

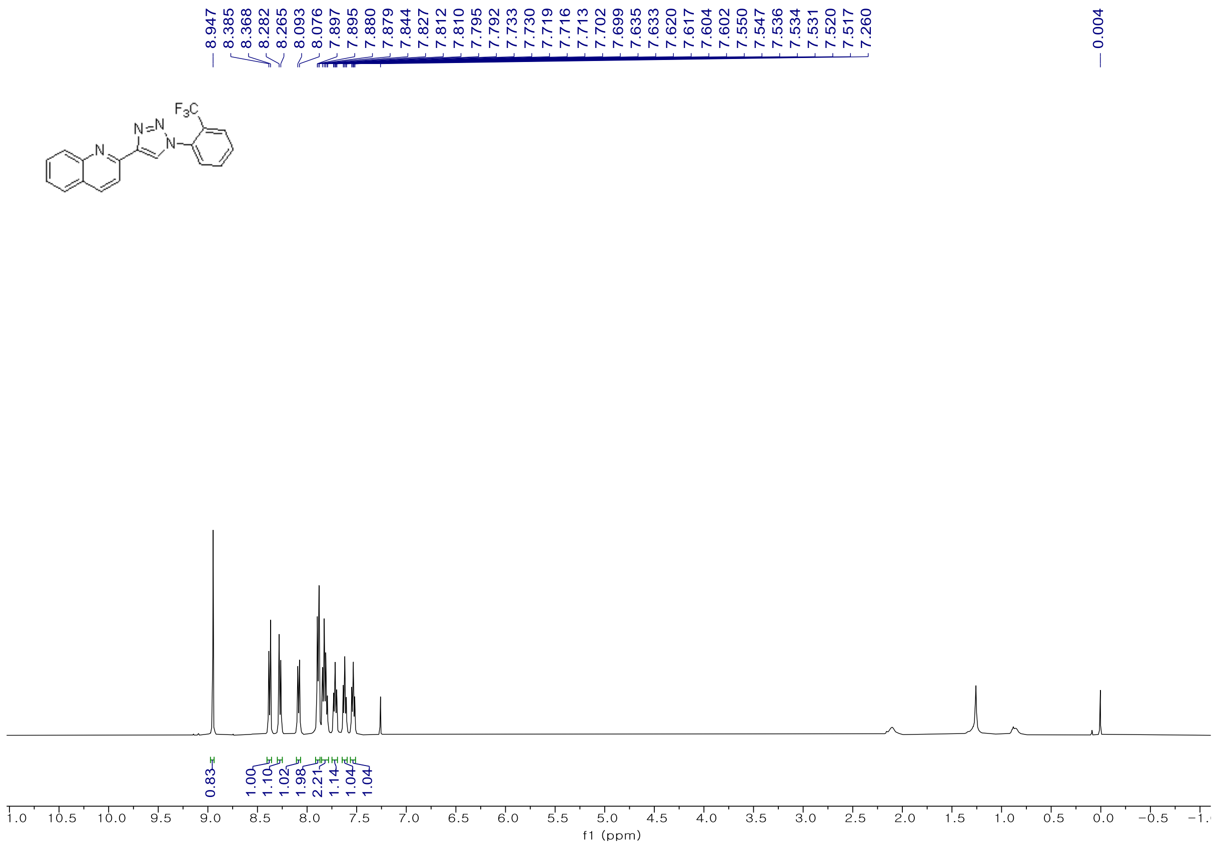
8.947
8.385
8.368
8.282
8.265
8.093
8.076
7.897
7.895
7.880
7.879
7.844
7.827
7.812
7.810
7.795
7.792
7.733
7.730
7.719
7.716
7.713
7.702
7.699
7.635
7.633
7.620
7.617
7.604
7.602
7.550
7.547
7.536
7.534
7.531
7.520
7.517
7.260
0.004
F_3C
N
N=N
N
0.83
1.00
1.10
1.02
1.98
2.21
1.14
1.04
1.04
1.0 10.5 10.0 9.5 9.0 8.5 8.0 7.5 7.0 6.5 6.0 5.5 5.0 4.5 4.0 3.5 3.0 2.5 2.0 1.5 1.0 0.5 0.0 −0.5 −1.
f1 (ppm)

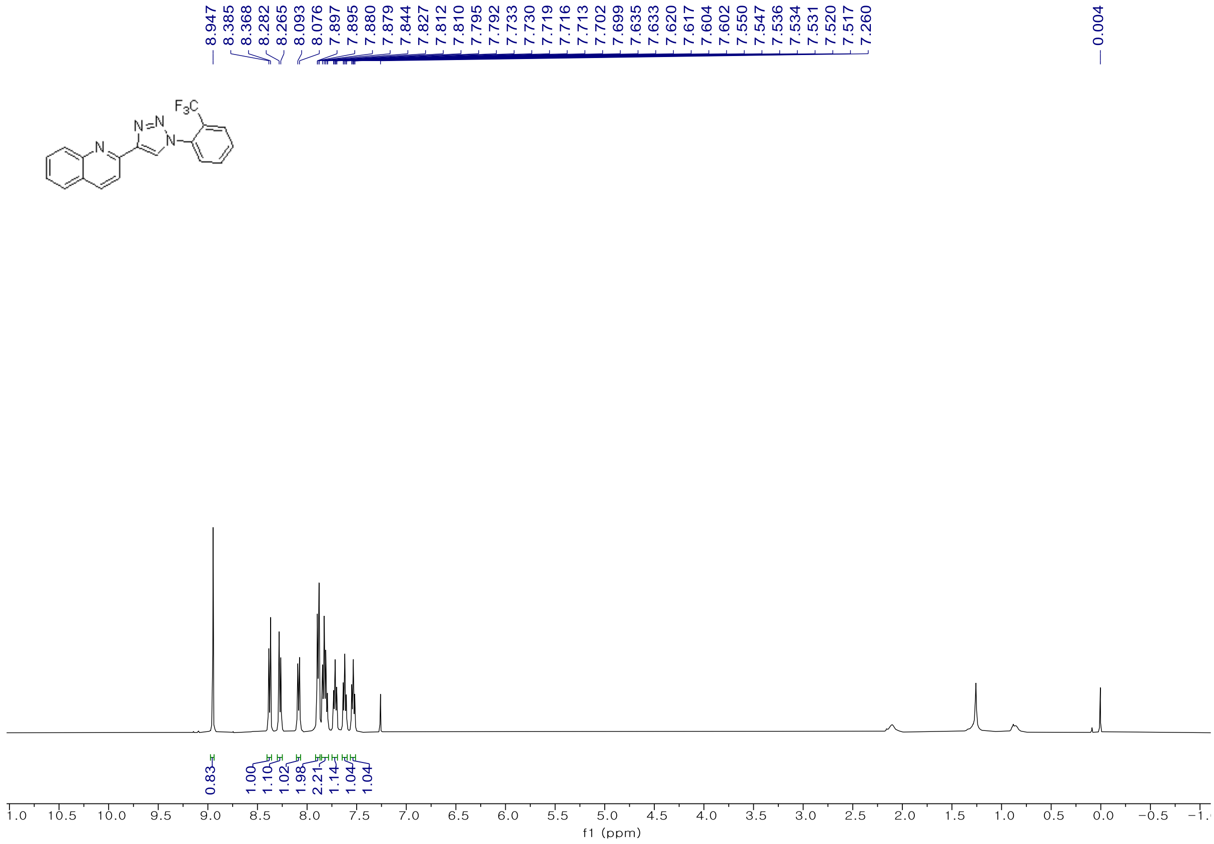
8.947
8.385
8.368
8.282
8.265
8.093
8.076
7.897
7.895
7.880
7.879
7.844
7.827
7.812
7.810
7.795
7.792
7.733
7.730
7.719
7.716
7.713
7.702
7.699
7.635
7.633
7.620
7.617
7.604
7.602
7.550
7.547
7.536
7.534
7.531
7.520
7.517
7.260
0.004
F_3C
N
N=N
N
0.83
1.00
1.10
1.02
1.98
2.21
1.14
1.04
1.04
1.0 10.5 10.0 9.5 9.0 8.5 8.0 7.5 7.0 6.5 6.0 5.5 5.0 4.5 4.0 3.5 3.0 2.5 2.0 1.5 1.0 0.5 0.0 −0.5 −1.
f1 (ppm)

V. Supplementary reference

1. Gohil VM, *et al.* Meclizine inhibits mitochondrial respiration through direct targeting of cytosolic phosphoethanolamine metabolism. *J.Biol. Chem.* **288**, 35387-35395 (2013).
