# Supplementary material for: SB2301-mediated perturbation of membrane composition in lipid droplets induces lipophagy and lipid droplets ubiquitination
Source: Commun Biol. 2023 Mar 21;6:300. doi: 10.1038/s42003-023-04682-9 (PMC10030462; doi:10.1038/s42003-023-04682-9)
Supplement: Supplementary file 2 — Description of Additional Supplementary Files [file 42003_2023_4682_MOESM2_ESM.pdf]

Description of Additional Supplementary Files

File name: Supplementary Data 1

Description: The source data behind the graphs in the paper

File name: Supplementary Video 1

Description: Imaging data observing the locations of PCYT2 and LD as a 3D block in the DMSO treatment condition

File name: Supplementary Video 2

Description: Imaging data observing the locations of PCYT2 and LD as a 3D block in the SB2301 10 µM treatment condition

File name: Supplementary Video 3

Description: Imaging data observing the locations of PCYT2 and LD as a 3D block in the SB2301 20 µM treatment condition

File name: Supplementary Video 4

Description: Imaging data observing the locations of PCYT2 and LD as a 3D block in the negative compound (**3**) treatment condition
