# Supplementary material for: SB2301-mediated perturbation of membrane composition in lipid droplets induces lipophagy and lipid droplets ubiquitination
Source: Commun Biol. 2023 Mar 21;6:300. doi: 10.1038/s42003-023-04682-9 (PMC10030462; doi:10.1038/s42003-023-04682-9)
Supplement: Supplementary file 8 — Reporting Summary [file 42003_2023_4682_MOESM8_ESM.pdf]

nature portfolio

Corresponding author(s): Seung Bum Park

Last updated by author(s): Jan 12, 2023

Reporting Summary

Nature Portfolio wishes to improve the reproducibility of the work that we publish. This form provides structure for consistency and transparency in reporting. For further information on Nature Portfolio policies, see our Editorial Policies and the Editorial Policy Checklist.

Statistics

For all statistical analyses, confirm that the following items are present in the figure legend, table legend, main text, or Methods section.

n/a	Confirmed	
☐	☒	The exact sample size (*n*) for each experimental group/condition, given as a discrete number and unit of measurement
☐	☒	A statement on whether measurements were taken from distinct samples or whether the same sample was measured repeatedly
☐	☒	The statistical test(s) used AND whether they are one- or two-sided<br>*Only common tests should be described solely by name; describe more complex techniques in the Methods section.*
☒	☐	A description of all covariates tested
☐	☒	A description of any assumptions or corrections, such as tests of normality and adjustment for multiple comparisons
☐	☒	A full description of the statistical parameters including central tendency (e.g. means) or other basic estimates (e.g. regression coefficient) AND variation (e.g. standard deviation) or associated estimates of uncertainty (e.g. confidence intervals)
☐	☒	For null hypothesis testing, the test statistic (e.g. *F*, *t*, *r*) with confidence intervals, effect sizes, degrees of freedom and *P* value noted<br>*Give P values as exact values whenever suitable.*
☒	☐	For Bayesian analysis, information on the choice of priors and Markov chain Monte Carlo settings
☒	☐	For hierarchical and complex designs, identification of the appropriate level for tests and full reporting of outcomes
☒	☐	Estimates of effect sizes (e.g. Cohen's *d*, Pearson's *r*), indicating how they were calculated

Our web collection on statistics for biologists contains articles on many of the points above.

Software and code

Policy information about availability of computer code

Data collection	Cell viability was collected by measuring the absorbance with Gen5 [BioTek]. Fluorescent gel scanning was conducted with Sapphire Capture Software [Azure Biosystems]. Fluorescent cell imaging was collected with softWoRx [Cytiva]. Immunoblot data were collected with Image Lab 4.0 [Bio-Rad]. qPCR data were collected with StepOne software v2.3 [Applied Biosystems]. No custom code was used in this paper.
Data analysis	Fluorescent cell images were analyzed with softWoRx [Cytiva], ImageJ [NIH], and InCell Developer [Cytiva]. All graphs were drawn with GraphPad Prism 8. Statistical comparison was conducted with GraphPad Prism 8. No custom code was used in this paper.

For manuscripts utilizing custom algorithms or software that are central to the research but not yet described in published literature, software must be made available to editors and reviewers. We strongly encourage code deposition in a community repository (e.g. GitHub). See the Nature Portfolio guidelines for submitting code & software for further information.

Data

Policy information about availability of data

All manuscripts must include a data availability statement. This statement should provide the following information, where applicable:

- Accession codes, unique identifiers, or web links for publicly available datasets
- A description of any restrictions on data availability
- For clinical datasets or third party data, please ensure that the statement adheres to our policy

The raw numbers for charts and graphs are available in the Source Data file. All original data of this study are available from the corresponding author upon request.

Human research participants

Policy information about studies involving human research participants and Sex and Gender in Research.

Reporting on sex and gender	N/A
Population characteristics	N/A
Recruitment	N/A
Ethics oversight	N/A

Note that full information on the approval of the study protocol must also be provided in the manuscript.

Field-specific reporting

Please select the one below that is the best fit for your research. If you are not sure, read the appropriate sections before making your selection.

☒ Life sciences ☐ Behavioural & social sciences ☐ Ecological, evolutionary & environmental sciences

For a reference copy of the document with all sections, see nature.com/documents/nr-reporting-summary-flat.pdf

Life sciences study design

All studies must disclose on these points even when the disclosure is negative.

Sample size	No sample size calculation was performed. Sample size was chosen such that statistical significance could be confidently established.
Data exclusions	No data were excluded from the analyses.
Replication	All attempts at replication were successful and included in this study.
Randomization	Not applicable, there was no experimental group allocation.
Blinding	Not applicable, there was no experimental group allocation.

Reporting for specific materials, systems and methods

We require information from authors about some types of materials, experimental systems and methods used in many studies. Here, indicate whether each material, system or method listed is relevant to your study. If you are not sure if a list item applies to your research, read the appropriate section before selecting a response.

Materials & experimental systems

n/a	Involved in the study	
☐	☒	Antibodies
☐	☒	Eukaryotic cell lines
☒	☐	Palaeontology and archaeology
☒	☐	Animals and other organisms
☒	☐	Clinical data
☒	☐	Dual use research of concern

Methods

n/a	Involved in the study	
☒	☐	ChIP-seq
☒	☐	Flow cytometry
☒	☐	MRI-based neuroimaging

Antibodies

Antibodies used	anti-LC3B (Abcam, ab51520), anti-PCYT2 (Abcam, ab126142), anti-IDH1 (Abcam, ab81653), anti-ubiquitin (Abcam, ab7780), anti-ATG5 (Abcam, ab108327), TRITC-conjugated anti-rabbit IgG secondary antibodies (ab6718), and anti-DGAT1 (ab178711), anti-GAPDH (Cell Signaling Technology, #2118), HRP-labeled anti-mouse IgG (Cell Signaling Technology, #7076), HRP-labeled anti-rabbit IgG secondary antibodies (Cell Signaling Technology, #7074), anti-ACSL4 (Santa Cruz, sc-271800), anti-WDR1 (Santa Cruz, sc-393159), Anti-SOAT1 (Novus biologicals, NB400-141), Anti-SOAT2 (Cayman, 100027), Anti-DGAT2 (Thermo, PA5-21722)
Validation	All antibodies were validated for their uses by the respective vendors.<br>anti-LC3B: https://www.abcam.com/lc3b-antibody-ab51520.html

anti-PCYT2: https://www.abcam.com/pcyt2-antibody-ab126142.html
anti-IDH1: https://www.abcam.com/idh1-antibody-ab81653.html
anti-ubiquitin: https://www.abcam.com/ubiquitin-antibody-ab7780.html
anti-ATG5: https://www.abcam.com/apg5latg5-antibody-epr17552-ab108327.html
TRITC-conjugated anti-rabbit IgG secondary antibodies: https://www.abcam.com/goat-rabbit-igg-hl-tritc-ab6718.html
anti-GAPDH: https://www.cellsignal.com/products/primary-antibodies/gapdh-14c10-rabbit-mab/2118
anti-mouse IgG: https://www.cellsignal.com/products/secondary-antibodies/anti-mouse-igg-hrp-linked-antibody/7076
anti-rabbit IgG: https://www.cellsignal.com/products/secondary-antibodies/anti-rabbit-igg-hrp-linked-antibody/7074
anti-ACSL4: https://www.scbt.com/p/acsl4-antibody-a-5
anti-WDR1: https://www.scbt.com/p/wdr1-antibody-b-10
anti-DGAT1 : https://www.abcam.com/dgat1-antibody-epr13430-ab178711.html
anti-SOAT1 : https://www.novusbio.com/products/acat-antibody_nb400-141
anti-SOAT2 : https://www.caymanchem.com/product/100027/soat-2-acat-2-polyclonal-antibody
anti-DGAT2 : https://www.fishersci.es/shop/products/anti-dgat2-polyclonal-pa521722/13269918

Eukaryotic cell lines

Policy information about cell lines and Sex and Gender in Research

Cell line source(s)	HeLa, HepG2, and AML12 were obtained from American Type Culture Collection.
Authentication	All cells were used without modification once received from the supplier, and therefore were not authenticated.
Mycoplasma contamination	All cells were tested negative for mycoplasma contamination.
Commonly misidentified lines (See ICLAC register)	No commonly misidentified cell lines were used.
